# Fully closed-loop insulin delivery in inpatients receiving nutritional support: a two-centre, open-label, randomised controlled trial

**DOI:** 10.1016/S2213-8587(19)30061-0

**Published:** 2019-05

**Authors:** Charlotte K Boughton, Lia Bally, Franco Martignoni, Sara Hartnell, David Herzig, Andreas Vogt, Maria M Wertli, Malgorzata E Wilinska, Mark L Evans, Anthony P Coll, Christoph Stettler, Roman Hovorka

**Affiliations:** a Wellcome Trust–Medical Research Council Institute of Metabolic Science, University of Cambridge, Cambridge, UK; b Wolfson Diabetes and Endocrine Clinic, Cambridge University Hospitals NHS Foundation Trust Cambridge, Cambridge, UK; c Department of Diabetes, Endocrinology, Clinical Nutrition and Metabolism, Bern University Hospital, Bern, Switzerland; d Department of Anaesthesiology and Pain Medicine, Inselspital, Bern University Hospital, Bern, Switzerland; e Department of General Internal Medicine, Bern University Hospital, Bern, Switzerland

## Abstract

**Background:**

Glucose management is challenging in patients who require nutritional support in hospital. We aimed to assess whether fully closed-loop insulin delivery would improve glycaemic control compared with conventional subcutaneous insulin therapy in inpatients receiving enteral or parenteral nutrition or both.

**Methods:**

We did a two-centre (UK and Switzerland), open-label, randomised controlled trial in adult inpatients receiving enteral or parenteral nutrition (or both) who required subcutaneous insulin therapy. Patients recruited from non-critical care surgical and medical wards were randomly assigned (1:1) using a computer-generated minimisation schedule (stratified by type of nutritional support [parenteral nutrition on or off] and pre-study total daily insulin dose [<50 or ≥50 units]) to receive fully closed-loop insulin delivery with faster-acting insulin aspart (closed-loop group) or conventional subcutaneous insulin therapy (control group) given in accordance with local clinical practice. Continuous glucose monitoring in the control group was masked to patients, ward staff, and investigators. Patients were followed up for a maximum of 15 days or until hospital discharge. The primary endpoint was the proportion of time that sensor glucose concentration was in target range (5·6–10·0 mmol/L), assessed in the intention-to-treat population. This trial is registered with ClinicalTrials.gov, number NCT01774565.

**Findings:**

Between Feb 8, 2018, and Sept 21, 2018, 90 patients were assessed for eligibility, of whom 43 were enrolled and randomly assigned to the closed-loop group (n=21) or the control group (n=22). The proportion of time that sensor glucose was in the target range was 68·4% [SD 15·5] in the closed-loop group and 36·4% [26·6] in the control group (difference 32·0 percentage points [95% CI 18·5–45·5; p<0·0001]). One serious adverse event occurred in each group (one cardiac arrest in the control group and one episode of acute respiratory failure in the closed-loop group), both of which were unrelated to study interventions. There were no adverse events related to study interventions in either group. No episodes of severe hypoglycaemia or hyperglycaemia with ketonaemia occurred in either study group.

**Interpretation:**

Closed-loop insulin delivery is an effective treatment option to improve glycaemic control in patients receiving nutritional support in hospital.

**Funding:**

Diabetes UK, Swiss National Science Foundation, National Institute for Health Research Cambridge Biomedical Research Centre, Wellcome Trust, and European Foundation for the Study of Diabetes.

## Introduction

Nutritional support with enteral or parenteral nutrition is an important component of medical care.^1^ Hyperglycaemia is common in patients receiving nutritional support in non-critical care, occurring in up to half of those receiving parenteral nutrition and in a third of those receiving enteral nutrition.^2^, ^3^ The carbohydrate content of nutritional support can exacerbate other causes of hyperglycaemia in hospital inpatients, such as metabolic responses to acute illness and medications that alter insulin sensitivity (eg, glucocorticoids).

Hyperglycaemia occurring in inpatients receiving parenteral or enteral nutrition, with or without a history of diabetes, is associated with increased morbidity and mortality.^4^, ^5^, ^6^ Observational studies^4^, ^5^, ^7^, ^8^, ^9^ have shown that the risks of infection, cardiac complications, acute renal failure, respiratory failure, and mortality increase as mean blood glucose increases in patients receiving parenteral nutrition.

Clinical practice guidelines for inpatient glucose management in non-critical care have been proposed,^10^ but implementation in patients receiving enteral or parenteral nutrition is particularly challenging. Unanticipated dislodgement of feeding tubes, temporary discontinuation of nutrition because of nausea or for administration of medication or diagnostic testing, and cycling of nutritional support with oral intake all necessitate a high level of vigilance among health-care professionals, including frequent blood glucose monitoring, regular adjustment of insulin doses, and pre-emptive administration of carbohydrates to minimise the risk of hypoglycaemia. Glucose management in this population is associated with a substantially increased workload for ward staff, and fear of hypoglycaemia often leads to suboptimal glycaemic control.

**Research in context**

**Evidence before this study**

We searched PubMed for articles published from Jan 1, 2000, up to Dec 1, 2018, using the search terms (“closed-loop” OR “artificial pancreas”) AND (“type 2 diabetes” OR “inpatient hyperglycaemia” OR “stress hyperglycaemia” OR [“hospital” AND “hyperglycaemia” AND “nutrition”]) to identify previous studies on the management of inpatient hyperglycaemia in non-critical care. Two previous inpatient studies have investigated the use of closed-loop insulin delivery in patients with type 2 diabetes in non-critical care. The larger of these randomised controlled trials was done in 136 adult patients with type 2 diabetes and compared conventional subcutaneous insulin therapy with closed-loop insulin delivery in non-critical care. Over a period of up to 15 days, the proportion of time spent in the target glucose range (5·6–10·0 mmol/L) and mean blood glucose concentrations were significantly improved with closed-loop therapy, without an increase in hypoglycaemia. Our search did not yield any previous studies that used closed-loop insulin delivery in hospital inpatients receiving nutritional support. Alternative strategies to improve glucose management in patients receiving nutritional support include the use of variable-rate intravenous insulin infusion; however, this support is associated with increased staff workload. In a non-randomised prospective study of 605 inpatients receiving parenteral nutrition, intravenous insulin (infusion therapy or added to parenteral nutrition bag) was associated with a greater risk of hypoglycaemia compared with subcutaneous or no insulin therapy. Overall glucose control was not reported. A retrospective study showed suboptimal glucose control using intravenous insulin infusion to treat parenteral nutrition-related hyperglycaemia in the non-intensive care setting. Subcutaneous insulin pump therapy titrated every 4 h has shown benefits in glycaemic variability over multiple daily insulin injections titrated every 4 h in a randomised trial involving 102 post-surgical patients receiving parenteral nutrition in hospital, but it requires substantial input from health-care providers and does not provide glucose concentration feedback. Studies have shown that insulin availability is affected by the composition of the parenteral nutrition infusion containers (adsorption with plastic-containing surfaces is much greater than with glass). The presence of a multivitamin and trace element additive is associated with much greater insulin availability than parenteral nutrition solutions without additive. The GLUCOSE-in-PN randomised trial showed no difference in mean glucose concentration achieved with continuously infused insulin in parenteral nutrition compared with subcutaneously administered long-acting insulin in non-critically ill surgical patients receiving parenteral nutrition.

**Added value of this study**

Our study is, to our knowledge, the first to assess fully automated closed-loop insulin delivery without meal bolusing in adults in non-critical care receiving enteral or parenteral nutrition (or both). We showed that an increased proportion of time was spent in the target glucose range and mean glucose was reduced with fully closed-loop insulin delivery compared with standard insulin therapy, without an increase in the time spent in hypoglycaemia or the total daily insulin dose. This study shows that fully closed-loop insulin delivery can potentially provide health-care professionals with an effective and safe clinical tool to manage hyperglycaemia in patients receiving parenteral or enteral nutritional support in hospital.

**Implications of all the available evidence**

Clinical guidelines for inpatient glucose management in non-critical care have been published. However, data from inpatient audits and studies show that implementation is challenging, particularly in patients receiving enteral or parenteral nutrition. Unanticipated dislodgement of feeding tubes, temporary discontinuation of nutrition, and cycling of nutritional support with oral intake necessitate high levels of vigilance among health-care professionals, including frequent blood glucose monitoring and regular adjustment of insulin doses. Glucose management in this population is associated with increased workload for ward staff, and fear of hypoglycaemia often leads to suboptimal glycaemic control. Further studies are needed to assess the potential of closed-loop insulin delivery to improve clinical outcomes, including morbidity and mortality, in this setting.

Closed-loop systems, which automatically deliver insulin in a glucose-responsive manner, might provide a solution to glucose management for hospital inpatients requiring nutritional support. Closed-loop systems combine real-time glucose measurements from a continuous glucose monitoring (CGM) device with a control algorithm that directs insulin delivery via an insulin pump. Evidence that closed-loop technology improves glycaemic control in inpatients with hyperglycaemia is increasing, but systematic investigations in the most challenging circumstances, such as during parenteral or enteral nutrition, have not been done.^8^, ^11^

Here we report the results of a two-centre, randomised, open-label trial of fully closed-loop insulin delivery in a diverse cohort of inpatients receiving enteral or parenteral nutrition (or both) in non-critical medical and surgical care. We hypothesised that closed-loop insulin delivery would be safe and improve glycaemic control without increasing the risk of hypoglycaemia.

## Methods

### Study design and participants

In this two-centre, open-label, randomised controlled trial, participants were recruited from non-critical care wards at Addenbrooke's Hospital (Cambridge, UK) and Bern University Hospital (Bern, Switzerland). Included patients were aged 18 years or older, had a prescription for enteral or parenteral nutrition, and had inpatient hyperglycaemia requiring subcutaneous insulin therapy during hospital admission. Exclusion criteria were type 1 diabetes, pregnancy or breastfeeding, and any physical or psychological disease or the use of one or more medication likely to interfere with the conduct of the trial or interpretation of the results. Full inclusion and exclusion criteria are available in the appendix. Inpatients were identified through hospital electronic records. Written informed consent was obtained from participants who had capacity to consent, or from relatives of participants who did not have capacity to consent (UK participants only) before the start of study-related procedures. The study protocol was approved by the local research ethics committees (East of England Central Cambridge Ethics Committee, UK; and Ethics Committee Bern, Switzerland) and regulatory authorities (Medicines and Healthcare products Regulatory Agency, UK; and Swissmedic, Switzerland). The safety of the trial was overseen by an independent data and safety monitoring board. The trial was done in accordance with the principles of the Declaration of Helsinki. The full trial protocol is available in the appendix.

### Randomisation and masking

Eligible participants were randomly assigned (1:1) to either fully closed-loop insulin delivery with faster-acting insulin aspart (closed-loop group) or conventional subcutaneous insulin therapy (control group; multiple different types of insulin regimens were used by participants and participants might have used several different insulin regimens during their time in the study as per standard clinical practice). Randomisation was done with the minimisation method, generated by Minim randomisation software,^12^ which is a biased coin approach with a probability of 0·7–0·8 of allocation to the best fitting treatment. This method aims to minimise imbalance between groups. The allocation algorithm takes into consideration the characteristics of previously allocated participants to determine the best fitting treatment group. Randomisation was stratified by the type of nutritional support (parenteral nutrition on or off) and pre-study total daily insulin dose (<50 or ≥50 units) to balance the two study groups. This was an open-label study, but the CGM receiver in the control group was modified to mask the sensor glucose measurements to the participant, investigators, and ward staff.

### Procedures

Participant bodyweight, height, and total daily insulin dose were recorded after enrolment. The study did not alter or specify the nutrition regimens prescribed by the local clinical team, and participants did not have their usual activity restricted.

In the closed-loop group, participants' usual insulin therapy and sulfonylurea medication, if prescribed, was discontinued on the day of closed-loop initialisation. All other medications were continued. A subcutaneous cannula was inserted by the investigator in the abdomen or arm for delivery of faster-acting insulin aspart (Fiasp, Novo Nordisk, Bagsværd, Denmark) by a study pump (Dana R Diabecare, Seoul, South Korea). A subcutaneous real-time CGM sensor (Freestyle Navigator II, Abbott Diabetes Care, Alameda, CA, USA) was inserted in the upper arm or abdomen by the investigator and calibrated in accordance with the manufacturer's instructions. When sensor glucose data became available, fully closed-loop glycaemic control was started by the investigator. A low glucose sensor alarm on the CGM receiver was initialised at a threshold of 3·5 mmol/L.

The FlorenceD2W-T2 automated closed-loop system consisted of a model predictive control algorithm (version 0.3.70) residing on a control algorithm device (Dell Latitude 10 tablet, Dell, Bracknell, UK) linked by a Universal Serial Bus cable to the CGM receiver (FreeStyle Navigator II; appendix). The tablet device communicated with the study pump via a Bluetooth wireless communication protocol. The control algorithm was initialised with the participant's weight and pre-study total daily insulin dose.

No prandial insulin boluses were delivered and the control algorithm did not receive data on timing or carbohydrate content of meals or enteral and parenteral feeds. Every 12 min the control algorithm calculated the required insulin infusion rate on the basis of sensor glucose measurements. The study pump was then instructed by wireless communication to adjust insulin delivery. The control algorithm adapted itself to a particular patient by updating model parameters and refining the patient's insulin requirements. The algorithm aimed to achieve glucose concentrations between 5·8 and 7·2 mmol/L and adjusted the actual target concentration depending on the accuracy of the model-based glucose predictions and prevailing glucose concentrations. Safety rules limited maximum insulin infusion and suspended insulin delivery at a sensor glucose measurement of 4·2 mmol/L or less, or when sensor glucose was rapidly decreasing. In the event of sensor failure or loss of sensor availability, the CGM receiver sounded an audible alarm that alerted the general ward or the research team. If sensor glucose data continued to be unavailable for 30 min, the study pump insulin infusion rate reverted to the preprogrammed basal rate. For longer interruptions of sensor glucose data, the control algorithm could use capillary glucose measurements to direct insulin delivery.

Point-of-care capillary glucose measurements (StatStrip Glucose Hospital Meter System, Nova Biomedical, Runcorn, UK; or Accu-Chek Inform II, Roche Diagnostics, Basel, Switzerland) were done by ward nursing staff in accordance with to local clinical practice.

At the end of the closed-loop period, participants completed a brief questionnaire providing feedback on their satisfaction with glycaemic control while on closed-loop therapy, acceptance of wearing study devices, and whether they would recommend closed-loop therapy to others. Participants' usual insulin therapy and sulfonylurea medication were restarted at the end of closed-loop use as appropriate.

In the conventional insulin therapy group, participants' insulin therapy at randomisation and other antihyperglycaemic therapies were continued throughout the study period. A CGM sensor (Freestyle Navigator II) was inserted by the investigator on the first day of the study and calibrated in accordance with the manufacturer's instructions.

Point-of-care capillary glucose measurements (StatStrip Glucose Hospital Meter System or Accu-Chek Inform II) were done by ward nursing staff. Each participant's glycaemic control was managed by the participant's clinical team in accordance with local clinical practice. The clinical team were allowed to modify and adjust participants' insulin and other antihyperglycaemic therapies and initiate additional point-of-care capillary glucose measurements as appropriate. Both centres have an inpatient diabetes service and patients in the control group referred to the diabetes team had capillary glucose reviewed every 1–2 days depending on patient complexity, with insulin doses adjusted as required. Patients not referred to the inpatient diabetes team had capillary glucose monitored and insulin dose adjusted by the clinical team.

Closed-loop insulin delivery was continued for up to 15 days or until hospital discharge. To reflect local clinical practice, in the UK, closed-loop insulin delivery was stopped if nutritional support was stopped and the diabetes team deemed that subcutaneous insulin therapy was no longer required; data collection was stopped at this point in both closed-loop and control groups. In Switzerland, data collection continued in both closed-loop and control groups up to 15 days or until discharge.

### Outcomes

The primary outcome was the proportion of time the sensor glucose measurement was in the target glucose range of 5·6–10·0 mmol/L during the study period. Secondary outcomes were the proportion of time that the sensor glucose measurement was either higher or lower than the target range; the proportion of time it was higher than 20·0 mmol/L, lower than 3·9 mmol/L, lower than 3·0 mmol/L, and lower than 2·8 mmol/L; the burden of hypoglycaemia, assessed by the area under the curve (AUC) less than 3·5 mmol/L and less than 3·0 mmol/L; the mean sensor glucose measurement; and the total daily insulin dose.

Secondary outcome measures of glycaemic variability was assessed by the SD and the coefficient of variation in sensor glucose measurements using data collected from the whole study period. The between-day coefficient of variation in sensor glucose measurements was calculated from daily mean glucose measurements (midnight to midnight). Daytime (0800–0000 h) and overnight (0000–0800 h) results were calculated for a subset of prespecified exploratory outcomes (time in target range, time above target range, mean sensor glucose measurement, the SD and the coefficient of variation in sensor glucose measurement, the between-days and between-nights coefficient of variation in sensor glucose measurements, and AUC less than 3·5 mmol/L, with the use of data from the respective periods) to limit multiple comparisons. The mean pre-meal and pre-bed capillary glucose measurements at each defined period were calculated per participant for the whole study period. Daily carbohydrate intake was calculated, and route of delivery recorded, per participant for the study period. Assessment of the experience of participants in the closed-loop group was collected at the end of the study period.

Safety endpoints were severe hypoglycaemia (<2·2 mmol/L) and clinically significant hyperglycaemia (>20 mmol/L) with ketonaemia, as determined by point-of-care capillary measurements, as well as device deficiencies, adverse events, and serious adverse events. Since the study was done in hospital inpatients, many of whom had a serious health condition or life-threatening illness at enrolment, only unanticipated adverse events and events relating to the study interventions were reported.

### Statistical analysis

This was an exploratory study in which we planned for up to 45 patients to be randomly assigned. Since previous inpatient closed-loop studies might not provide reliable information about the SD of the primary endpoint in patients receiving parenteral or enteral nutrition (or both), we applied no formal power calculation. The sample size corresponds to the sample size of a previous inpatient feasibility closed-loop randomised trial.^8^

We analysed efficacy and safety data by an intention to treat. We calculated outcomes with GStat software (version 2.3) and did statistical analyses with SPSS (version 25). An unpaired *t* test was used to compare normally distributed variables and the Mann-Whitney U test was used for highly skewed variables. We tabulated the numbers of events (a prespecified saftey outcome) that were related to a capillary glucose measurement of less than 3·5 mmol/L, less than 2·2 mmol/L, and more than 20 mmol/L in each trial group and we compared the proportion of participants with events in each group with Fisher's exact test. We report values as mean (SD) or median (IQR), unless stated otherwise. We made no allowance for multiplicity. All p values are two-tailed, and p values of less than 0·05 were deemed to indicate statistical significance.

This trial is registered with ClinicalTrials.gov, number NCT01774565.

### Role of the funding source

The funders of the study had no role in study design, data collection, data analysis, data interpretation, or writing of the report. CKB, LB, and RH had full access to all the data in the study and take responsibility for the integrity of the data and the accuracy of the data analysis. All authors made the decision to submit for publication.

## Results

**Figure 1:**
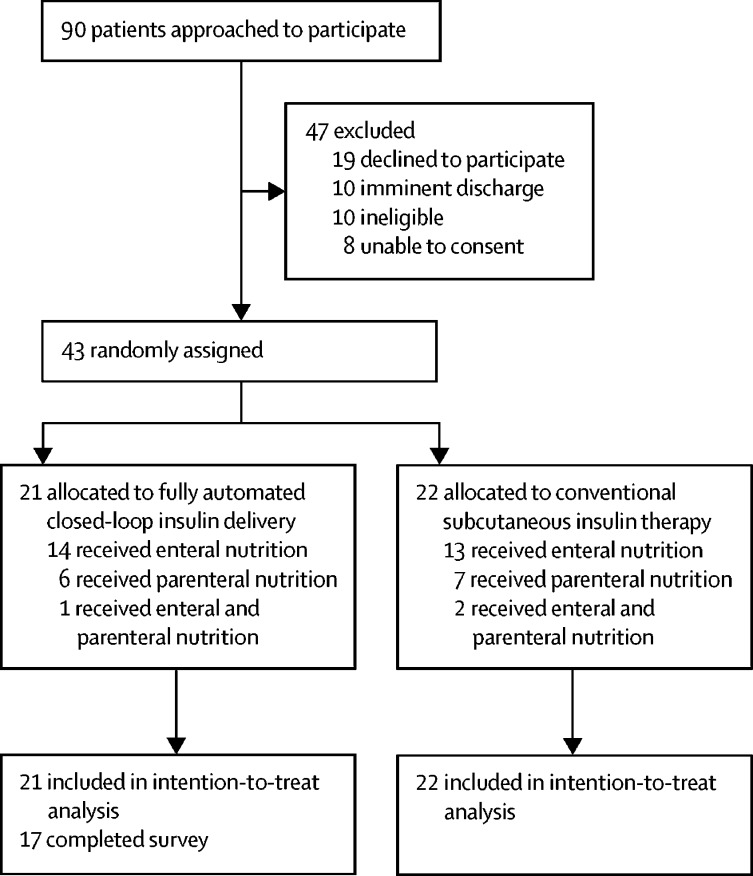
Trial profile

From Feb 8, 2018, to Sept 21, 2018, 90 participants were considered for enrolment, of whom 43 were eligible and consented; 21 were randomly assigned to the closed-loop group and 22 to the control group (figure 1). Across both study groups, roughly 80% of CGM sensors were inserted in the upper arm and 20% were inserted in the abdomen. The study period was terminated before reaching predetermined study endpoints (15 days, hospital discharge, or no requirement for insulin therapy) in five patients in the closed-loop group and ten patients in the control group. The most common reason participants stopped the study prematurely was because of discomfort wearing study devices, usually due to the additional capillary glucose measurements required for sensor calibration (n=3 in the closed-loop group; n=7 in the control group); study stopping points are outlined in the appendix.

The baseline characteristics of the closed-loop group and control group were similar (table 1). The proportion of male patients was higher in Cambridge than in Bern (90% *vs* 55%), and participants in Cambridge were younger and had higher baseline HbA_1c_ (appendix). The most common hyperglycaemia treatment at recruitment was basal-bolus insulin in 21 (49%) or basal insulin alone in ten (23%) participants (appendix). Gastrointestinal malignancy was the predominant reason for hospital admission (14 [33%] of 43 participants; appendix). The mean Charlson Comorbidity Index score was higher in the closed-loop group than in the control group (appendix). Total hospital stay was longer in the control group (median 32 days [IQR 25–47]) than in the closed-loop group (18 days [14–26]; p=0·013); however, length of hospital stay from study enrolment to discharge was similar between groups (15 days [9–30] in the control group *vs* 10 days [6–19] in the closed-loop group; p=0·13).

**Table 1:** Baseline characteristics

		**Closed-loop group (n=21)**	**Control group (n=22)**
Sex			
	Women	7 (33%)	5 (23%)
	Men	14 (67%)	17 (77%)
Age, years		66·2 (13·6)	69·1 (9·9)
BMI, kg/m^2^		27·0 (4·3)	29·3 (5·1)
HbA_1c_*		7·3% (1·6)	7·4% (1·8)
HbA_1c_, mmol/mol*		56 (17)	57 (19)
Type of diabetes			
	Type 2	10 (48%)	12 (54%)
	Pancreatic	5 (24%)	2 (9%)
	Type 2 and pancreatic	3 (14%)	7 (32%)
	Type 2 and steroids	1 (5%)	0
	Pancreatic and steroids	1 (5%)	1 (5%)
	Feed induced	1 (5%)	0
Duration of diabetes, years†		11·1 (13·9)	7·1 (7·5)
Duration on insulin therapy, years		3·2 (9·0)	3·0 (5·9)
Total daily insulin, U/kg		0·6 (0·4)	0·6 (0·3)
Total daily insulin <50 units		15 (71%)	14 (64%)
Participants recruited in Cambridge		10 (48%)	11 (50%)
Participants recruited in Bern		11 (52%)	11 (50%)

Data are n (%) or mean (SD).

* Data available for 17 participants in the closed-loop group and 19 participants in the control group.

† Data available for 20 participants in the closed-loop group and 21 participants in the control group.

The study duration, defined as the period from the first sensor reading until last sensor reading, was similar between groups (6·7 days [SD 4·0] in the closed-loop group and 6·7 days [4·3] in the control group; p=0·99; appendix) and included suspension of the study period in four patients in the closed-loop group and six patients in the control group due to intensive care transfer, surgery, or other procedures, which required transient removal of study devices. In Switzerland, all participants required insulin after nutritional support was stopped, so data collection continued in both closed-loop and control groups up to 15 days or until discharge. In the UK, four participants did not require insulin after nutritional support was stopped. Sensor glucose data were available for 97·4% (SD 3·8) of the study period (excluding suspension periods) in the closed-loop group and 91·8% (8·1) in the control group (p=0·007). Closed-loop therapy was operational for 99·2% (4·1) of the time when sensor glucose data were available.

Total daily carbohydrate intake and daily carbohydrate received via parenteral, enteral, and oral intake were similar between groups (appendix). About a third of participants received parenteral nutrition and two-thirds received enteral nutrition. Fewer than 10% received both parenteral and enteral nutrition during the study (figure 1).

The proportion of time sensor glucose concentration was in the target range (5·6–10·0 mmol/L; the primary outcome) was significantly higher in the closed-loop group (68·4% [SD 15·5]) than in the control group (36·4% [26·6]; difference 32·0 percentage points [95% CI 18·5–45·5]; p<0·0001; table 2). Mean sensor glucose concentration was significantly lower in the closed-loop group (8·5 mmol/L [1·2]) than in the control group (11·4 mmol/L [3·4]; difference 2·9 mmol/L [1·3–4·5]; p=0·001; table 2). The proportion of time spent at concentrations higher than the target range (>10·0 mmol/L) was 32·6 percentage points lower (17·8–47·3; p<0·0001) in the closed-loop group than in the control group, but the time spent at less than the target range (<5·6 mmol/L) did not differ between groups (table 2). The proportion of time spent with concentrations less than 3·9 mmol/L, less than 3·0 mmol/L, and less than 2·8 mmol/L, and burden of hypoglycaemia (as measured by AUC less than 3·5 mmol/L), were low and similar between groups (table 2). Glycaemic control outcomes by site and by nutritional support regimen are shown in the appendix.

**Table 2:** Primary and secondary outcomes

		**Closed-loop group (n=21)**	**Control group (n=22)**	**p value**
Proportion of time spent at glucose concentration				
	5·6–10·0 mmol/L*	68·4% (15·5)	36·4% (26·6)	<0·0001
	>10·0 mmol/L	22·2% (15·7)	54·8% (29·7)	<0·0001
	>20·0 mmol/L	0·3% (0·7)	6·2% (13·9)	0·06
	<5·6 mmol/L	9·3% (6·3)	8·7% (10·3)	0·82
	<3·9 mmol/L	0·5% (0·0–1·5)	0·5% (0·0–2·9)	0·74
	<3·0 mmol/L	0·0% (0·0–0·2)	0·0% (0·0–0·8)	0·37
	<2·8 mmol/L	0·0% (0·0–0·1)	0·0% (0·0–0·5)	0·31
Mean glucose concentration, mmol/L		8·5 (1·2)	11·4 (3·4)	0·001
SD of glucose concentration, mmol/L		2·3 (0·8)	3·4 (1·4)	0·003
Coefficient of variation of glucose concentration		26·7% (6·2)	29·7% (9·7)	0·24
Between-days coefficient of variation of sensor glucose		14·7% (6·1)	20·0% (11·7)	0·07
AUC_Day_ <3·5 mmol/L (mmol/L × min)		1·7 (0·0–24·9)	3·6 (3·5–113·5)	0·58
AUC_Day_ <3·0 mmol/L (mmol/L × min)		0·0 (0·0–4·4)	0·0 (0·0–20·1)	0·39
Total daily insulin dose (units)		53·9 (26·9–82·6)	40·3 (28·8–52·7)	0·47
Capillary glucose concentrations†				
	Pre-breakfast, mmol/L (0500–0800 h)	8·4 (1·6)	11·1 (4·5)	0·014
	Pre-lunch, mmol/L (1100–1300 h)	8·7 (2·1)	13·6 (4·8)	0·0003
	Pre-evening meal, mmol/L (1600–1900 h)	7·9 (1·2)	11·3 (4·4)	0·002
	Pre-bed, mmol/L (2100–0000 h)	9·1 (1·7)	10·7 (4·9)	0·17
Number of events with capillary glucose <3·5 mmol/L		4	9	..
Number of participants with capillary glucose <3·5 mmol/L		2 (10%)	5 (23%)	0·25
Number of capillary glucose measurements per 24 h		4·1 (0·9)	5·3 (1·6)	0·01

Data are mean (SD) or median (IQR), unless otherwise specified. AUC_Day_=area under the curve per 24-h period.

* Primary endpoint.

† Pre-breakfast n=21 for the closed-loop group, n=21 for the control group; pre-lunch n=21 (closed-loop) and n=20 (control); pre-evening meal n=21 (closed-loop) and n=22 (control); and pre-bed n=20 (closed-loop) and n=21 (control).

**Figure 2:**
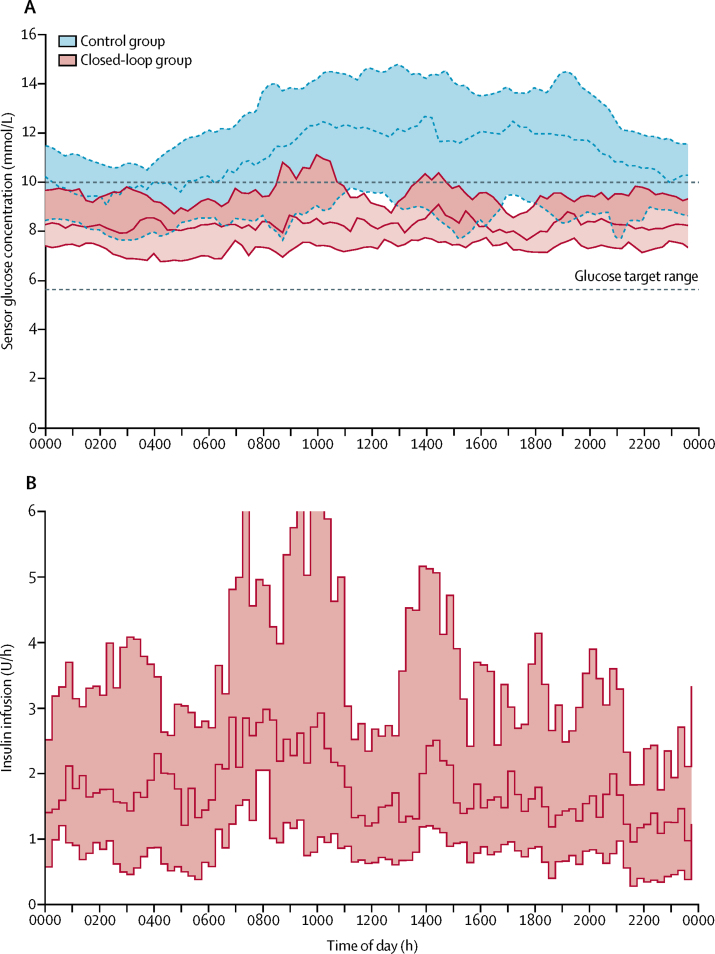
Sensor glucose concentration and insulin delivery profiles (A) Sensor glucose concentration during closed-loop and control interventions from midnight to midnight (lines indicate median, shaded areas indicate IQRs). The glucose target range is 5·6–10·0 mmol/L. (B) Algorithm-directed insulin delivery during closed-loop intervention (line indicates median, shaded area indicates IQR).

Total daily insulin delivery did not differ significantly between groups (table 2). Glycaemic variability during closed-loop therapy, as measured by SD of sensor glucose measurement, was significantly reduced compared with conventional insulin therapy (table 2). Pre-bed capillary glucose concentration did not differ significantly between groups, but pre-breakfast, pre-lunch, and pre-evening meal glucose concentrations were higher in the control group than in the closed-loop group (table 2). 24 h sensor glucose data and insulin delivery profiles are shown in figure 2.

The proportion of time that overnight (0000–0800 h) and daytime (0800–0000 h) sensor glucose concentration was in the target range was significantly higher in the closed-loop group than in the control group (overnight difference 30·1 percentage points [95% CI 15·3–44·8; p=0·0002]; daytime difference 32·1 percentage points [18·4–45·7; p<0·0001]; table 3). Mean sensor glucose was significantly lower in the closed-loop group than in the control group overnight and during daytime periods (table 3). SD of sensor glucose overnight and during daytime periods was significantly lower in the closed-loop group than in the control group (table 3). Overnight and daytime burden of hypoglycaemia, as measured by AUC less than 3·5 mmol/L, did not differ significantly between groups (table 3).

**Table 3:** Overnight (0000–0800 h) and daytime (0800–0000 h) outcomes

	Closed-loop group (n=20)*	**Control group (n=22)**	**p value**
**Overnight period (0000–0800 h)**			
Proportion of time spent at glucose concentration 5·6–10·0mmol/L	72·7% (17·2)	42·6% (28·7)	0·0002
Mean glucose concentration, mmol/L	8·3 (1·3)	10·3 (3·3)	0·013
SD of glucose concentration, mmol/L	1·8 (0·8)	2·9 (1·3)	0·002
Coefficient of glycaemic variability	20·8% (7·6)	28·6% (12·8)	0·021
Between night coefficient of glycaemic variability	17·7% (8·2)	26·0% (14·2)	0·026
AUC_Day_ <3·5 mmol/L (mmol/l × min)	0·0 (0·0–0·0)	0·0 (0·0–4·5)	0·52
Insulin dose, units	17·9 (8·7–28·2)	..	..
**Daytime period (0800–0000 h)**			
Proportion of time spent at glucose concentration 5·6–10·0 mmol/L	66·5% (16·4)	34·4% (26·6)	<0·0001
Mean glucose concentration, mmol/L	8·6 (1·2)	11·8 (3·6)	0·0005
SD of glucose concentration, mmol/L	2·4 (0·8)	3·3 (1·3)	0·018
Coefficient of glycaemic variability	27·9% (6·5)	27·9% (9·3)	0·98
Between night coefficient of glycaemic variability	15·5% (5·3)	20·6% (11·5)	0·07
AUC_Day_ <3·5 mmol/L (mmol/l × min)	1·7 (0·0–20·3)	0·0 (0·0–43·0)	0·91
Insulin dose, units	35·2 (17·8–57·2)	..	..

Data are mean (SD) or median (IQR), unless otherwise specified. AUC_Day_=area under the curve per 24-h period.

* One participant had no overnight data because they were in the study <12 h before transfer to intensive care unit.

Closed-loop experience questionnaires were completed by 17 (81%) of 21 participants in the closed-loop group (three participants were unable to complete the questionnaire due to impaired cognition, and one participant self-discharged before receiving the questionnaire). Overall, 100% (17/17) of those who completed the questionnaire stated that their diabetes treatment in hospital during the study was as expected or better than expected and 100% reported that having their glycaemia controlled automatically by the closed-loop system was as expected or better than expected (appendix). All 17 participants who completed the questionnaire would recommend the system to a friend or family if they were admitted to hospital.

Four hypoglycaemic episodes with capillary glucose concentration of less than 3·5 mmol/L, confirmed by point-of-care measurements, occurred in the closed-loop group (two participants), compared with nine episodes in the control group (five participants). These episodes were treated with oral or enteral carbohydrates in accordance with local guidelines by the ward staff, with intravenous dextrose required in one episode (2·9 mmol/L; in the closed-loop group) because of a reduced level of consciousness and a blocked nasogastric tube. As per protocol, one patient in the closed-loop group received supplemental insulin in accordance with the instruction of the study team when sensor glucose was greater than 18 mmol/L for more than 1 h.

No episodes of severe hypoglycaemia or significant hyperglycaemia with ketonaemia occurred in either group. Two severe adverse events (one cardiac arrest and one episode of acute respiratory failure resulting in death) occurred during the study (one case of acute respiratory failure in the closed-loop group and one case of cardiac arrest in the control group); neither was related to the study (table 4). No adverse events were related to study devices in either group (discontinuation due to intolerance of devices was not considered an adverse event). Device deficiencies occurred in five patients in the closed-loop group and two patients in the control group. The most common device deficiency was premature sensor failure (n=2 in the closed-loop group; n=2 in the control group). On each occasion the sensor was replaced within hours.

**Table 4:** Safety findings

		**Closed-loop group (n=21)**	**Control group (n=22)**
Number of severe hypoglycaemic events*		0	0
Number of significant hyperglycaemic events†		2	42
Number of participants with significant hyperglycaemic events†		2 (10%)	9 (41%)
Number of adverse events			
	Serious adverse events (not study related)	1	1
	Non-serious adverse events (not study related)	5	4
Number of participants with adverse events		4 (19%)	4 (18%)
Number of device deficiencies		5	2
Number of participants with device deficiencies		5 (24%)	2 (9%)

Data are n or n (%).

* Severe hypoglycaemia is defined as capillary glucose concentration of less than 2·2mmol/L or the patient requiring assistance from another member of health-care staff.

† Significant hyperglycaemia is defined as capillary glucose concentration greater than 20 mmol/L.

## Discussion

In this randomised controlled trial, we have shown that fully closed-loop insulin delivery provided significantly better glycaemic control than did conventional subcutaneous insulin therapy in inpatients on enteral or parenteral nutrition (or both) in non-critical care. The proportion of time that the sensor glucose concentration was in the target range was significantly greater in the closed-loop group than in the control group, and the mean sensor glucose and SD were significantly lower in the closed-loop group. Improved glycaemic control was achieved without increasing the risk of hypoglycaemia and without an increase in total daily insulin dose.

Recommended glucose targets in non-critical care are not attainable by many health-care institutions with conventional insulin therapy.^13^, ^14^ In this study, the benefits of fully closed-loop glycaemic control suggest that this technology has the potential to substantially improve management of hyperglycaemia in one of the most challenging inpatient populations. The advantage of fully closed-loop insulin delivery in this population is the instantaneous and continually adaptive glucose-responsive modulation of insulin delivery, which can accommodate the glycaemic challenges associated with enteral and parenteral nutrition—such as unanticipated dislodgement of feeding tubes, temporary discontinuation of nutrition, and cycling of enteral nutrition with oral intake. The closed-loop algorithm continually adapts to changing insulin needs during the day and between days, allowing increases in time spent in the target glucose range and lowering of mean glucose concentration without increasing time spent in hypoglycaemia. We used faster-acting insulin aspart (Fiasp) because of its rapid onset and offset, to further improve the safety and efficacy of the closed-loop insulin delivery system. Conventional insulin therapy is less responsive to alterations in insulin requirements, and despite frequent blood glucose monitoring and regular adjustment of insulin doses, tighter glycaemic control with conventional therapy is associated with increased risk of hypoglycaemia and related adverse medical outcomes.^15^, ^16^, ^17^

Our study shows that overnight insulin requirements were higher in patients receiving nutritional support than in previous closed-loop inpatient studies,^8^, ^11^ probably due to the timing and duration of the feed compared with patients with standard oral intake. This increased insulin requirement coupled with fear among health-care professionals of hypoglycaemia and associated adverse outcomes—especially overnight—might contribute to the prevalent hyperglycaemia in the control group. Indeed, time spent in hypoglycaemia was low in the control group, but was similar between groups.

Alternative approaches to improving glucose management in this population include use of variable-rate intravenous insulin infusion; however, this method requires considerable staff workload with up to 2 h per 24 h period spent monitoring blood glucose and adjusting insulin infusion rates, precluding widespread use on general wards.^18^ Subcutaneous insulin pump therapy adjusted with a titration algorithm every 4 h has shown some benefit over multiple daily insulin injections in patients receiving parenteral nutrition, reducing mean blood glucose and measures of glycaemic variability, but it does not provide feedback and adaptability and requires substantial input from health-care providers.^19^ The addition of insulin to parenteral nutrition is associated with variable availability of insulin and does not accommodate cycling of enteral nutrition with oral intake.^20^

Between-night sensor glucose variability was reduced with closed-loop therapy use compared with conventional therapy, but between-day sensor glucose variability did not differ significantly between groups. This result reflects the advantage of frequent modulation of insulin delivery based on sensor glucose, trading variability of insulin delivery with consistency in glucose concentration. Previous studies have shown that higher glycaemic variability (measured by the SD and mean daily change in blood glucose) increases the risk of mortality in inpatients receiving parenteral nutrition.^21^

Identifying populations most likely to benefit from closed-loop glycaemic control is likely to be important for widespread adoption of this technology in the inpatient setting. Our findings expand on the results of a randomised trial that investigated use of a fully closed-loop system in an unselected cohort of inpatients with hyperglycaemia.^11^ Despite potentially more challenging glucose management in our study due to the nutrition regimens prescribed, participants in the closed-loop group spent more than 16 h each day in the target glucose range, an additional 7·5 h compared with the control group.

Acceptability of closed-loop systems in the inpatient setting has been positive and the system has been designed to limit effects on mobility. Many inpatients require tubes and attachments for treatment of comorbidities other than diabetes. Future closed-loop systems will have a substantially smaller device burden. Implementation of closed-loop systems in the inpatient setting will require training for ward nursing staff. Set-up, initialisation, and day-to-day management of the closed-loop system do not require specialist input. However, administration of intravenous insulin, which is currently undertaken by ward nursing staff, is more complex and has a higher risk of dosing error and health consequences. Future studies should assess to what extent the adoption of closed-loop technology adds to the complexity of staff duties. Surveillance for efficacy and safety will be possible with the remote monitoring capabilities of newer closed-loop platforms, allowing remote review of multiple patients on closed-loop systems across the hospital. Limitations to adoption include clinical inertia and time required to train health-care professionals. Studies showing clinical benefit from closed-loop glycaemic control should help to overcome these barriers.

The strength of this study is the use of a novel approach to address the need for effective and safe management of hyperglycaemia in one of the most challenging inpatient populations. In the control group, glycaemic control was managed in accordance with local hospital guidelines, reflecting real-world practice. The two-country design allowed the safety and efficacy of fully closed-loop glycaemic control to be assessed across different health-care systems, supporting generalisability. The closed-loop system used commercially available components, allowing ease of use in different health-care settings and potentially allowing accelerated adoption of the system for future widespread clinical use.

The study design was pragmatic because of the heterogeneity of the study population, allowing individualised treatment intensification on a patient-by-patient basis in the control group and reflecting usual clinical practice in the two centres. Insulin regimens and the frequency of glucose monitoring varied within the control group. Both centres had an inpatient diabetes service and patients in the control group referred to the diabetes team had capillary glucose measurements reviewed every 1–2 days depending on patient complexity, with insulin doses adjusted as required, whereas patients not referred to the inpatient diabetes team had capillary glucose monitoring and insulin dose adjustment done by the clinical team. The target glucose range varies for hospital inpatients in non-critical care. Tight glycaemic control is associated with increased mortality in the intensive care setting because of an increased risk of hypoglycaemia.^22^ However, the target glucose concentration range of 5·6–10·0 mmol/L used in our study is more appropriate on general wards, where the nursing staff-to-patient ratio is lower, and high workloads often prevent more frequent glucose monitoring.

This study has limitations, including the small number of patients. Sensor glucose data availability was higher in the closed-loop group than in the control group. In the closed-loop group, the research team were notified of any loss of connectivity between sensor and receiver device by an alarm, whereas the glucose sensor was masked in the control group and such connectivity interruptions were not detected, leading to reduced sensor glucose data.

In conclusion, we have shown that fully closed-loop insulin delivery in inpatients receiving parenteral or enteral nutrition (or both) is safe and significantly improves glycaemic control without increasing the risk of hypoglycaemia compared with conventional insulin therapy in non-critical care. Further studies are required to assess whether closed-loop glycaemic control can translate into improved clinical outcomes and is cost-effective in particular patient cohorts to support widespread adoption by health-care systems.

## Data sharing

No additional data are available.

## References

[1] Correia MI, Waitzberg DL (2003). The impact of malnutrition on morbidity, mortality, length of hospital stay and costs evaluated through a multivariate model analysis. Clin Nutr.

[2] Pleva M, Mirtallo JM, Steinberg SM (2009). Hyperglycemic events in non-intensive care unit patients receiving parenteral nutrition. Nutr Clin Pract.

[3] Pancorbo-Hidalgo PL, Garcia-Fernandez FP, Ramirez-Perez C (2001). Complications associated with enteral nutrition by nasogastric tube in an internal medicine unit. J Clin Nurs.

[4] Pasquel FJ, Spiegelman R, McCauley M (2010). Hyperglycemia during total parenteral nutrition. Diabetes Care.

[5] Cheung NW, Napier B, Zaccaria C, Fletcher JP (2005). Hyperglycemia is associated with adverse outcomes in patients receiving total parenteral nutrition. Diabetes Care.

[6] Gonzalez Infantino CA, Gonzalez CD, Sanchez R, Presner N (2013). Hyperglycemia and hypoalbuminemia as prognostic mortality factors in patients with enteral feeding. Nutrition.

[7] Olveira G, Tapia MJ, Ocón J (2013). Parenteral nutrition-associated hyperglycemia in non-critically ill inpatients increases the risk of in-hospital mortality (multicenter study). Diabetes Care.

[8] Thabit H, Hartnell S, Allen JM (2017). Closed-loop insulin delivery in inpatients with type 2 diabetes: a randomised, parallel-group trial. Lancet Diabetes Endocrinol.

[9] Leelarathna L, English SW, Thabit H (2013). Feasibility of fully automated closed-loop glucose control using continuous subcutaneous glucose measurements in critical illness: a randomized controlled trial. Crit Care.

[10] Umpierrez GE, Hellman R, Korytkowski MT (2012). Management of hyperglycemia in hospitalized patients in non-critical care setting: an endocrine society clinical practice guideline. J Clin Endocrinol Metab.

[11] Bally L, Thabit H, Hartnell S (2018). Closed-loop insulin delivery for glycemic control in noncritical care. N Engl J Med.

[12] Evans S, Day S, Royston P Minim: minimisation program for allocating patients to treatments in clinical trials. https://www-users.york.ac.uk/~mb55/guide/minim.htm.

[13] Health and Social Care Information Centre (HSCIC) (2017). National Diabetes Inpatient Audit (NaDIA) 2017—National Report.

[14] Swanson CM, Potter DJ, Kongable GL, Cook CB (2011). Update on inpatient glycemic control in hospitals in the United States. Endocr Pract.

[15] Christensen MB, Gotfredsen A, Norgaard K (2017). Efficacy of basal-bolus insulin regimens in the inpatient management of non-critically ill patients with type 2 diabetes: a systematic review and meta-analysis. Diabetes Metab Res Rev.

[16] Murad MH, Coburn JA, Coto-Yglesias F (2012). Glycemic control in non-critically ill hospitalized patients: a systematic review and meta-analysis. J Clin Endocrinol Metab.

[17] Nirantharakumar K, Marshall T, Kennedy A, Narendran P, Hemming K, Coleman JJ (2012). Hypoglycaemia is associated with increased length of stay and mortality in people with diabetes who are hospitalized. Diabet Med.

[18] Aragon D (2006). Evaluation of nursing work effort and perceptions about blood glucose testing in tight glycemic control. Am J Crit Care.

[19] Li FF, Zhang WL, Liu BL (2018). Management of glycemic variation in diabetic patients receiving parenteral nutrition by continuous subcutaneous insulin infusion (CSII) therapy. Sci Rep.

[20] McCulloch A, Bansiya V, Woodward JM (2018). Addition of insulin to parenteral nutrition for control of hyperglycemia. JPEN J Parenter Enteral Nutr.

[21] Farrokhi F, Chandra P, Smiley D (2014). Glucose variability is an independent predictor of mortality in hospitalized patients treated with total parenteral nutrition. Endocr Pract.

[22] NICE-SUGAR Study Investigators (2009). Intensive versus conventional glucose control in critically ill patients. N Engl J Med.

