# Supplementary material for: Fully closed-loop insulin delivery in inpatients receiving nutritional support: a two-centre, open-label, randomised controlled trial
Source: Lancet Diabetes Endocrinol. 2019 May;7(5):368–77. doi: 10.1016/S2213-8587(19)30061-0 (PMC6467839; doi:10.1016/S2213-8587(19)30061-0)
Supplement: Supplementary appendix [file mmc1.pdf]

THE LANCET
Diabetes & Endocrinology

Supplementary appendix

This appendix formed part of the original submission and has been peer reviewed. We post it as supplied by the authors.

Supplement to: Boughton C K, Bally L, Martignoni F, et al. Fully closed-loop insulin delivery in inpatients receiving nutritional support: a two-centre, open-label, randomised controlled trial. *Lancet Diabetes Endocrinol* 2019; published online March 29. http://dx.doi.org/10.1016/S2213-8587(19)30061-0.

Supplementary Appendix

Table of Contents

Table S1.	Baseline characteristics by site	page 2
Table S2.	Admission details	page 3
Table S3.	Hyperglycaemia treatment at randomization	page 4
Table S4.	Nutrition support	page 5
Table S5.	Glucose control by site	page 6
Table S6.	Nutrition support regimens and glucose control	page 7
Table S7.	Utility assessment	page 8
Figure S1.	Automated fully closed-loop insulin delivery prototype (FlorenceD2W-T2) used in the study	page 9
Figure S2.	Participant study stopping points	page 10
Figure S3.	Participants' responses to the feedback questionnaire about closed-loop insulin delivery	page 11
Protocol	Study protocol (Phase 3 applies to the present work)	
SAP	Statistical analysis plan	

Table S1. Baseline characteristics by site.

	**Cambridge (n=21)**	**Bern (n=22)**
Male sex – no./total no [%]	19/21 [90]	12/22 [55]
Age (years)	63·3 (14·8)	71·9 (5·8)
BMI (kg/m^2)	28·2 (5·0)	28·1 (4·8)
HbA1c (%)*	8·5 (1·9)	6·6 (1·0)
HbA1c (mmol/mol)*	68 (21)	49 (11)
Duration of diabetes (years)**	6·3 (6·5)	11·4 (13·7)
Duration on insulin therapy (years)	1·5 (2·7)	4·6 (10·0)
Total daily insulin (U/kg/24h)	0·7 (0·3)	0·5 (0·3)

Data presented as mean (SD)
Age and baseline HbA1c were significantly different between centres.
* Data available for 14 Cambridge participants and 22 Bern participants
** Data available for 19 Cambridge participants and 22 Bern participants

Table S2. Admission details.

	Closed-loop (n=21)	Control (n=22)
**Reason for admission**		
**Sepsis**		
Respiratory	0 (0)	1 (4·5)
Abdominal	0 (0)	1 (4·5)
Other	3 (14·3)	0 (0)
**Renal**		
Acute Kidney Injury	1 (4·8)	1 (4·5)
Other	0 (0)	1 (4·5)
**Malignancy**		
Gastrointestinal	6 (28·6)	8 (36·4)
Other	0 (0)	1 (4·5)
**Gastrointestinal**		
Liver disease	2 (9·5)	0 (0)
Pancreatitis	3 (14·3)	1 (4·5)
Obstruction	1 (4·8)	2 (9·1)
Other	2 (9·5)	3 (13·6)
**Respiratory**		
COPD exacerbation	0 (0)	1 (4·5)
**Neurological**		
Stroke	3 (14·3)	1 (4·5)
Other	0 (0)	1 (4·5)
**Admission type**		
Medical	7 (33·3)	8 (36·4)
Surgical	14 (66·7)	14 (63·6)
Emergency	13 (61·9)	12 (54·5)
Elective	8 (38·1)	10 (45·5)
Charlson Comorbidity Index	8·1 (4·0)	5·5 (2·2)

Data presented as n (%), Charlson Comorbidity Index data is mean (SD)
Charlson Comorbidity Index ranges from 0-33 with lower scores representing less comorbidity burden
COPD - chronic obstructive pulmonary disease

Table S3. Hyperglycaemia treatment at randomization.

	**Closed-loop (n=21)**	**Control (n=22)**
Insulin		
Basal alone	3 (14·3)	7 (31·8)
Basal bolus	10 (47·6)	11 (50·0)
Biphasic	1 (4·8)	2 (9·1)
Bolus alone	6 (28·6)	2 (9·1)
Biphasic + bolus	1 (4·8)	0 (0)
Metformin	3 (14·3)	1 (4·5)
SGLT2 inhibitor	1 (4·8)	1 (4·5)
DPP4 inhibitor	2 (9·5)	0 (0)
GLP-1 receptor agonist	0 (0)	1 (4·5)

Data are n (%). DPP4=dipeptidyl peptidase, GLP-1=glucagon-like peptide-1, SGLT2=sodium/glucose cotransporter.

Table S4. Nutrition support.

	Closed-loop (n=21)	Control (n=22)
Number of participants receiving parenteral nutrition	6 (28·6)	7 (31·8)
Number of participants receiving enteral nutrition	14 (66·7)	13 (59·1)
Number of participants receiving parenteral and enteral nutrition	1 (4·8)	2 (9·1)
Mean daily carbohydrate (g/24h)	207·4 (56·7)	209·6 (76·4)
Mean daily carbohydrate received as parenteral nutrition (g/24h)	130·8 (54·9)	144·9 (72·5)
Mean daily carbohydrate received as enteral nutrition (g/24h)	175·8 (64·6)	181·9 (74·7)
Mean daily carbohydrate received as oral intake (g/24h)	61·8 (43·0)	47·1 (37·1)

Data are n (%) or mean (SD)
3 participants received both enteral and parenteral nutrition during the study period

Table S5. Glucose control by site.

	**Cambridge (n=21)**	**Bern (n=22)**
Percentage of time spent at glucose level		
5·6 to 10·0mmol/l [*]	52·7 (26·7)	51·5 (27·9)
> 10·0mmol/l	38·1 (28·7)	39·7 (29·7)
> 20·0mmol/l	4·5 (12·6)	2·2 (7·6)
< 5·6mmol/l	9·2 (9·8)	8·9 (7·3)
< 3·9mmol/l	0·5 (0·0 – 2·3)	0·5 (0·0 – 1·7)
< 3·0mmol/l	0·0 (0·0 - 0·6)	0·0 (0·0 – 0·2)
< 2·8mmol/l	0·0 (0·0 - 0·4)	0·0 (0·0 – 0·1)
Mean glucose (mmol/l)	10·2 (3·4)	9·9 (2·6)
Standard deviation of glucose (mmol/l)	2·9 (1·2)	2·8 (1·3)
CV of glucose (%)	28·4 (8·6)	28·0 (8·1)
Between days CV of glucose (%)	17·5 (10·5)	17·3 (9·0)
AUC_{Day} below 3·5mmol/l (mmol/l × minute)	2·4 (0·0 – 59·2)	1·5 (0·0 – 29·0)
AUC_{Day} below 3·0mmol/l (mmol/l × minute)	0·0 (0·0 – 14·4)	0·0 (0·0 – 3·5)
Total daily insulin dose (units)	52·6 (33·7-82·6)	33·5 (27·1-55·8)
Number of events with capillary glucose <3·5mmol/l	8	5
Number [%] of participants with capillary glucose <3·5mmol/l	5 [24]	2 [9]

Data presented as mean (SD), or median (IQR)

AUC_{Day} - area under the curve for a glucose concentration of less than 3·5mmol/l and 3·0mmol/l per 24-h period

Table S6. Nutrition support regimens and glucose control.

	Enteral nutrition (n=27)		Parenteral nutrition (n=13)	
	Closed-loop (n=14)	Control (n=13)	Closed-loop (n=6)	Control (n=7)
Continuous over 24h	0 [0]	4 [31]	6 [100]	7 [100]
Cyclic day	6 [43]	3 [23]	0 [0]	0 [0]
Cyclic day & night	8 [57]	6 [46]	0 [0]	0 [0]
Percentage of time spent at glucose level				
5·6 to 10·0mmol/l *	62·0 (12·5)	37·3 (27·0)	85·6 (6·8)	38·5 (30·7)
> 10·0mmol/l	27·0 (15·1)	55·3 (29·7)	8·3 (6·7)	49·1 (34·3)
> 20·0mmol/l	0·4 (0·8)	6·3 (13·4)	0·0 (0·0)	7·0 (6·7)
< 5·6mmol/l	11·0 (6·8)	7·4 (8·8)	6·1 (3·6)	12·4 (13·8)
< 3·9mmol/l	0·7 (0·3 – 1·8)	0·0 (0·0 – 2·5)	0·1 (0·0 – 0·4)	1·9 (0·0 – 5·2)
< 3·0mmol/l	0·0 (0·0 - 0·3)	0·0 (0·0 – 1·2)	0·0 (0·0 – 0·0)	0·6 (0·0 – 0·8)
< 2·8mmol/l	0·0 (0·0 - 0·2)	0·0 (0·0 – 0·9)	0·0 (0·0 – 0·0)	0·3 (0·0 – 0·6)
Mean glucose (mmol/l)	8·8 (1·2)	11·5 (3·5)	7·7 (0·6)	11·0 (4·1)
Standard deviation of glucose (mmol/l)	2·6 (0·8)	3·3 (1·5)	1·6 (0·3)	3·4 (1·3)
CV of glucose (%)	28·9 (6·0)	28·3 (9·6)	21·1 (3·1)	31·6 (11·0)
Between days CV of glucose (%)	15·8 (6·6)	17·4 (11·8)	11·9 (4·8)	25·0 (11·2)
AUC_{Day} below 3·5mmol/l (mmol/l × minute)	3·1 (0·0 – 55·1)	0·0 (0·0 – 76·4)	0·0 (0·0 – 2·1)	41·4 (0·0 – 144·2)
AUC_{Day} below 3·0mmol/l (mmol/l × minute)	0·0 (0·0 – 13·4)	0·0 (0·0 – 46·0)	0·0 (0·0 – 0·0)	12·4 (0·0 – 31·2)
Total daily insulin dose (units)	57·8 (28·4 - 89·3)	37·5 (30·0 – 57·9)	28·8 (24·5 - 68·2)	45·0 (29·0 - 52·6)
Number of events with capillary glucose <3·5mmol/l	4	3	0	6
Number of participants with capillary glucose <3·5mmol/l	2 [14]	1 [8]	0 [0]	4 [57]

Data are n [%], mean (SD), or median (IQR)

3 participants received both parenteral and enteral nutrition and were not included in this analysis

Table S7. Utility assessment.

	Closed-loop (n=21)	Control (n=22)	p value
Duration in study (days)	6·7 (4·0)	6·7 (4·3)	0·99
Time when sensor glucose data available (%)	97·4 (3·8)	91·8 (8·1)	0·007
Time when CL in operation (%)	96·5 (3·5)	-	-
Time when CL in operation while sensor data available (%)	99·2 (4·1)	-	-

Data are mean (SD)

Figure S1. Example of the automated fully closed-loop insulin delivery prototype (FlorenceD2W-T2) used in the study (photo obtained with consent).

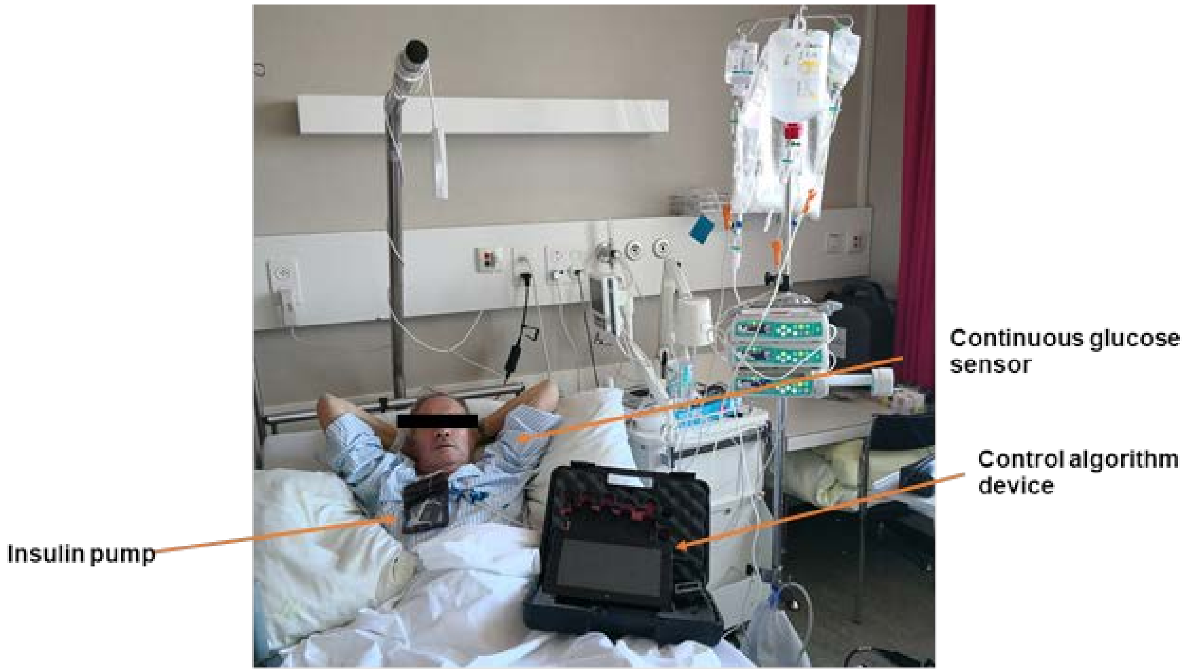

Figure S2. Participant stopping points.

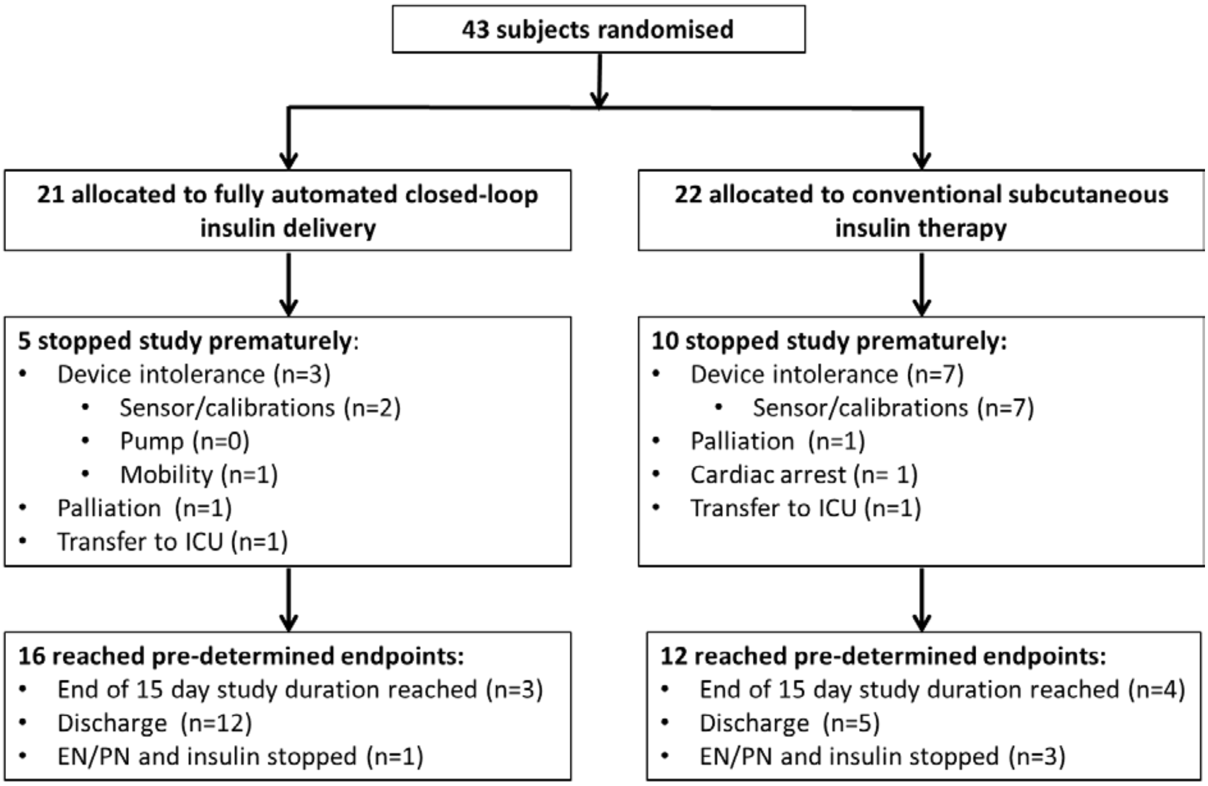

Figure S3. Participants' responses to the feedback questionnaire about closed-loop insulin delivery.

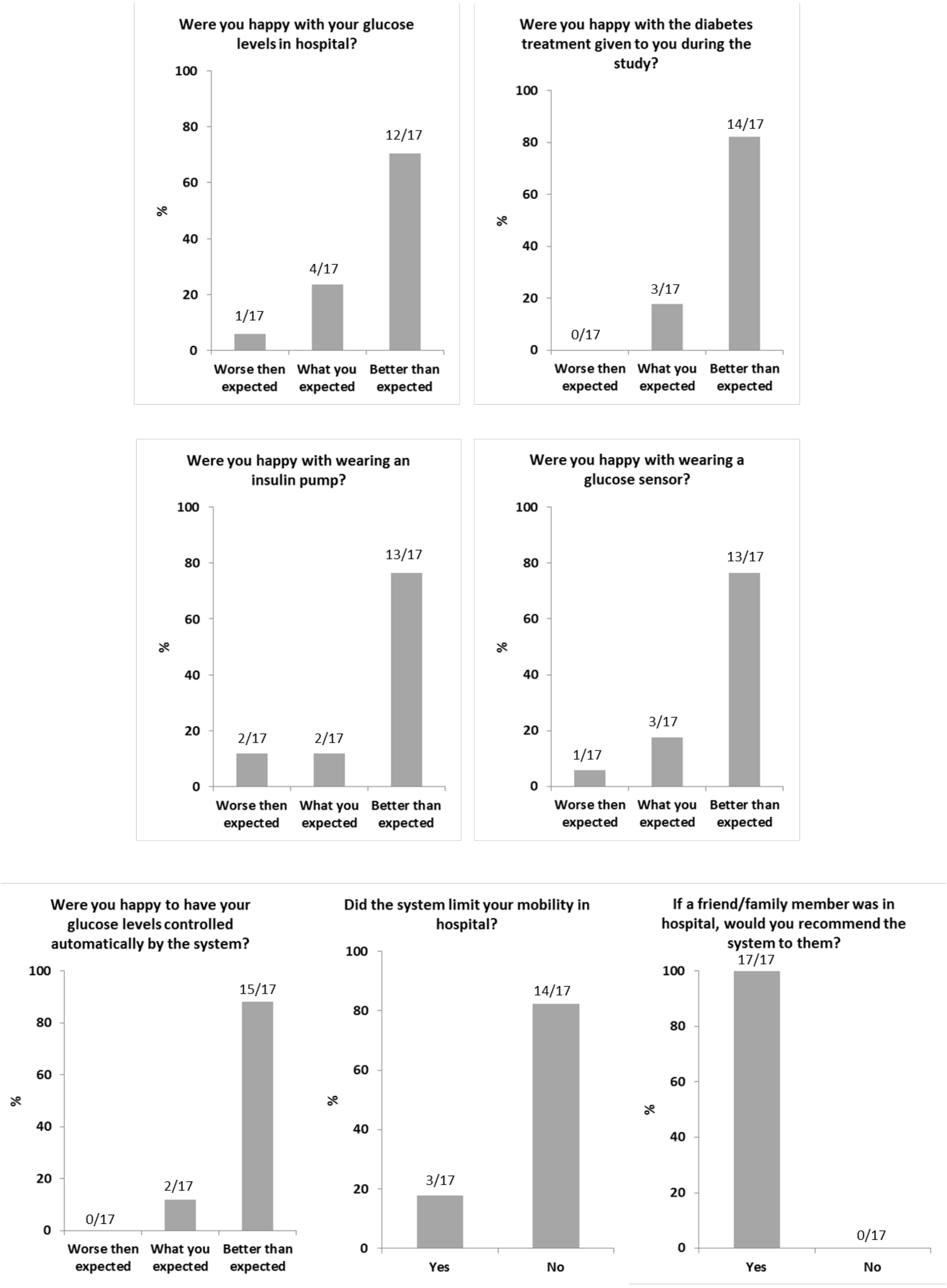

Clinical Study Protocol

**Study Title:**	**A randomised study to assess the efficacy and safety of automated closed-loop glucose control in insulin treated type 2 diabetes (phase 1), inpatient hyperglycaemia requiring subcutaneous insulin therapy (phase 2 and phase 3) and to evaluate use of closed-loop applying faster insulin aspart versus standard insulin aspart (phase 4)**
**Short Title:**	**Achieving Normal Glucose in Hospital Settings (Angie02)**

Protocol Version: **7.2** **12 April 2018**

**Chief Investigator**	Dr Roman Hovorka<br>Institute of Metabolic Science<br>University of Cambridge<br>Box 289, Level 4<br>Addenbrooke's Hospital<br>Hills Road, Cambridge<br>CB2 0QQ<br>UK<br>Phone: +44 1223 762 862<br>Fax: +44 1223 336 996<br>rh347@cam.ac.uk

This protocol has been written in accordance with current ISO 14155 standards

**Principal Clinical Investigators**	Dr Anthony Coll<br>Institute of Metabolic Science<br>Box 289, Level 4<br>Addenbrooke's Hospital<br>Hills Road<br>Cambridge CB2 0QQ<br>UK<br>Phone: +44 1223 769 041<br>apc36@cam.ac.uk<br><br>Professor Christoph Stettler<br>Department of Diabetes, Endocrinolgy, Clinical Nutrition and Metabolism<br>Inselspital, Bern University Hospital<br>University of Bern<br>Bern<br>Switzerland<br>Phone: +41 (0)31 632 40 70<br>Christoph.stettler@insel.ch
**Clinical Investigators**	Dr Mark Evans<br>Institute of Metabolic Science<br>Box 289, Level 4<br>Addenbrookes Hospital<br>Hills Road<br>Cambridge<br>CB2 0QQ<br>UK<br>Phone: +44 1223 336 994<br>mle24@cam.ac.uk<br><br>Dr Hood Thabit<br>Institute of Metabolic Science<br>Box 289, Level 4<br>Addenbrooke's Hospital<br>Hills Road<br>Cambridge CB2 0QQ<br>UK<br>Phone: +44 1223 769080<br>ht312@medschl.cam.ac.uk<br><br>Professor Drahomir Aujesky<br>Department of General Internal Medicine<br>Inselspital, Bern University Hospital<br>University of Bern<br>Bern<br>Switzerland<br>Phone: +41 (0)31 632 88 84<br>Fax: +41 (0)31 632 88 85<br>drahomir.aujesky@insel.ch

	Dr Lia Bally<br>Department of Endocrinology, Diabetes, Clinical Nutrition and Metabolism and Department of General Internal Medicine<br>Inselspital, Bern University Hospital<br>University of Bern<br>Bern<br>Switzerland<br>Phone: +41 (0) 79 852 47 08<br>Lia.bally@insel.ch<br><br>Dr Mariam Semmo<br>Department of Nephrology, Inselspital, Bern University Hospital<br>University of Bern<br>Bern<br>Switzerland<br>Phone: +41 (0) 31 632 21 98<br>mariam.semmo@insel.ch<br><br>Dr Thomas Zueger<br>Department of Endocrinology, Diabetes, Clinical Nutrition and Metabolism Inselspital, Bern University Hospital<br>University of Bern<br>Bern<br>Switzerland<br>Phone: +41 (0) (0) 79 852 47 08<br>thomas.zueger@insel.ch
**Other Investigators**	Dr Malgorzata E Wilinska<br>Institute of Metabolic Science<br>University of Cambridge<br>Box 289, Level 4<br>Addenbrooke's Hospital<br>Hills Road, Cambridge<br>CB2 0QQ<br>UK<br>Phone: +44 1223 769 065<br>Fax: +44 1223 336 996<br>mew37@cam.ac.uk
**Study Statistician**	Dr Roman Hovorka<br>Institute of Metabolic Science<br>University of Cambridge<br>Box 289, Level 4<br>Addenbrooke's Hospital<br>Hills Road, Cambridge<br>CB2 0QQ<br>UK<br>Phone: +44 1223 762 862<br>Fax: +44 1223 336 996<br>rh347@cam.ac.uk
**Clinical Laboratories**	Karen Whitehead<br>University Department of Paediatrics<br>Level 8, Box 116

	Addenbrooke’s Hospital<br>Hills Road, Cambridge<br>CB2 0QQ<br>UK<br>Phone: +44 1223 762945<br>Fax: +44 1223 336996<br>kw240@cam.ac.uk<br><br>Alexander Benedikt Leichtle<br>Centre of Laboratory Medicine, Inselspital<br>Bern University Hospital, University of Bern<br>Freiburgstrasse<br>3010 Bern<br>Switzerland<br>Phone: +41 31 632 83 30<br>Alexander.leichtle@insel.ch	
**Study Sponsor (UK)**	Cambridge University Hospitals NHS Foundation Trust, jointly with University of Cambridge	
	Jo Martindale,<br>Research Service Division<br>University of Cambridge<br>16 Mill Lane,<br>Cambridge, CB2 1SB,<br>UK<br>Phone: +44 (0)1223 765191<br>Fax: +44 (0)1223 332988<br>Jo.Martindale@admin.cam.ac.uk	Stephen Kelleher<br>Cambridge University Hospitals<br>NHS Foundation Trust<br>Box 277, Addenbrooke's Hospital<br>Hills Road, Cambridge, CB2 0QQ,<br>UK<br>Phone: +44 (0) 1223 217418<br>Fax: +44 (0) 1223 348494<br>r&denquiries@addenbrookes.nhs.uk
**Representative of the Sponsor and Principal Investigator (Switzerland)**	Bern University Hospital, University of Bern	
	Professor Christopher Stettler<br>Department of Endocrinology, Diabetes, Clinical Nutrition and Metabolism,<br>Inselspital, Bern University Hospital<br>University of Bern<br>Bern<br>Switzerland<br>Phone: +41 (0)31 632 40 70<br>Christoph.stettler@insel.ch	

Data Monitoring and Ethics Committee

DMEC will consist of

- Independent Chair
- Two independent Medical Experts

PROTOCOL SIGNATURE PAGE

The signature below documents the approval of protocol Version 7.2 dated 12 April 2018 and provides the necessary assurances that this study will be conducted according to all stipulations of the protocol, the principles of GCP and the appropriate reporting requirements.

Signature ……………………………………. Date ....
Dr Roman Hovorka, Chief Investigator

PROTOCOL SIGNATURE PAGE

The signature below documents the approval of protocol Version 7.2 dated 12 April 2018 and provides the necessary assurances that this study will be conducted according to all stipulations of the protocol, the principles of GCP and the appropriate reporting requirements.

Signature ……………………………………. Date ....

Professor Christoph Stettler, Principal Clinical Investigator

Table of Contents

1 LIST OF ABBREVIATIONS 11

2 STUDY SYNOPSIS 12

3 STUDY SUMMARY 19

4 BACKGROUND 21

4.1 PRECLINICAL TESTING 22
4.2 PREVIOUS CLINICAL EXPERIENCE 23
4.2.1 Studies of Closed-Loop in Children and Adolescents with Type 1 Diabetes 23
4.2.2 Studies of Closed-Loop in Adults with Type 1 Diabetes 23
4.2.3 Studies of Closed-Loop in Adults with Type 2 Diabetes 24
4.2.4 Day and Night Closed-Loop Control 25
4.2.5 Manual Operational Mode 25
4.2.6 Automated Closed-Loop Mode 26
4.2.7 Closed-Loop Studies in the Home Setting 26
4.3 RATIONALE FOR CURRENT STUDY 27

5 OBJECTIVES 28

5.1 EFFICACY 28
5.2 SAFETY 28

6 STUDY DESIGN 28

6.1 DESCRIPTION 28

7 STUDY SUBJECTS 29

7.1 STUDY POPULATION 29
7.2 INCLUSION CRITERIA 29
7.3 EXCLUSION CRITERIA 29

8 METHODS UNDER INVESTIGATION 30

8.1 NAME AND DESCRIPTION OF THE METHOD OF INVESTIGATION 30
8.2 INTENDED PURPOSE 30
8.3 METHODS OF ADMINISTRATION 30
8.4 DEVICE MODIFICATION 30
8.5 REQUIRED TRAINING 30
8.6 COMPLIANCE WITH STUDY INTERVENTIONS 30
8.7 PRECAUTIONS 31
8.8 ACCOUNTABILITY OF THE METHOD UNDER INVESTIGATION 31
8.9 PACKAGING, LABELLING, STORAGE AND SUPPLY 31

9 STUDY SCHEDULE 32

9.1 OVERVIEW 32
9.2 RECRUITMENT 33
9.3 RANDOMISATION 34
9.4 CLOSED-LOOP INSULIN DELIVERY VERSUS CONVENTIONAL TREATMENT 34
9.4.1 Fully Closed-loop Insulin Delivery 34
9.4.2 Conventional Therapy 35
9.5 PHASE 1 SEQUENCE OF PROCEDURES: FULLY CLOSED-LOOP INSULIN DELIVERY 35
9.5.1 Day 1 35
9.5.2 Day 2 36
9.5.3 Day 3 36
9.5.4 Day 4 36
9.6 PHASE 1 SEQUENCE OF PROCEDURES: CONVENTIONAL THERAPY 37

9.6.1 Day 1 ... *37*
9.6.2 Day 2 and Day 3 ... *37*
9.6.3 Day 4 ... *38*
9.7 PHASE 2 AND PHASE 3 SEQUENCE OF PROCEDURES: FULLY CLOSED-LOOP INSULIN DELIVERY ... 38
9.7.1 Day 1 ... *38*
9.7.2 Day 2 until end study ... *39*
9.7.3 End of Study ... *39*
9.7.4 Temporary discontinuation of closed-loop insulin delivery ... *40*
9.8 PHASE 2 AND PHASE 3 SEQUENCE OF PROCEDURES: CONVENTIONAL THERAPY ... 40
9.8.1 Day 1 ... *40*
9.8.2 Day 2 until end of study ... *40*
9.8.3 End of Study ... *41*
9.9 PHASE 4: SEQUENCE OF PROCEDURES ... 46
9.9.1 Visit 1 and Visit 2 ... *46*
9.9.2 Blood Sampling ... *47*
9.9.3 End of Study ... *47*
9.9.4 Subject Reimbursement ... *47*
9.10 END OF STUDY PROCEDURES ... 47
9.11 EXPERIMENT STOPPING RULES ... 48
9.11.1 Termination of Subject's Participation in Study ... *48*
9.11.2 Withdrawal of Study Subject Data from Final Analysis ... *48*

10 ENDPOINTS ... 49

10.1 EFFICACY ENDPOINTS ... 49
10.1.1 Primary efficacy endpoints ... *49*
10.1.2 Secondary efficacy endpoints ... *49*
10.1.3 Other outcomes of interest ... *49*
10.2 SAFETY EVALUATION ... 50

11 SAFETY EVALUATION AND REPORTING ... 51

11.1 DEFINITIONS ... 51
11.1.1 Reportable Adverse Event ... *51*
11.1.2 Adverse Event ... *51*
11.1.3 Adverse Device Effect ... *51*
11.1.4 Device Deficiency ... *51*
11.1.5 Serious Adverse Event ... *51*
11.1.6 Serious Adverse Device Effect ... *52*
11.1.7 Unanticipated and Anticipated Serious Adverse Device Effect ... *52*
11.1.8 Adverse Event Intensity ... *52*
11.1.9 Adverse Event Causality ... *53*
11.2 MONITORING PERIOD OF ADVERSE EVENTS ... 54
11.3 MONITORING PERIOD OF SERIOUS ADVERSE EVENTS ... 54
11.4 RECORDING OF ADVERSE EVENTS/DEVICE DEFICIENCIES ... 54
11.5 SEVERE HYPOGLYCAEMIA ... 54
11.6 REPORTING PROCEDURES ... 55
11.7 ANTICIPATED ADVERSE EVENTS/DEVICE EFFECTS ... 57
11.7.1 Hypoglycaemia and Hyperglycaemia ... *57*
11.7.2 Blood test ... *57*
11.7.3 Cannula Insertion (phase 4 only) ... *57*
11.7.4 Insulin Pump Therapy ... *58*
11.7.5 Continuous Glucose Monitoring ... *58*
11.7.6 Questionnaires ... *58*
11.8 BENEFITS ... 58

12 METHODS AND ASSESSMENTS ... 59

12.1 STUDY PROCEDURES ... 59

12.1.1 Height and Weight ... 59
12.1.2 Point of Care Capillary Glucose Measurements (Phase 1-3) ... 59
12.1.3 Plasma Glucose (Phase 4 only) ... 59
12.1.4 Plasma Insulin (Phase 4 only) ... 59
12.2 Assessment for Safety ... 59
12.3 Assessment for Efficacy ... 61
12.4 Questionnaire ... 61
13 DATA ANALYSIS ... 61
13.1 Derived Indices ... 61
13.2 Evaluative Periods ... 62
13.3 Sample Size and Power Calculations ... 62
13.4 Interim Analysis ... 62
13.5 Statistical Analysis ... 62
14 STUDY MATERIALS ... 63
14.1 Study Products ... 63
14.1.1 Insulin ... 63
14.1.2 Insulin Pump ... 63
14.1.3 Continuous Subcutaneous Glucose Monitor ... 63
14.1.4 CGM Receiver Device ... 63
14.1.5 Control Algorithm Device (CAD) ... 64
15 CASE REPORT FORMS ... 65
16 DATA MANAGEMENT ... 65
16.1 Study Monitoring ... 65
16.2 Data Management ... 65
17 STUDY MANAGEMENT ... 68
17.1 Study Registration ... 68
17.2 Categorisation of Study ... 68
17.3 Study Steering Committee ... 68
18 ETHICS ... 68
18.1 Ethics Committee ... 68
18.2 Declaration of Interest ... 69
18.3 Informed Consent of Study Subjects ... 69
18.4 Protocol Amendments ... 69
19 TIMETABLE ... 70
20 DEVIATIONS FROM PROTOCOL ... 70
21 RESPONSIBILITIES ... 71
21.1 Principal Investigator ... 71
22 REPORTS AND PUBLICATIONS ... 71
23 RETENTION OF STUDY DOCUMENTATION ... 71
24 INDEMNITY STATEMENTS ... 71
25 INSURANCE ... 71
26 REFERENCES ... 72
27 APPENDICES ... 75
27.1 Appendix 1 ... 75
27.2 Appendix 2 ... 77

27.3 APPENDIX 3 79
27.4 APPENDIX 4 81
27.5 APPENDIX 5 83
27.6 APPENDIX 6 84
27.7 APPENDIX 7 85
27.8 APPENDIX 8 86
27.9 APPENDIX 9 87
27.10 APPENDIX 10 88
27.11 APPENDIX 11 89
27.12 APPENDIX 12 90
27.13 APPENDIX 13 91
27.14 APPENDIX 14 93
27.15 APPENDIX 15 94
27.16 APPENDIX 16 95

1 List of Abbreviations

ADE	Adverse Device/Method Effect
AE	Adverse Event
AP	Artificial Pancreas
AR	Adverse Reaction
AUC	Area Under the Curve
CE	Conformité Européenne (CE-mark)
CGM	Continuous Glucose Monitoring
CL	Closed-loop
ClinO	Ordinance of Clinical Trials in Human Research
CSII	Continuous subcutaneous insulin infusion
DMEC	Data Monitoring and Ethics Committee
Fiasp	Faster Insulin Aspart
GCP	Good Clinical Practice
HRA	Human Research Act
MHRA	Medicine and Healthcare products Regulatory Agency
i.v.	Intravenous
MPC	Model-Predictive-Control
R & D	Research and Development
REC	Research ethics committee
RF	Radio Frequency
s.c.	Subcutaneous
SADE	Serious Adverse Device/Method Effect
SAE	Serious Adverse Event
SAR	Serious Adverse Reaction
SUSAR	Suspected Unexpected Serious Adverse Reaction
T1D	Type 1 Diabetes Mellitus
T2D	Type 2 Diabetes Mellitus

2 Study Synopsis

**Title of clinical trial**	A randomised study to assess the efficacy and safety of closed-loop glucose control in comparison with conventional treatment in hospitalised insulin treated T2D patients (phase 1) and inpatient hyperglycaemia requiring subcutaneous insulin therapy (phase 2 and phase 3) and to evaluate use of closed-loop applying faster insulin aspart versus standard insulin aspart (phase 4)
**Short Title**	Achieving Normal Glucose In Hospital Settings (Angie02)
**Sponsor name**	**UK** - University of Cambridge & Cambridge University Hospitals NHS Foundation Trust
**Representative of the Sponsor in Switzerland**	Professor Christoph Stettler<br>Bern University Hospital,<br>University of Bern, Bern,<br>Switzerland
**Trial registration**	**NCT01774565**
**Medical condition or disease under investigation**	Type 2 diabetes mellitus<br>Stress hyperglycaemia
**Systems/Devices**	Closed-loop insulin delivery system:<br>1. Real-time continuous glucose monitoring system (Freestyle Navigator, Abbott Diabetes Care or similar)<br>2. DANA Diabecare R® (SOOIL) or similar CE-marked insulin pump<br>3. Control algorithm device (CAD)<br><br>Phase 1-3<br>Point-of-care capillary glucose meter for reference glucose (NovaStat Strip glucose meter or similar)<br><br>Phase 4<br>Bedside glucose analyser to test plasma glucose (Biosen, EKF Diagnostics for life)

**Study objectives**	Phase 1<br>The study objective is to compare conventional insulin therapy with closed-loop glucose control combined with once daily basal insulin injection over 72 hours in achieving target glucose levels in hospitalised insulin treated T2D subjects.<br>1. EFFICACY: The objective is to assess the efficacy of closed-loop glucose control combined with once daily basal insulin injection in maintaining plasma glucose levels within the target range (5.6 - 10 mmol/l) as compared with conventional therapy.<br>2. SAFETY: The objective is to evaluate the safety of closed-loop glucose control combined with once daily basal insulin injection in terms of episodes of hypoglycaemia and other adverse events.<br>Phase 2<br>The study objective is to compare conventional insulin therapy with closed-loop glucose control up to maximum 15 days in inpatient hyperglycaemia requiring subcutaneous insulin therapy<br>1. EFFICACY: The objective is to assess the efficacy of closed-loop glucose control in maintaining plasma glucose levels within the target range (5.6 - 10 mmol/l) as compared with conventional therapy.<br>2. SAFETY: The objective is to evaluate the safety of closed-loop glucose control in terms of episodes of hypoglycaemia and other adverse events.<br>Phase 3<br>The study objective is to compare conventional insulin therapy with closed-loop glucose control applying faster insulin aspart up to maximum 15 days in insulin-treated inpatients receiving parenteral and/or enteral nutrition.<br>1. EFFICACY: The objective is to assess the efficacy of closed-loop glucose control in maintaining plasma glucose levels within the target range (5.6 - 10 mmol/l) as compared with conventional therapy.<br>2. SAFETY: The objective is to evaluate the safety of closed-loop glucose control in terms of

	episodes of hypoglycaemia and other adverse events.<br><br>Phase 4<br><br>The study objective is to compare automated closed-loop control using faster acting insulin aspart with closed-loop control using standard insulin aspart.<br><br>1. EFFICACY: The objective is to assess the efficacy of closed-loop glucose control in maintaining plasma glucose levels within the target range (5.6 - 10 mmol/l)<br><br>2. SAFETY: The objective is to evaluate the safety of closed-loop glucose control in terms of episodes of hypoglycaemia and other adverse events.
**Study Design**	Phase 1-3<br>An open-label, single-centre (phase1)/multicentre (phase 2 and phase 3), randomised, parallel design study contrasting closed-loop glucose control combined with (phase 1, or without for phase 2 and phase 3) once daily basal insulin injection and conventional insulin therapy.<br><br>Phase 4<br>A double-blind, single-centre, randomised, two-period crossover study, contrasting automated closed-loop control using faster-acting insulin aspart with closed-loop control using standard insulin aspart.
**Study centres**	UK<br>1. Addenbrooke's Hospital, Cambridge<br><br>Switzerland<br>1. Bern University Hospital, Bern
**Study Efficacy Endpoints**	The primary outcome is the proportion of time with glucose levels within target range (5.6 - 10.0 mmol/l) during the study period according to CGM values (phase 1-3) or plasma glucose values (phase 4).<br><br>Secondary outcomes include (all evaluated over the study period)<br>1. Proportion of time with glucose levels below 5.6 mmol/l and above 10.0 mmol/l according to CGM and reference glucose values.

	2. Proportion of time with glucose levels below 3.5 mmol/l according to CGM and reference glucose values.<br>3. Average glucose levels, as recorded by CGM and reference glucose values.<br>4. Standard deviation of glucose levels, as recorded by CGM and reference glucose values.<br>5. AUC below 3.5 mmol/l as recorded by CGM.<br>6. Total daily insulin dose (U/24h) (phase 1-3) or insulin delivered between 07:00 and 17:00<br><br>Phase 4 only<br>7. 2-hour postprandial incremental plasma glucose<br>8. Peak plasma/sensor glucose<br>9. Mean of plasma insulin<br>10. Tmax of plasma insulin<br>11. Cmax of plasma insulin<br>12. Total and endogenous insulin exposure within 1 hour postprandial period (iAUC)
**Safety Evaluation**	Assessment of frequency of hypoglycaemic episodes and other adverse events.
**Other outcomes of interest**	1. Daily carbohydrate intake (g/24h)<br>2. Staff workload burden assessment using questionnaires (Switzerland only)<br>3. Metabolic and inflammatory profiling on serum samples (Switzerland only)<br>4. Insulin pharmacokinetics for both faster-acting insulin aspart and standard insulin aspart.
**Participant Evaluation**	Evaluation of participants' experience and feedback during the study.
**Sample Size**	Phase 1: Up to 50 adults will be recruited to allow for 40 subjects available for assessment (20 subjects in each group).<br>Phase 2: Up to 150 adults will be recruited, to allow 120 subjects with at least 48-hours data available for assessment (60 subjects in each group).<br>Phase 3: Up to 50 adults will be recruited to allow 40 subjects with at least 48-hours data available for assessment (20 subjects in each group)<br>Phase 4: Up to 15 adults will be recruited to allow for 10 subjects available for assessment

Summary of eligibility criteria

Key inclusion criteria:

Phase 1

1. Aged 18 years or older
2. T2D for at least 1 year as defined by WHO
3. Treatment with subcutaneous insulin alone or in combination with oral glucose-lowering medication(s)

Phase 2

1. Aged 18 years or older
2. Deemed by clinical team to require subcutaneous insulin therapy for inpatient glycaemic control

Phase 3

1. Aged 18 years or older
2. Deemed by clinical team to require subcutaneous insulin therapy for inpatient glycaemic control
3. Receiving parenteral and/or enteral nutrition

Phase 4

1. Aged 18 years or older
2. Type 2 diabetes for at least 1 year as defined by WHO
3. Treatment with insulin (basal-bolus) for at least 3 months
4. HbA1c<11.0%

Key exclusion criteria:

1. Autoimmune type 1 diabetes
2. Known or suspected allergy against insulin
3. Known proliferative retinopathy
4. Current or planned pregnancy or breast feeding
5. Unstable or end-stage cardiac and renal disease (phase 1 only)
6. Planned surgery during study period (phase 1 only)
7. Current inpatient in intensive care unit
8. Any physical or psychological disease or medication(s) likely to interfere with the conduct of the study and interpretation of the

	study results, as judged by the study clinician<br>9. Likely discharge earlier than 72 hours<br>10. Withdrawal of informed consent (phase 3)
**Maximum duration of study for a subject**	Phase 1: 72 hours<br>Phase 2 and phase 3: up to maximum 15 days<br>Phase 4: two 24-hour visits separated by a washout period of 1-4 weeks
**Recruitment**	The subjects will be recruited following admission to hospital under the treating medical team (phase 1-4) or from the diabetes outpatient clinic (phase 4)
**Study Schedule**	<u>Phase 2</u><br>August 2016 first participant<br>August 2018 last participant (anticipated)<br><u>Phase 3</u><br>January 2018 first participant<br>December 2018 last participant (anticipated)<br><u>Phase 4</u><br>April 2018 first participant<br>December 2018 last participant (anticipated)
**Consent**	Participants will be asked to provide written informed consent.<br>Phase 3 only: Written informed consent will be obtained from the patient or from the next of kin if the patient is incapacitated. If the patient lacks capacity and no next of kin is available, written informed consent will be obtained from a senior member of the clinical team responsible for the patient, who is not a member of the study team. Should the subject/next of kin express subsequent disagreement with study enrolment, the subject will be withdrawn from the study and all his/her data destroyed.
**Baseline Assessment**	A brief medical (including diabetes) history will be obtained. A blood sample for HbA1c (and C-peptide, Switzerland only) will be taken at baseline.
**Randomisation**	<u>Phase 1-3</u><br>Eligible subjects will be randomised to one of two groups stated below:<br><br>Group 1: Conventional insulin therapy using masked CGM<br>Group 2: Fully automated closed-loop insulin delivery

	Phase 4<br>Eligible subjects will be randomised to receive closed-loop control with<br>Group 1: faster-acting insulin aspart, followed by standard insulin aspart<br>Group 2: standard insulin aspart, followed by faster-acting insulin aspart
**Investigational Methods for Automated Closed-loop Glucose Control**	Participants will be admitted under the treating medical team as per routine clinical practice. Informed consent will be obtained by member(s) of the research team.<br><br>A subcutaneous CGM will be inserted by a member of the research team and will be calibrated accordingly. A continuous subcutaneous insulin infusion (CSII) pump will also be inserted by the research team. The closed-loop system will then be started once the CGM is calibrated. CGM's low glucose alarm will be activated.<br><br>Once the system operation is initiated, insulin will be adjusted automatically by the closed-loop insulin delivery system every 10 to 12 minutes. Reference blood samples for glucose measurements will be taken using the NovaStat Strip® or similar CE-marked glucose meter, before each meal and before bedtime (phase 1-3). In the fully closed-loop insulin delivery arm, insulin delivery will be continuously adjusted automatically by the control algorithm according to sensor glucose values. A once daily injection of subcutaneous basal insulin injection will be administered to each subject, at 20% of their usual total daily insulin dose (phase 1 only). In phase 2, subcutaneous basal insulin injection at 20% of their usual total daily insulin dose will only be administered if closed-loop system needs to be disconnected for more than 1 hour due to inpatient investigation such as magnetic resonance imaging (MRI).<br><br>Subjects will be allowed to continue with their usual oral hypoglycaemic therapy and/or non-insulin injectable therapy, with the exception of sulphonylurea agents, which will be discontinued during the study.

	The study will not interfere with nor specify any nutritional intake or activity of the patient in the ward. All other inpatient activities will be decided by the treating clinicians, as part of their routine clinical care. The study will conclude after 72 hours of closed-loop glucose control in phase 1, up to maximum 15 days for phase 2 and phase 3 and after the second 24-hour visit in phase 4. At the end of the study the patient will be transferred to standard therapy according to existing hospital guidelines.
**Standard Therapy for Glucose Control**	Subjects randomised to the Control arm (Standard therapy) will have a subcutaneous CGM inserted by a member of the research team which will be calibrated accordingly. The investigator and subject will be blinded to the CGM measurements.<br>Subject's glucose control will be managed as per standard clinical care.
**End of study evaluation**	Subjects will be invited to complete a questionnaire evaluating their experience and views of the diabetes treatment received.
**Procedures for safety monitoring during trial**	Standard operating procedures for monitoring and reporting of all adverse events will be in place, including serious adverse events (SAE) and specific adverse events (AE) such as severe hypoglycaemia.
**Criteria for withdrawal of patients on safety grounds**	A subject may terminate participation in the study at any time without necessarily giving a reason and without any personal disadvantage. An investigator can stop the participation of a subject after consideration of the benefit/risk ratio. Possible reasons are:<br>1. Serious adverse events<br>2. Decision by the investigator, or the sponsor, that termination is in the subject's best medical interest<br>3. Pregnancy<br>4. Allergic reaction to insulin

3 Study Summary

The main study objective is to compare conventional insulin therapy with automated closed-loop glucose control in achieving target glucose levels in hospitalised insulin-treated T2D subjects (phase 1) and inpatient hyperglycaemia requiring subcutaneous insulin therapy (phase 2 and phase 3).

In phase 4 extension, the purpose is to compare the efficacy of automated closed-loop glucose control without meal-bolusing applying faster-acting insulin aspart compared to standard insulin aspart.

This is an open-label, two-arm, randomised, parallel design study in hospitalised insulin-treated T2D subjects (phase 1) and inpatient hyperglycaemia requiring subcutaneous insulin therapy (phase 2 and phase 3), during which target glucose levels will be controlled either by closed-loop system combined with (phase 1 only) once daily basal insulin injection or by conventional insulin therapy in random order. In phase 4 extension, efficacy of automated closed-loop control using faster-acting insulin aspart versus standard insulin aspart will be assessed in insulin-treated T2D subjects in a double-blind two-period crossover design study.

In phase 1, up to a total of 50 adults with insulin-treated T2D aged 18 years or older will be recruited, to allow for 40 subjects available for assessment. In phase 2, up to a total of 150 adults requiring subcutaneous insulin therapy aged 18 years or older will be recruited, to allow for 120 subjects with at least 48 hours data available for assessment. In phase 3, up to 50 adults requiring subcutaneous insulin therapy aged 18 years or older will be recruited to allow for 40 subjects with at least 48 hours data available for assessment (20 in each group). Recruitment will take place following admission to hospital by the clinical care team. In phase 4 extension, up to 15 adults with insulin-treated T2D will recruited to allow for 10 subjects with completed study periods.

The primary outcome is the proportion of time with glucose levels between 5.6 and 10.0 mmol/l during the 72-hour study period (phase 1), up to 15-days (phase 2 and phase 3) and during the 10-hour period (phase 4) according to CGM levels (phase 1-3) or plasma glucose levels (phase 4).

4 Background

Hyperglycaemia in hospitalised patients is becoming a common clinical problem due to the increasing prevalence of diabetes mellitus. Hyperglycaemia in this cohort can also occur in patients with previously undiagnosed diabetes, or during acute illness in those with previously normal glucose tolerance. As a result, the prevalence of acute or stress hyperglycaemia in hospitalised patients has been widely reported. This has previously been generally regarded as inconsequential, since hyperglycaemia in this group of patients has been thought to be beneficial during physiologically “stressful” periods, to ensure adequate supply of glucose as substrate to organs that do not require insulin for glucose uptake (i.e. the brain and immune system)(1). A growing body of evidence currently suggests that the degree of hyperglycaemia upon admission and the duration of hyperglycaemia during their illness are associated with adverse outcomes(2, 3). Inpatient hyperglycaemia is now widely recognised as a poor prognostic marker in terms of morbidity and mortality, increased length of stay and cost to the healthcare system(4, 5).

Analysis of data from nine randomised controlled trials and ten observational studies reported that treatment of hyperglycaemia in non-critically ill patients was associated with reduction in the risk of infection (relative risk, 0.41;95% confidence interval, 0.21-0.77)(6). In 2001, the Leuven study demonstrated significant reductions in morbidity and mortality with intensive glucose control (4.4-6.1mmol/l) in surgical critical care patients(7). In contrast, no benefit of tight glycaemic control was seen in the recent NICE SUGAR multicentre trial, with the added observations of higher 90-day mortality and an increased frequency of severe hypoglycaemia(8). Various reasons have been cited for these conflicting results. Amongst the reasons is substantial variability in blood glucose sampling methods, as well as differences in insulin infusion protocols and glucose targets between study centres. Optimal glycaemic inpatient glucose targets still remain an intensely debated subject. In critically ill patients, an updated consensus from the American Association of Clinical Endocrinologists (AACE) and American Diabetes Association (ADA) recommended specified targets of fasting or pre-meal blood glucose <7.8mmol/l and random blood glucose <10mmol/l(9). The guidelines in non-critically ill patients however are based on clinical experience alone, as the majority of studies have been carried out in the intensive care setting.

The current management of inpatient hyperglycaemia in non-critical care is still far from ideal, and vary widely between different centres(9, 10). The discordance between clinical evidence and practice is due to a number of factors which could potentially undermine patient care and safety. Of these, hypoglycaemia remains one of the biggest barriers to managing inpatient hyperglycaemia(11). A recent meta-analysis reported that intensive glycaemic control in non-critical care patients is associated with a trend of increased risk of hypoglycaemia(6). It is estimated that each patient with diabetes or hypoglycaemia experiences 2 episodes of hypoglycaemia during their hospitalisation, the majority occurring overnight(12). Poor knowledge of insulin

therapy among healthcare providers and unexpected nutritional interruption coupled with failure to adjust patient's insulin therapy can contribute to the prevalence of inpatient hypoglycaemia(11). In addition, the implementation of intravenous insulin sliding scale and various regimes of subcutaneous insulin to optimise glucose control in most hospitals continue to be an ongoing challenge due to reduced staffing levels and increased workload(11). There is therefore a need to develop and validate a more effective and safer system to manage inpatient hyperglycaemia.

A closed-loop insulin infusion system has previously been tested and reported to be feasible and safe in intensive care patients(13). Its utilisation in non-critical patients in the general medical and surgical wards currently remains understudied. Its use in this cohort however could potentially be of significant practical and clinical value, especially in a busy ward environment. A closed-loop system consists of a subcutaneous glucose sensor which provides real-time glucose value feedback from the patient to a control algorithm. The control algorithm then autonomously determines the rate of insulin infused to maintain euglycaemia, based on the glucose sensor feedback. The Model Predictive Control (MPC) algorithm developed by our group at the University of Cambridge utilises fundamental glucoregulatory processes and predicts future glucose excursion resulting from projected insulin infusion rates(14). The algorithm can also account for the patient's meal intake and the duration of action of the short acting insulin used. This has the distinct advantage over the "reactive" approach of sliding scale insulin protocols, which treats hyperglycaemia after it has already occurred. A recent study evaluating the application of an automated fully closed-loop insulin delivery system in the general ward setting showed efficacy and safety, with improved time spent within target glucose range (5.6 - 10.0mmol/l) without further increase in risk of hypoglycaemia(15).

The MPC algorithm has been studied in intensive care and cardiac surgery patients, and results from these studies to date have been encouraging(14, 16, 17). It is shown to be associated with a significantly higher percentage of time within the blood glucose target range, without increasing the risk of severe hypoglycaemia. The expected role of a closed-loop system using the MPC algorithm in non-critical care patients would therefore be to provide clinicians with an effective and safe method to manage hyperglycaemia in hospital.

4.1 Preclinical Testing

The closed-loop model predictive control (MPC) algorithm has been studied extensively using *in silico* testing utilising a simulator developed by members of the study team(18). The simulations suggested a reduced risk of nocturnal hypoglycaemia and hyperglycaemia with the use of the MPC algorithm(19).

4.2 Previous clinical experience

4.2.1 Studies of Closed-Loop in Children and Adolescents with Type 1 Diabetes

To date, 25 children and adolescents with T1D have been studied on 70 study nights. Closed-loop (CL) glucose control was maintained on 49 of those nights, corresponding to a total of 584 hours. In the first study the feasibility and safety of overnight CL glucose control was compared with conventional insulin pump therapy in 12 children. Overnight closed-loop increased plasma glucose time in target (range 3.9-8.0mmol/l) by 37% and reduced hypoglycaemia risk index 8-fold (Appendix 2). The CL was further evaluated after slowly and rapidly absorbed large evening meals on two separate study nights (APCam02), with results indicating similar efficacious glucose control regardless of the meal composition (Appendix 3). A third study tested the ability of overnight CL to prevent nocturnal hypoglycaemia following afternoon exercise. Overnight CL was not associated with hypoglycaemia and increased the time spent in target glucose, compared with conventional pump therapy (Appendix 4). Secondary analysis of pooled APCam01 and APCam03 data demonstrated increased time for which plasma glucose was in target between 3.9 and 8.0mmol/l (60% on CL vs 40% on CSII, p=0.002), and reduced time below target (2.1% on CL vs 4.1% on CSII, p=0.03) (Appendix 1)(20). There were no plasma glucose values below 3.0mmol/l and no rescue carbohydrates administered during any of the 33 CL nights.

4.2.2 Studies of Closed-Loop in Adults with Type 1 Diabetes

We have completed two randomised overnight CL studies in 24 adults with T1D, testing a similar closed-loop system comprising off the shelf CGM and pump devices and a MPC algorithm. The first study (n=12) assessed the feasibility and efficacy of overnight CL insulin delivery following a moderate-sized (60g carbohydrate) evening meal compared with conventional pump therapy. We demonstrated that overnight CL insulin delivery, compared with usual CSII, significantly increased time in target plasma glucose range (3.9-8 mmol/l) by 24% and reduced glycaemic variability as measured by standard deviation of plasma glucose (Appendix 5)(21). The improvements in glucose control seen on CL were even greater after midnight, when time in target increased by 41%. In the second study we tested the efficacy of overnight CL following a common situation such as consuming a large (100g carbohydrate) evening meal and drinking alcohol (0.75g ethanol/kg body weight of 13% ethanol white wine). We showed that overnight CL insulin delivery, compared with conventional CSII, similarly increased time in target plasma glucose between 3.9 and 8.0mmol/l by 24% and reduced time spent above target by 11%, even following such challenges (Appendix 6)(22). Importantly these improvements during CL were achieved with no increased requirement in the average rate of insulin infusion overnight. Pooled results of both studies demonstrated increased time for which plasma glucose was in target between

3.91 and 8.0 mmol/l (76% on CL vs. 50% on CSII, p<0.001), and reduced time below target (2.8% on CL vs. 6.7% on CSII, p=0.04) (Appendix 7)(23). These studies build on earlier CL studies carried out in children and adolescents using the same algorithm, providing further evidence of the safety and efficacy of overnight CL across a range of age groups.

4.2.3 Studies of Closed-Loop in Adults with Type 2 Diabetes

We have recently completed a randomised cross-over 24-hour closed-loop insulin delivery in twelve subjects with type 2 diabetes on oral hypoglycemic agents utilising Model Predictive Algorithm modified for type 2 diabetes. Interim data of closed-loop visits (Figure 1) utilising FreeStyle Navigator CGM and Animas pump delivering Lispro insulin demonstrates safety of closed-loop insulin delivery in type 2 diabetes.

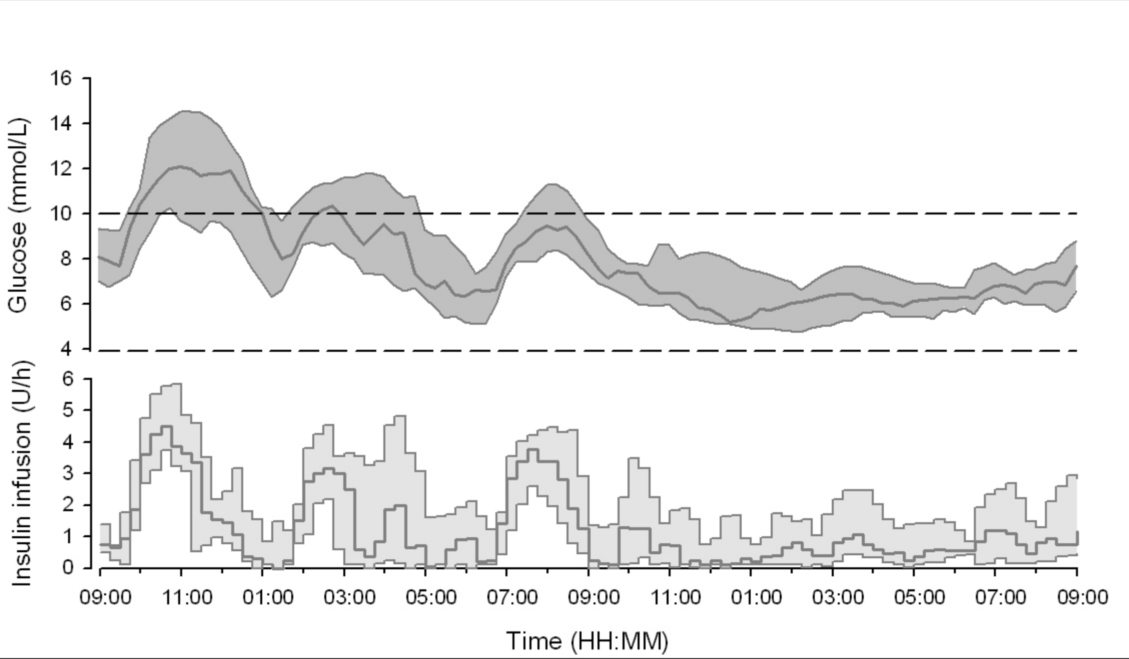

Figure 1. Illustrates the glucose level and insulin delivery profile observed during a 24-hour closed-loop study in a cohort of insulin-naive subject with type 2 diabetes on oral hypoglycaemic agents (oral agents were stopped during the study). The top panel shows the continuous subcutaneous glucose trace (median, IQR). The dashed lines indicate the target glucose range. Insulin infusion rates (median, IQR) during closed-loop delivery are shown in the bottom panel. The control algorithm is unaware of three main meals consumed at 09:00, 13:00 and 19:00.

In phase1, automated fully closed-loop insulin delivery was assessed in inpatients with type 2 diabetes hospitalised in the general wards (15). Forty participants were randomised to either automated fully closed-loop insulin delivery or usual insulin therapy. Results showed that closed-loop significantly increased time spent within target glucose range (5.6-10.0mmol/l, Figure 2), without any increase in the risk of hypoglycaemia.

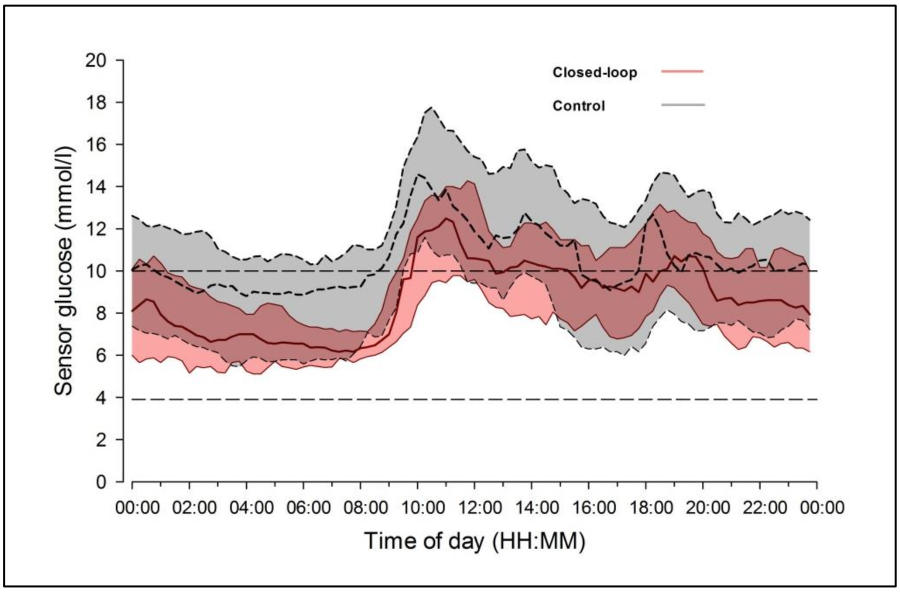

Figure 2. Median (interquartile range) of sensor glucose during closed-loop (the solid red line and the red shaded area) and control interventions (the dashed black line and the grey shaded area) from midnight to midnight. The lower and upper limits of the glucose target range 5.6 to 10.0 mmol/l are denoted by the horizontal dashed lines.

4.2.4 Day and Night Closed-Loop Control

The next goal was to assess the safety and efficacy of closed-loop insulin delivery over a prolonged period including the daytime. Twelve adolescents with type 1 diabetes were evaluated over a 36 hour period, comparing the closed-loop system with their usual insulin pump therapy on two occasions. During the stay at the clinical research facility, participants engaged in normal daily activities, consumed meals and snacks, and undertook moderate-intensity exercise on a stationary bicycle. Results showed time spent with plasma glucose levels within the target range of 3.9 to 10mmol/l was 82%, a 27% increase as compared to conventional pump treatment (Appendix 8)(24). A similar study evaluating 24 hours of closed-loop during daily activities and exercise is currently in progress in women with T1D in pregnancy (Appendix 9)(25).

4.2.5 Manual Operational Mode

The aim of our previous studies was to test the performance and, specifically, the safety and efficacy of the MPC-based glucose control algorithm overnight in children and adults with T1D under conditions of exercise, small versus large meals, and alcohol. In all these studies the closed-loop system involved manual inputs of sensor glucose values into the computer-based algorithm, and manual adjustments of the insulin pump following algorithm generated insulin infusion rate advice.

4.2.6 Automated Closed-Loop Mode

The next step is progression from a manual operational mode to a totally automated closed-loop insulin delivery system. We have recently evaluated such a system in 8 young children, demonstrating feasibility and similar efficacy with initiation of CL at 18:00 versus 21:00 (Appendix 10)(26). On both study nights, blood glucose levels were controlled by an automated closed-loop system which involved a computer-based control algorithm communicating with a CGM device in order to acquire real-time glucose concentration values at one minute intervals. The advised insulin infusion rate was then sent to the insulin pump via wireless link and the pump infusion rate adjusted automatically. This study was developed as part of an MHRA clinical investigation plan (**Ref:** CI/2008/0036).

A further 8 adolescent subjects with T1D will be studied on a similar automated CL system, known as Florence, on the clinical research facility, prior to testing the system in the home setting. Florence is a purpose-built system, including a hand-held computer containing the algorithm and communicating with a CGM device and insulin pump.

4.2.7 Closed-Loop Studies in the Home Setting

Following testing of the Florence system in the clinical research facility, the first home study will be carried out in 16 adolescents with T1D evaluating safety and efficacy in the home setting, with subsequent testing in 24 adults with T1D in a multi-centre trial (REC Ref: 10/H0304/87 and 12/EE/0034).

4.3 Rationale for Current Study

Studies from our group have assessed the safety and efficacy of closed-loop system delivery in T1D in a controlled research setting, with the ultimate goal being the use of closed-loop systems at home. Closed-loop use in hospitalised patients to date has only been evaluated in the intensive care setting, and has demonstrated significant efficacy and safety in achieving target glucose range when compared to standard treatment protocol(13, 16, 27). Despite studies in non-critical care cohorts reporting an adverse relationship between inpatient hyperglycaemia and clinical outcome, glycaemic management of these patients remains suboptimal(5).

Glycaemic targets for inpatients remains a matter of intense debate. Results from inpatient studies have reported that tight glycaemic control is associated with a nearly 6-fold increased risk of hypoglycaemia(28). There is now evidence to suggest that this may lead to higher inpatient mortality, and that a more conservative glucose target should be followed(29). As a result, recent consensus from the American Diabetes Association and American Association of Clinical Endocrinologists have recommended, in non-critical care patients, a premeal glucose target of less than 7.9 mmol/l and a random or postmeal glucose target of less than 10 mmol/l to lessen the risk of hypoglycaemia(30). However, long-term data and outcomes from these targets are currently still unavailable.

Health care providers need to deliver glycaemic control in a safe and efficacious manner. The use of subcutaneous insulin sliding scale is discouraged due to the increased risk of hypoglycaemia from insulin stacking(31). As a result, insulin therapy in hospital is currently being delivered either as intravenous insulin infusion titrated according to frequently sampled blood glucose, or a combination of subcutaneous long-acting basal insulin with rapid acting insulin at mealtimes, titrated according to finger prick glucose testing carried out 4-5 times per day. This has a significant impact on time and staffing levels in the general ward, which are currently under considerable strain(11). The use of real-time continuous glucose monitors (CGM) has several advantages over intermittent blood glucose testing, but still requires interpretation with subsequent adjustment of insulin regimens by clinical staff. An algorithm driven automated closed-loop system enables automated subcutaneous delivery of insulin in response to real-time glucose sensor readings. Studies from our group at the University of Cambridge have reported that closed-loop insulin delivery is likely to be efficacious in hospitalised patients with T1D and insulin-treated T2D (17, 32, 33). Patients with T2D treated by diet or non-insulin glucose-lowering medications alone, an episode of acute illness may result in elevated glucose levels, also known as stress hyperglycaemia, necessitating short term use of insulin to optimise glycaemic control(34). Stress hyperglycaemia may also necessitate short-term adjustment to insulin doses in those already on insulin therapy.

Closed-loop system may be of benefit to such patients in whom the optimal dosing regimen is difficult to establish, and hence may be better facilitated by

an algorithm-initiated and driven insulin therapy. Most importantly, closed-loop may provide a safer method of insulin delivery with the added benefit of continuous monitoring of glucose levels on general hospital wards, thus minimising the likelihood of hyper- and hypoglycaemic events and their known associated worse outcomes.

5 Objectives

5.1 Efficacy

The objective is to assess the efficacy of 72-hour automated closed-loop (CL) glucose control combined with once daily basal insulin injection (phase 1) and up to 15 days automated CL (phase 2 and phase 3) in the general ward setting in maintaining CGM glucose levels within the target range (5.6 to 10.0 mmol/l) compared to conventional glucose control in hospitalised insulin-treated T2D subjects (phase 1) and inpatient hyperglycaemia requiring subcutaneous insulin therapy (phase 2 and phase 3). In phase 4 extension, efficacy of closed-loop glucose control in insulin-treated T2D subjects using faster-acting insulin aspart versus standard insulin aspart will be assessed.

5.2 Safety

The objective is to determine the impact of 72-hour automated closed-loop glucose control combined with once daily basal insulin injection (phase 1) and up to 15 days automated closed-loop (phase 2 and phase 3) in the general wards setting and use of automated closed-loop glucose control using faster-acting insulin aspart versus standard insulin aspart during two 24-hour visits in terms of:

1) incidence of severe hypoglycaemia;
2) occurrence of other adverse events, including severe hyperglycaemia.

6 Study Design

6.1 Description

An open-label, single-centre, randomised, two-arm, parallel design study in hospitalised insulin-treated T2D adults (phase 1) and an open-label, multi-centre, randomised, two-arm, parallel design study in inpatient hyperglycaemia requiring subcutaneous insulin therapy (phase 2 and phase 3), assessing the efficacy and safety of automated closed-loop glucose control system combined with once daily basal insulin injection (phase 1) and automated closed-loop glucose control system only (phase 2 and phase 3) in the general wards setting, as compared to conventional glucose control alone. Phase 4 evaluates the efficacy of automated closed-loop control using faster-acting insulin aspart versus standard insulin aspart in insulin-treated T2D subjects in an inpatient clinical research facility setting.

7 Study Subjects

7.1 Study Population

Up to a total of 50 adults with insulin-treated T2D (phase 1), up to 150 adults requiring subcutaneous insulin therapy (phase 2) and up to 50 adults requiring subcutaneous insulin therapy while receiving parenteral and/or enteral nutrition will be recruited at the general wards following admission to hospital under the treating clinical team, to allow for 40 (phase 1 and phase 3) available for assessment, 120 (phase 2) with at least 48 hours of data. In phase 4 extension, insulin-treated T2D subjects will be recruited from the adult outpatient diabetes clinic and/or during admission to the general wards at the University Hospital Bern.

Potential participants will be identified by their treating clinicians and contacted by the research team. They will then be given the study information leaflets and an invitation to join the study by the research team.

7.2 Inclusion Criteria

Phase 1

1. Aged 18 years or older
2. Type 2 diabetes for at least 1 year as defined by WHO
3. Treatment with insulin therapy with or without oral glucose-lowering medication(s).

Phase 2

1. Aged 18 years or older
2. Deemed by clinical team to require subcutaneous insulin therapy for inpatient hyperglycaemia

Phase 3

1. Aged 18 years or older
2. Deemed by clinical team to require subcutaneous insulin therapy for inpatient hyperglycaemia
3. Receiving parenteral and/or enteral nutrition

Phase 4

1. Aged 18 years or older
2. Type 2 diabetes for at least 1 year as defined by WHO
3. Treatment with insulin (basal-bolus) for at least 3 months
4. HbA1c<11.0%

7.3 Exclusion Criteria

1. Autoimmune type 1 diabetes
2. Known or suspected allergy against insulin
3. Proliferative retinopathy
4. Current or planned pregnancy or breast feeding
5. Unstable or end-stage cardiac and renal disease (phase 1 only)

6. Current inpatient in intensive care unit
7. Planned surgery during study period (phase 1 only)
8. Any physical or psychological disease or medication(s) likely to interfere with the conduct of the study and interpretation of the study results, as judged by the study clinician.
9. Likely discharge earlier than 72 hours (phase 1-3)
10. Withdrawal of informed consent (phase 3)

8 Methods under Investigation

8.1 Name and Description of the Method of Investigation

The investigational treatment is the automated closed-loop insulin delivery system (FlorenceD2W-T2) manufactured by the Cambridge University Hospitals NHS Foundation Trust. Component versions will be identified during regulatory submission to the MHRA (UK) and Swissmedic (Switzerland).

This system has been employed in our closed-loop studies in children, adolescents, adults and pregnant women with type 1 diabetes carried out thus far, demonstrating its safety and efficacy, as detailed in Section 4.2 previously.

8.2 Intended Purpose

The intended purpose of the investigational treatment is closed-loop insulin delivery in inpatients with glucose dysregulation.

8.3 Methods of Administration

The closed-loop system consists of components directly attached to the patient, which are the CGM transmitter and the insulin pump. The components not directly attached to the patient comprise (i) a device which communicates with the CGM transmitter and the insulin pump and (ii) a mobile/laptop computer that contains the closed-loop algorithm, see conceptual design in Figure 3.

8.4 Device Modification

No CE-marked devices were modified for the purposes of the study.

8.5 Required Training

Prior to commencement of the study, the research team nurses/clinicians will be trained to use the closed-loop system and its components.

8.6 Compliance with Study Interventions

Not applicable as no direct patient input is required for study device management and intervention.

8.7 Precautions

During the investigational and conventional treatment with insulin there is a risk of hypoglycaemia and hyperglycaemia. In-hospital testing and Hazard Analysis both documented reduced risk of hypoglycaemia and hyperglycaemia during overnight closed-loop compared to conventional treatment.

8.8 Accountability of the Method under Investigation

The investigator ensures that the closed-loop system is used for the study purposes only.

8.9 Packaging, Labelling, Storage and Supply

Unmodified study devices will be delivered in the original packaging. Non-CE marked study devices will be appropriately labelled. Storage, return or destruction of study devices are according to the agreed standard procedures.

Switzerland only

Serum samples will be coded and stored at -80°C at the central laboratory of the University Hospital of Bern, until analysis is performed.

Figure 3. Conceptual design of the automated closed-loop system

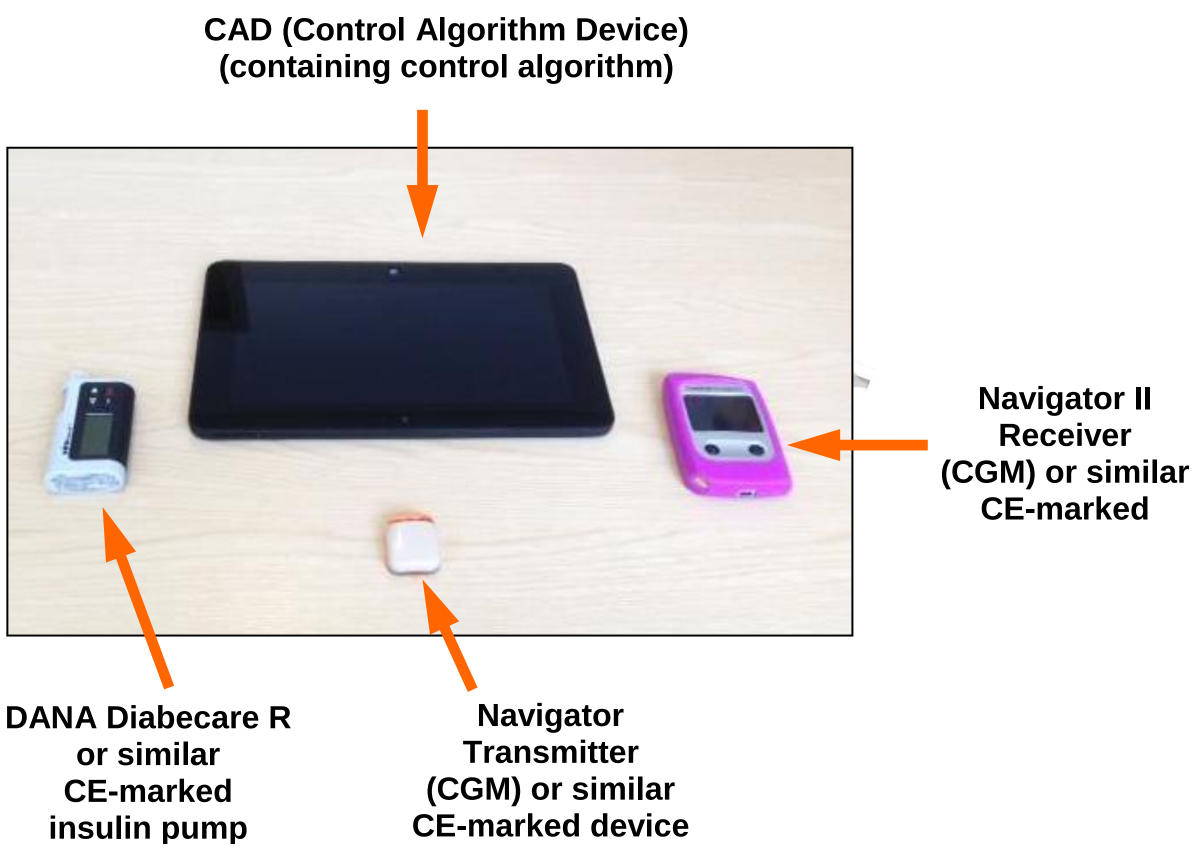

9 Study Schedule

9.1 Overview

Phase 1 - The study will take place at the general ward in Addenbrooke's Hospital. Full details of the study activities are reported in Section 9.5.

Phase 2 and Phase 3 - The study will take place in the general ward at the following sites:

1. Addenbrooke's Hospital, Cambridge, UK
2. Bern University Hospital, Bern, Switzerland

Phase 4 – The study will take place at the nephrology ward of the University Hospital, Bern, Switzerland.

Phase 1

Adults with insulin-treated T2D admitted by the clinical team for inpatient management will be approached. Once informed consent has been obtained, 40 subjects will be randomised to one of two groups:

- Group 1; conventional insulin therapy using masked CGM (Section 9.4.2) for 72 hours
- Group 2; fully automated closed-loop with once daily basal insulin injection (Section 9.4.1) for 72 hours

Group 2 will involve automated variable subcutaneous insulin delivery according to calculations made by the control algorithm and utilising continuous glucose monitoring data.

Phase 2 and Phase 3

Adults admitted by the clinical team for inpatient management who are deemed to require subcutaneous insulin therapy for inpatient hyperglycaemia will be approached. Once informed consent or assent has been obtained, up to 150 subjects (phase 2) and up to 50 subjects on parenteral and or enteral nutrition (phase 3) will be randomised to one of either two groups:

- Group 1; conventional insulin therapy using masked CGM (Section 9.4.2) for up to 15 days
- Group 2; fully automated closed-loop (Section 9.4.1) for up to 15 days

Group 2 will involve automated variable subcutaneous insulin delivery according to calculations made by the control algorithm and utilizing continuous glucose monitoring data. In phase 3, closed-loop control will apply faster-acting insulin aspart (Fiasp).

Phase 4

In order to test the incremental benefit of faster-acting insulin aspart when used in an automated closed-loop glucose-control system (see phase 3), insulin-treated T2D adults from the diabetes outpatient clinic and/or general ward who

are treated with basal-bolus subcutaneous insulin therapy will be recruited. Once informed consent has been obtained, up to 15 subjects will be assigned to the following treatment arms over 24 hours in random order

- Automated closed-loop control using faster-acting insulin aspart (Fiasp)
- Automated closed-loop control using standard insulin aspart

9.2 Recruitment

Each study centre will be expected to recruit 50 or more participants in phases 1 to 3.

Up to 15 participants will be recruited for phase 4 which will take place in Bern only.

Once the subjects have agreed to participate in the study, the following activities will be performed by the research team:

- written informed consent
- checking inclusion and exclusion criteria
- medical (diabetes) history
- body weight measurement
- record of current type and dosage of insulin therapy
- urine pregnancy test (females of childbearing potential)

9.2.1 Informed Consent (Phase 3 only)

Written informed consent will be obtained where possible, from the patient or from the closest relative/next of kin if the patient is incapacitated. If the patient lacks capacity and no next of kin is available, written informed consent will be obtained from a senior member of the clinical team responsible for the patient, who is not a member of the study team. In the situation where a subject is enrolled without consent, this should be explained to the next of kin/legal guardian at the earliest opportunity and permission should be sought for continuation of the study. Should the subject/next of kin express subsequent disagreement with study enrolment, the subject will be immediately withdrawn from the study and all his/her data destroyed.

In the situation where the study was conducted with consent from the next of kin / legal guardian of a subject who lacks capacity at the time, permission should be sought from the subject for the use of data obtained during the study, once the subject regains capacity, up to 1 month after the end of the study or on discharge from hospital, whichever occurs earlier. If the subject is unwilling at this stage, all data obtained during the study will be destroyed.

9.3 Randomisation

Eligible subjects, once consented by the research team, will be randomised to the use of automated closed-loop glucose control or to standard care possibly using the minimization method(35), implemented in the minim program (36) to balance, for example, surrogates of insulin resistance such as body mass index and pre-study insulin (units) per kilogram of body weight.

Subjects will be allocated to one of two groups: Group 1, conventional insulin therapy or Group 2, fully automated closed-loop system. During closed-loop, subject's glycaemic control will be managed by subcutaneous insulin pump therapy, driven by computer-based algorithm.

In phase 4, eligible subjects will be randomised using permuted block randomisation to either automated closed-loop control using faster-insulin aspart or automated closed-loop control using standard insulin aspart. Randomisation will take place at visit 1 by an independent nurse who will prepare syringes with "faster-acting insulin aspart (Fiasp)" or "standard insulin aspart (Novorapid)" based on randomiser software output and pre-fill the insulin pump accordingly. Accordingly, both researcher and study participants will be blinded to treatment allocation. The randomisation list is kept electronically in a password protected file. The study team will have no access to the list until the primary analysis of the trial is finished. Unblinding is considered only when knowledge of the treatment assignment is deemed essential for the participant's care by the clinician or regulatory authority. It will therefore be restricted to emergency situations and is expected to be extremely rare in this study. Code breaks/emergency envelopes will be held at the department of nephrology where an on-call nurse will be available to perform the code break at any time. Only healthcare professionals involved in the care of the study participant can require unblinding. The responsible nephrology nurse will inform the principal investigator of any request for unblinding and the reasons for the request. The investigator responsible for the care of the study participant will record the unblinding and reasons in the CRF. The responsible nephrology nurse will also record any unblinding and the reasons in the code list.

9.4 Closed-loop Insulin Delivery versus Conventional Treatment

9.4.1 Fully Closed-loop Insulin Delivery

9.4.1.1 Introduction

During the fully closed-loop intervention, the control algorithm will direct between meals and meal-related insulin delivery. The subcutaneous insulin pump will deliver rapid-acting insulin analogue or ultra-rapid-acting insulin analogue (phase 3 and phase 4). During phase 1, once daily subcutaneous insulin glargine will also be administered to each participant.

9.4.1.2 System Overview

- The control algorithm in the automated mode will be commenced following insertion and calibration of CGM by a member of the research team.
- Non-identifiable study ID, body weight in kilograms and total daily insulin will be used to initiate the closed-loop system.
- The CGM device continuously measures interstitial glucose concentrations and transmits the measurements every minute using radiofrequency link to the CGM Receiver device.
- The CGM Receiver extracts glucose measurements and sends the glucose measurements to an ultra-mobile personal OQO computer or similar, known as the Control Algorithm Device (CAD). The CAD will be running the software containing the control algorithm.
- The control algorithm will calculate the required insulin infusion rate every 10 to 12 minutes, based on historical glucose measurements and historical insulin infusion delivery.
- Every 10 to 12 minutes, the CAD will instruct the insulin pump via wireless communication to alter insulin delivery to the calculated insulin infusion rate. On receiving instruction from the algorithm, the insulin pump delivers the rapid-acting insulin analogue or ultra-rapid-acting insulin analogue (phase 3 and phase 4) subcutaneously.
- No meal announcements will be provided to the control algorithm and no prandial insulin bolus will be given for meals.
- The whole cycle is repeated and this sequence of events will continue for 72 hours.

9.4.2 Conventional Therapy

During the conventional therapy, subject's insulin dose and regimen on admission will be adjusted as necessary by the clinical team according to usual clinical practice at each study centre.

9.5 Phase 1 Sequence of Procedures: Fully Closed-loop Insulin Delivery

A flow diagram illustrating the sequence of procedures for the 72-hour period is shown in Figure 4.

9.5.1 Day 1

- A CGM device will be inserted by a member of the research team on the ward the day the CL system is to be commenced.
- CGM calibration will be performed as per manufacturer's instructions.
- Participants will be given their usual insulin therapy the day before, but will be asked to withhold their insulin therapy on the day of the study.
- Weight will be measured in kilograms using calibrated electronic scales.
- Urine pregnancy test will be carried out in females of childbearing potential
- The CL system will be commenced following calibration of CGM.

- Insulin glargine will be delivered subcutaneously once daily, at 20% of the participant's pre-study daily insulin dose.
- Point of care capillary glucose will be measured for reference glucose values using NovaStat Strip glucose meter or similar before each mealtime and before bedtime.
- Participants will be allowed to continue with their usual oral hypoglycaemic therapy, with the exception of sulphonylurea agents, which will be discontinued during the study.
- Participants will be given their usual standard ward meals, as chosen by the participants themselves, at the usual meal times in the general ward.
- In between meals, participants will be unrestricted in terms of their usual activity in the ward. Participants will be able to choose when they want to sleep according to individual preferences.

9.5.2 Day 2

- Insulin glargine will be delivered subcutaneously once daily, at 20% of the participant's pre-study daily insulin dose.
- Participants will be allowed to continue with their usual oral hypoglycaemic therapy and/or non-insulin injectable therapy, with the exception of sulphonylurea agents, which will be discontinued during the study.
- As per Day 1, participants will be given their usual standard ward meals, as chosen by the participants themselves, at the usual meal times in the general ward.
- As per Day 1, participants will be unrestricted in terms of their usual activity in the ward. Participants will be able to choose when they want to sleep according to individual preferences.

9.5.3 Day 3

- Insulin glargine will be delivered subcutaneously once daily, at 20% of the participant's pre-study daily insulin dose.
- Participants will be allowed to continue with their usual oral hypoglycaemic therapy, with the exception of sulphonylurea agents, which will be discontinued during the study.
- As per Day 1 and Day 2, participants will be given their usual standard ward meals, as chosen by the participants themselves, at the usual meal times in the general ward.
- As per Day 1 and Day 2, participants will be unrestricted in terms of their usual activity in the ward. Participants will be able to choose when they want to sleep according to individual preferences.

9.5.4 Day 4

- At the end of the 72-hour period, CL insulin delivery will be discontinued and the final point of care capillary glucose measurement for the study will be taken.
- Participants will recommence their usual insulin medication(s) according to the following order:
    - Basal alone insulin therapy: Basal insulin will be given at the end of the study.

- Basal-bolus insulin therapy: Basal insulin will be given at the end of the study. Subsequent prandial bolus insulin doses will be given as per usual main meal times.
- Biphasic (premixed) insulin therapy: The first biphasic insulin dose will be given at the same time as the participant's next main meal. The second biphasic insulin dose will be given as per the participant's usual regime.
- Oral anti-diabetic agents: Oral anti-diabetic agents will be given as per the usual inpatient regime.

- Participants will be invited to complete a questionnaire asking about their experience during the study, and their views on the diabetes treatment received.

9.6 Phase 1 Sequence of Procedures: Conventional Therapy

A flow diagram illustrating the sequence of procedures for the 72-hour period is shown in Figure 5.

9.6.1 Day 1

- A CGM device (blinded) will be inserted by a member of the research team on the ward the day the study is to be commenced.
- CGM calibration will be performed as per manufacturer's instructions.
- Participant's insulin dose and regimen will be adjusted as necessary by the clinical team according to Cambridge University Hospital Foundation Trust's usual clinical practise.
- Weight will be measured in kilograms using calibrated electronic scales.
- Urine pregnancy test will be carried out in females of childbearing potential
- Point of care capillary glucose will be measured for reference glucose values using NovaStat Strip glucose meter or similar before each mealtime and before bedtime
- Participants will be given their usual standard ward meals, as chosen by the participants themselves, at the usual meal times in the general ward.
- In between meals, participants will be unrestricted in terms of their usual activity in the ward. Participants will be able to choose when they want to sleep according to individual preferences.

9.6.2 Day 2 and Day 3

- As per Day 1, participants will be given their usual standard ward meals, as chosen by the participants themselves, at the usual meal times in the general ward.
- As per Day 1, participants will be unrestricted in terms of their usual activity in the ward. Participants will be able to choose when they want to sleep according to individual preferences.

9.6.3 Day 4

- At the end of the 72-hour period, the final point of care capillary glucose measurement for the study will be taken. The CGM device will be removed by a member of the research team.
- Participants will continue with their usual anti-diabetic medication(s) and their glucose control will continue to be managed by the clinical team.
- Participants will be invited to complete a questionnaire asking about their experience during the study, and their views on the diabetes treatment received.

9.7 Phase 2 and Phase 3 Sequence of Procedures: Fully Closed-loop Insulin Delivery

A flow diagram illustrating the sequence of procedures for is shown in Figure 6.

9.7.1 Day 1

- Baseline blood samples will be taken (Switzerland only).
- A CGM device will be inserted by a member of the research team on the ward the day the CL system is to be commenced.
- CGM calibration will be performed as per manufacturer's instructions.
- Participants will be given their usual insulin therapy the day before, but will be asked to withhold their insulin therapy on the day of the study.
- Weight will be measured in kilograms using calibrated electronic scales.
- Urine pregnancy test will be carried out in females of childbearing potential.
- The CL system will be commenced following calibration of CGM.
- Point of care capillary glucose will be measured for reference glucose values using NovaStat Strip glucose meter or similar before each mealtime and before bedtime.
- Participants will be allowed to continue with their usual oral hypoglycaemic therapy, with the exception of sulphonylurea agents, which will be discontinued during the study.
- Participants will be given their usual standard ward meals, as chosen by the participants themselves, at the usual meal times in the general ward.
- Daily carbohydrate intake will be recorded in a food diary.
- In between meals, participants will be unrestricted in terms of their usual activity in the ward. Participants will be able to choose when they want to sleep according to individual preferences.
- If closed-loop system needs to be disconnected for more than 1 hour due to inpatient investigation such as magnetic resonance imaging (MRI). Subcutaneous basal insulin injection at 20% of their usual total daily insulin dose will be administered prior to disconnecting the closed-loop system.

9.7.2 Day 2 until end study

- As per Day 1, participants will be allowed to continue with their usual oral hypoglycaemic therapy, with the exception of sulphonylurea agents, which will be discontinued during the study.
- As per Day 1, participants will be given their usual standard ward meals, as chosen by the participants themselves, at the usual meal times in the general ward.
- Daily carbohydrate intake will be recorded in a food diary.
- As per Day 1, participants will be unrestricted in terms of their usual activity in the ward. Participants will be able to choose when they want to sleep according to individual preferences.
- As per Day 1, if closed-loop system needs to be disconnected for more than 1 hour due to inpatient investigation such as magnetic resonance imaging (MRI). Subcutaneous basal insulin injection at 20% of their usual total daily insulin dose will be administered prior to disconnecting the closed-loop system.
- Participant will continue the study until discharge from hospital, or up to maximum of 15 days.

9.7.3 End of Study

- End of study blood samples will be collected (Switzerland only).
- Staff workload burden will be assessed using questionnaires (Switzerland only). Questionnaires will be distributed, per participant, to a minimum of two ward staff caring for the participant during the study.
- At the end of the study period, CL insulin delivery will be discontinued and the final point of care capillary glucose measurement for the study will be taken.
- If participant was on insulin therapy pre-study, participants will recommence their usual insulin medication(s) according to the following order:
    - Basal alone insulin therapy: Basal insulin will be given at the end of the study.
    - Basal-bolus insulin therapy: Basal insulin will be given at the end of the study. Subsequent prandial bolus insulin doses will be given as per usual main meal times.
    - Biphasic (premixed) insulin therapy: The first biphasic insulin dose will be given at the same time as the participant’s next main meal. The second biphasic insulin dose will be given as per the participant’s usual regime.
    - Oral anti-diabetic agents and non-insulin injectables: Oral anti-diabetic agents and non-insulin injectables will be given as per the usual inpatient regime.
- Participants will be invited to complete a questionnaire asking about their experience during the study, and their views on the diabetes treatment received.

9.7.4 Temporary discontinuation of closed-loop insulin delivery

In the event of the participant undergoing surgery or other medical interventions (eg haemodialysis), the decision to stop or continue closed-loop insulin delivery will be at the discretion of the treating clinical team.

9.8 Phase 2 and Phase 3 Sequence of Procedures: Conventional Therapy

A flow diagram illustrating the sequence of procedures for the study period is shown in Figure 7.

9.8.1 Day 1

- Baseline blood samples will be taken (Switzerland only).
- A CGM device (blinded) will be inserted by a member of the research team on the ward the day the study is to be commenced.
- CGM calibration will be performed as per manufacturer’s instructions.
- Participant’s insulin dose and regimen will be adjusted as necessary by the clinical team according to each centre's usual clinical practice.
- Weight will be measured in kilograms using calibrated electronic scales.
- Urine pregnancy test will be carried out in females of childbearing potential.
- Point of care capillary glucose will be measured for reference glucose values using NovaStat Strip glucose meter or similar before each mealtime and before bedtime.
- Participants will be given their usual standard ward meals, as chosen by the participants themselves, at the usual meal times in the general ward.
- In between meals, participants will be unrestricted in terms of their usual activity in the ward. Participants will be able to choose when they want to sleep according to individual preferences.
- Daily carbohydrate intake will be recorded in a food diary.

9.8.2 Day 2 until end of study

- As per Day 1, participants will be given their usual standard ward meals, as chosen by the participants themselves, at the usual meal times in the general ward.
- As per Day 1, participants will be unrestricted in terms of their usual activity in the ward. Participants will be able to choose when they want to sleep according to individual preferences.
- Daily carbohydrate intake will be recorded in a food diary.
- Participant will continue the study until discharge from hospital, or up to maximum of 15 days.

9.8.3 End of Study

- At the end of the study, the final point of care capillary glucose measurement for the study will be taken. The CGM device will be removed by a member of the research team.
- End of study blood samples will be collected (Switzerland only).
- Staff workload burden will be assessed using questionnaires (Switzerland only). Questionnaires will be distributed, per participant, to a minimum of two ward staff caring for the participant during the study.
- Participants will continue with their usual anti-diabetic medication(s) and their glucose control will continue to be managed by the clinical team.

Figure 4. Study Protocol Diagram during Closed-Loop Insulin Delivery in Phase 1

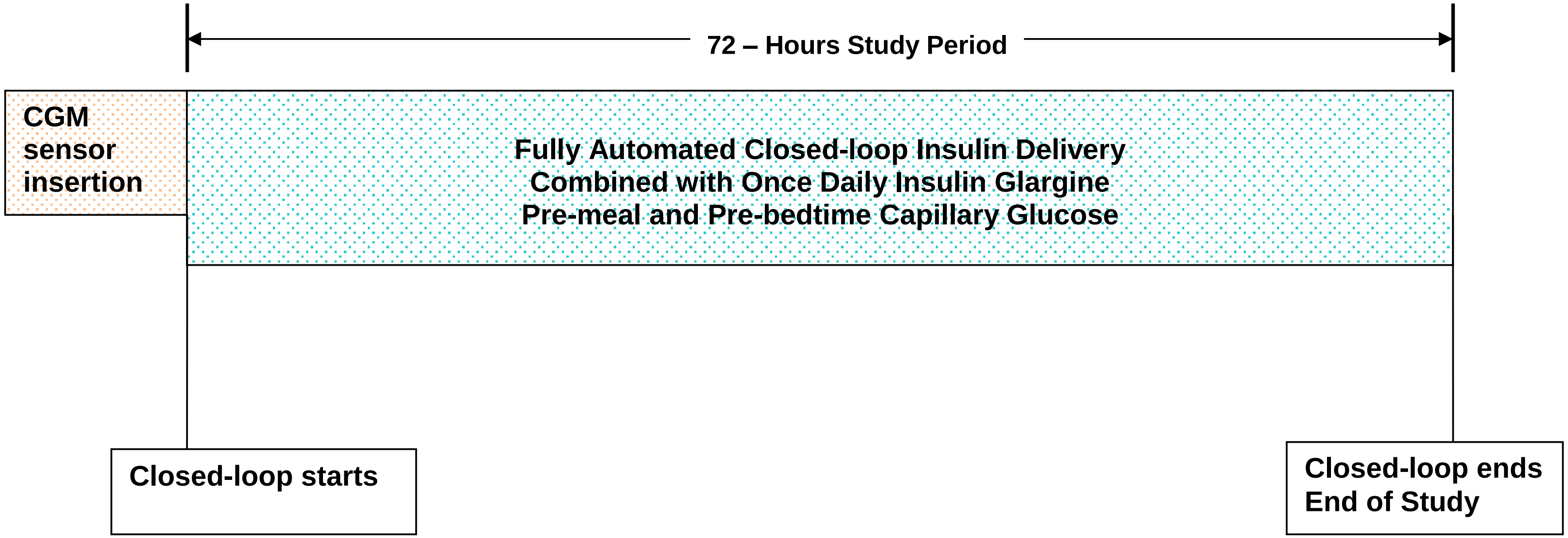

Figure 5. Study Protocol Diagram during Conventional Insulin Therapy in Phase 1

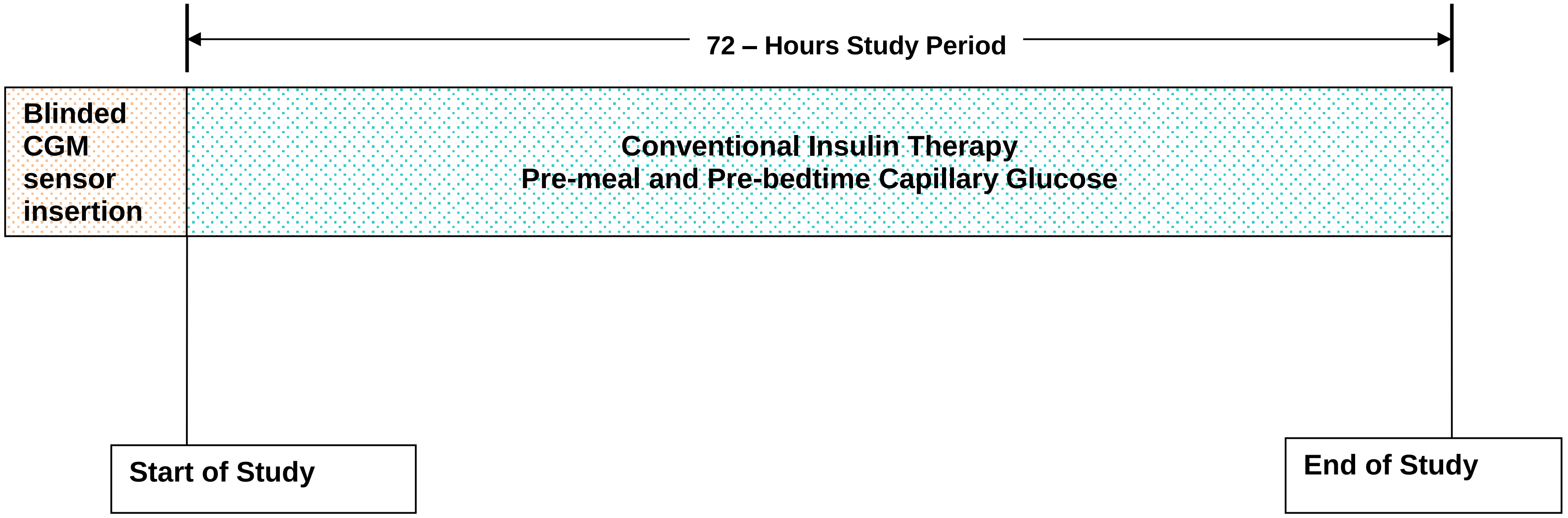

Figure 6. Study Protocol Diagram during Closed-Loop Insulin Delivery in Phase 2 and Phase 3

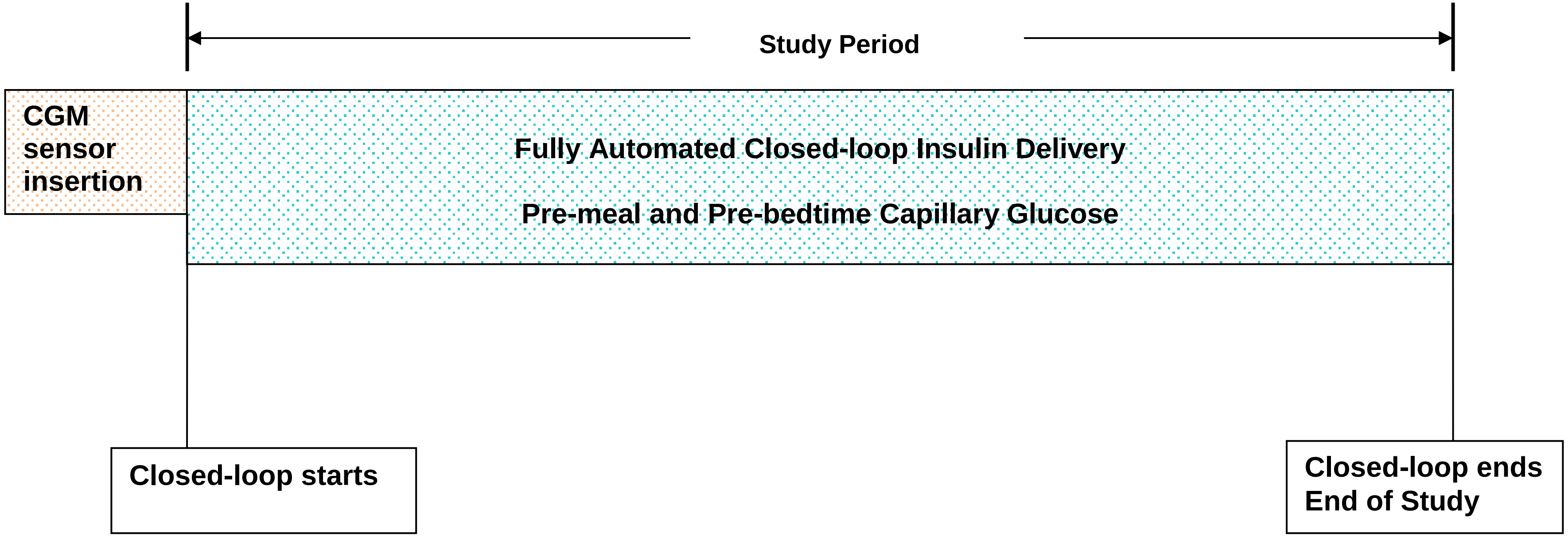

Figure 7. Study Protocol Diagram during Conventional Insulin Therapy in Phase 2 and Phase 3

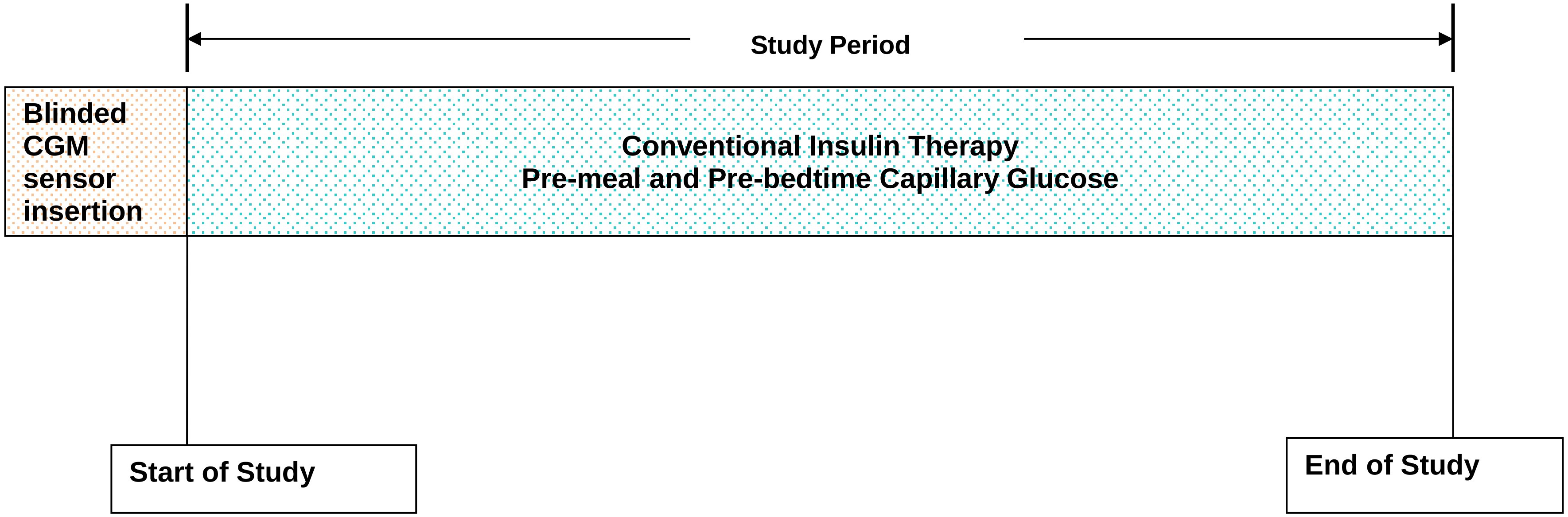

9.9 Phase 4: Sequence of Procedures

The participants will attend the inpatient clinical research facility at the nephrology ward for two 24-hour visits, with the same study procedures performed on both occasions, once using the closed-loop system with faster-acting insulin aspart (intervention) and once using the closed-loop system with standard insulin aspart (control). The order in which subjects attend for the intervention and control visits will be randomly assigned, with the second visit carried out within a 1-4 week time interval.

A flow diagram illustrating the sequence of procedures for the study period is shown in Figure 8. The time schedule of visits 1 and 2 is provided in Appendix 16.

9.9.1 Visit 1 and Visit 2

Participants will be asked to arrive at the facility at 18:30. They will be instructed to have had an early dinner before admission with their usual insulin dose, but withhold their long-acting insulin administration 24 hours prior to the visit. Participants will be allowed to continue with their usual oral hypoglycaemic therapy, with the exception of sulphonylurea agents, which will be discontinued 24 hours before arrival at the facility.

Weight will be measured in kilograms using calibrated electronic scales, height will be measured in metres, and BMI calculated. Urine pregnancy test will be carried out in females of childbearing potential.

The study CGM device will be inserted and calibrated by a member of the research team as per manufacturer's instructions. A subcutaneous cannula will be inserted in the abdomen for delivery of faster-acting insulin aspart or standard insulin aspart by the study pump. Non-identifiable study ID, body weight in kilograms and total daily insulin will be used to initiate the closed-loop system.

An intravenous cannula will be inserted into forehand/arm vein for sampling and will be kept patent with 0.9% saline. Participants will be asked to sleep until 06:30 the following day. The next day, subjects will be given standardised meals, matched for macronutrient content on both visits: breakfast (50g of carbohydrates) at 07:00 and lunch (80g of carbohydrates) at 12:00. Participants will be instructed to have the meals within 15min. Three different breakfast and lunch menus will be offered and choices will be replicated on the second visit. Decaffeinated tea or coffee and water will be offered with meals. Water will be available ad libitum throughout the experiment. Between 10:30 and 11:00, participants will engage in a 20min walk in the ward corridor accompanied by a staff member and will otherwise remain sedentary. Automated closed-loop glucose control will be stopped at 17:30 and intravenous cannula, insulin pump and CGM device will be removed.

9.9.2 Blood Sampling

Blood samples will be taken for the measurement of plasma glucose and plasma insulin.

Plasma glucose will be measured at regular intervals starting at 07:00
- Every 15min for 3h post-meal intake
- Every 30min at other times (10:00-12:00 and 15:00 to 17:00)

Plasma insulin will be measured at regular intervals starting at 07:00
- Every 15min for 3h post-meal intake
- Every 30min at other times (10:00-12:00 and 15:00 to 17:00)

A total of 33 blood samples will be drawn per study visit amounting to a total blood volume of 80ml used per visit. Thus, the total blood volume used for both visits (separated by 1-4 weeks) will amount to 160ml.

9.9.3 End of Study

After device removal on visits 1 and 2, participants will resume their usual diabetes treatment regime according to the instruction from the clinical investigator. Participants will leave the facility, after ensuring blood glucose levels are stable.

Figure 8. A flow diagram illustrating the sequence of procedures of visits 1 and 2 of phase 4.

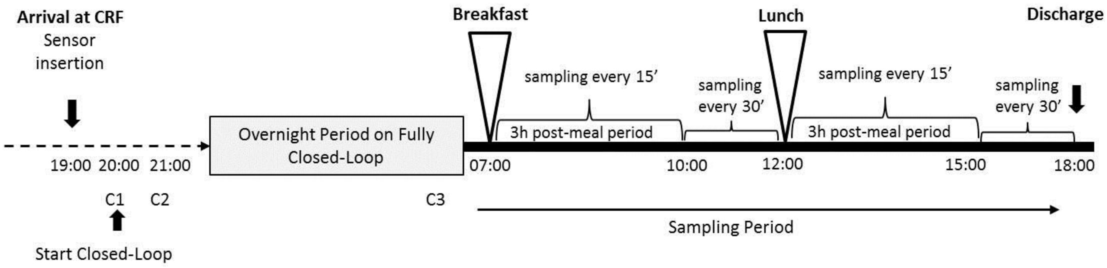

9.9.4 Subject Reimbursement

Phase 4: Subjects will be offered CHF 200 for the time and inconvenience related to taking part in the study. Travel and other relevant visit-related expenses will be reimbursed.

9.10 End of Study Procedures

Having completed the intervention arm of the study, subjects will revert to conventional glucose control therapy and will be managed by the clinical team as per hospital guidelines.

9.11 Experiment Stopping Rules

9.11.1 Termination of Subject's Participation in Study

(1) If more than 3 capillary samples in a row are not obtained (phase 1-3).
(2) If the continuous subcutaneous insulin infusion (CSII) is interrupted for more than 4 hours.
(3) If severe hypoglycaemia occurs (capillary (phase 1-3) or plasma (phase 4) glucose levels below 2.2 mmol/l).
(4) If blood ketones rise above 1.5mmol/l.
(5) If clinical team deems it necessary to switch from CSII to IV insulin infusion therapy due to severe hyperglycaemia.
(6) If clinical team deems it necessary to switch from CSII to IV insulin infusion therapy out of clinical necessity (phase 1-3).
(7) If surgery is planned during the study period (phase 1-3).
(8) Any serious adverse event deemed by the research team to be related to the study.

Phase 4
If the study is stopped due to one of the reasons stated above, the subject may repeat the study visit on another occasion within the 1-4 week time interval.

9.11.2 Withdrawal of Study Subject Data from Final Analysis

All randomised subjects (including withdrawn subjects) data will be included in the final analysis, in accordance with the intention to treat principle. Data from withdrawn subjects will be kept in the coded format.

10 Endpoints

10.1 Efficacy endpoints

10.1.1 Primary efficacy endpoints

The primary outcome measure is time spent in target glucose range (5.6-10.0mmol/l) for 72-hours (phase 1), up to maximum 15 days (phase 2 and phase 3) or during 10-hour period (phase 4), using continuous subcutaneous glucose monitoring data (phase 1-3) or plasma glucose data (phase 4).

10.1.2 Secondary efficacy endpoints

Secondary outcomes will include:

1. Proportion of time with glucose levels in the target glucose range (5.6-10.0mmol/l) as recorded by reference (fingerstick) glucose values.
2. Proportion of time with glucose levels below 5.6 mmol/l and above 10.0 mmol/l as recorded by CGM and reference glucose values.
3. Proportion of time with glucose levels below 3.5 mmol/l as recorded by CGM and reference glucose values.
4. Average glucose levels, as recorded by CGM and reference glucose values.
5. Standard deviation of glucose levels, as recorded by continuous subcutaneous glucose monitoring and point-of-care capillary glucose meter.
6. AUC below 3.5 mmol/l as recorded by CGM.
7. Total daily insulin dose (U/24h) (phase 1-3) or insulin delivered between 07:00 and 17:00
   .

<u>Phase 4 only</u>

8. 2-hour postprandial incremental glucose
9. Peak plasma/sensor glucose
10. Mean of plasma insulin
11. Tmax of plasma insulin
12. Cmax of plasma insulin
13. Total and endogenous insulin exposure within 1 hour postprandial period (iAUC)

10.1.3 Other outcomes of interest

1. Daily carbohydrate intake (g/24h)
2. Staff workload burden assessment using questionnaires (Switzerland only, phase 2 only)

3. Metabolic and inflammatory profiling on serum samples (Switzerland only, phase 2 only)
4. Insulin pharmacokinetics for both Fiasp and standard aspart (phase 4 only)

10.2 Safety Evaluation

Safety evaluation will include frequency of hypoglycaemic episodes as well as the number of subjects experiencing severe hypoglycaemia and other adverse events, including significant hyperglycaemia (>20.0 mmol/l).

11 Safety Evaluation and Reporting

11.1 Definitions

11.1.1 Reportable Adverse Event

A reportable Adverse Event (AE) is any untoward medical occurrence that meets criteria for a serious adverse event. All device deficiencies (DD) that could have led to a serious adverse device effect (SADE) will also be reported (ISO 14155: 8.2.5 and 9.8).

11.1.2 Adverse Event

An adverse event is any untoward medical occurrence, unintended disease or injury, or any untoward clinical signs (including abnormal laboratory findings) in participants, users or other persons whether or not related to the investigational medical device (ISO 14155: 3.2). In accordance with ISO 14155: 3.2 Note 1 and Note 2, this includes events related to:

- Investigational medical device or the comparator
- The procedures involved.

11.1.3 Adverse Device Effect

An adverse device effect (ADE) is an adverse event related to the use of an investigational medical device (ISO 14155: 3.1). In accordance with ISO 14155: 3.1 Note 1 and Note 2, this includes adverse events resulting from:

- Insufficient or inadequate instructions for use, deployment, implantation, installation or operation, or any malfunction of the investigational device
- Use error or intentional misuse of the investigational device.

11.1.4 Device Deficiency

A device deficiency is an inadequacy of a medical device related to its identity, quality, durability, reliability, safety or performance, such as malfunction, misuse or use error and inadequate labelling (ISO 14155: 3.15).

11.1.5 Serious Adverse Event

A serious adverse event is an adverse event that:

- led to death, or
- led to serious deterioration in health that either resulted in:
    - a life-threatening illness or injury, or
    - a permanent impairment of a body structure or a body function, or
    - in-patient or prolonged hospitalisation, or

  - medical or surgical intervention to prevent life threatening illness or injury or permanent impairment to a body structure or a body function
- led to foetal distress, foetal death or a congenital abnormality or birth defect (ISO 14155: 3.37).

A planned hospitalisation for pre-existing condition, or a procedure required by the study protocol, without a serious deterioration in health, is not considered to be a serious adverse event.

11.1.6 Serious Adverse Device Effect

A Serious Adverse Device Effect (SADE) is an adverse device effect that has resulted in any of the consequences characteristic of a serious adverse event or that might have led to a serious adverse event if:

- suitable action had not been taken, or
- intervention had not been made, or
- circumstances had been less opportune.

11.1.7 Unanticipated and Anticipated Serious Adverse Device Effect

An unanticipated serious adverse device effect (USADE) is an adverse device effect which by its nature, incidence, severity or outcome has not been identified in the current version of the protocol or the risk analysis report (ISO 14155: 3.42).

This includes unanticipated procedure related serious adverse events; that is, serious adverse events occurring during the study procedure that are unrelated to any malfunction or misuse of the investigational medical device.

An Anticipated Serious Adverse Device Effect (ASADE) is a serious adverse device effect which by its nature, incidence, severity or outcome has been identified in the protocol or the risk analysis report.

11.1.8 Adverse Event Intensity

**Intensity**	**Definition**
Mild	Patient is aware of signs and symptoms but they are easily tolerated
Moderate	Signs / symptoms cause sufficient discomfort to interfere with usual activities

Severe	Patient is incapable to work or perform usual activities

NB. The term “severe” is often used to describe the intensity (severity) of a specific event. This is not the same as ‘serious’, which is based on patient/event outcome or action criteria (see definition 11.1.5). For example, itching for several days may be rated as severe, but may not be clinically serious.

11.1.9 Adverse Event Causality

Causality	Definition
Not assessable	A report suggesting an adverse event, which cannot be judged because information is insufficient or contradictory, and which cannot be supplemented or verified.
Unlikely	A clinical event, including laboratory test abnormality, with a temporal relationship, which makes a causal relationship improbable, and in which other drugs/treatments, chemicals or underlying disease(s) provide plausible explanations.
Possible	A clinical event, including laboratory test abnormality, with a reasonable time sequence to administration of the treatment/use of investigational treatment/device, but which also could be explained by concomitant diseases or other drugs/treatments or chemicals.
Probable	A clinical event, including laboratory test abnormality, with a reasonable time sequence to administration of the treatment/use of medical method/device, unlikely to be attributable to concomitant disease(s) or other drugs/treatments or chemicals, and which follows a clinically reasonable response on withdrawal (dechallenge). Rechallenge information is not required to fulfil this definition.
Certain	A clinical event, including laboratory test abnormality, occurring in a plausible time relationship to study treatment/use of medical method/device and which cannot be explained by concomitant disease(s), other drugs/treatments or chemicals. The response to withdrawal of the treatment

	(dechallenge) should be clinically plausible. The event must be unambiguously either pharmacologically or as phenomenon, using in satisfactory rechallenge procedures if necessary.

(Reference: WHO-UMC Causality Categories)

11.2 Monitoring Period of Adverse Events

The period during which adverse events will be reported is defined as the period from the beginning of the study (obtaining informed consent) until 72 hours after the end of the intervention. Adverse events that continue after the subject's discontinuation or completion of the study will be followed until their medical outcome is determined or until no further change in the condition is expected. The follow up of AEs may therefore extend after the end of the clinical investigation; however no new AEs will be reported after the trial reporting period.

11.3 Monitoring Period of Serious Adverse Events

In the unlikely event of a serious adverse event, this will be followed-up until the outcome is determined, or until no further change in the event is anticipated.

11.4 Recording of Adverse Events/Device Deficiencies

Throughout the course of the study, all efforts will be made to remain alert to possible adverse events, device deficiencies and safety issues which will be recorded in a secure central online reporting repository, which is accessible to clinical investigators at all study sites. All adverse events and device deficiencies will be documented in both the local site's CRF and the central online reporting repository by clinical investigators. Recording of events in the central online reporting repository will be performed at each site within seven days of discovering the event. The first concern will be the safety of the subject, and appropriate medical intervention will be taken.

The study investigator will assess the relationship of any adverse event to be related or unrelated by determining if there is a reasonable possibility that the adverse event may have been caused by the study device or study procedures. The individual investigator at each site will be responsible for managing all adverse events/reactions according to local protocols, and decide if reporting is required.

11.5 Severe Hypoglycaemia

Severe hypoglycaemia will be defined as a capillary glucose level < 2.2 mmol/l, or an event requiring assistance of another person to actively administer

carbohydrate, glucagon, or other resuscitative actions. These episodes may be associated with sufficient neuroglycopaenia to induce seizure or coma. If plasma glucose measurements are not available during such an event, neurological recovery attributable to the restoration of plasma glucose to normal is considered sufficient evidence that the event was induced by a low plasma glucose concentration. Severe hypoglycaemia will be regarded as an anticipated serious adverse event and will be reported.

11.6 Reporting Procedures

When reporting adverse events, all pertinent data protection legislation must be adhered to.

The serious advent report should contain the following information*:

1. Study identifier
2. Participant’s unique study number
3. Date of birth
4. Event description
5. Start date of event
6. Laboratory tests used and medical interventions used to treat the SAE
7. Planned actions relating to the event, including whether the study device was discontinued
8. Statement on the patient’s current state of health
9. Criterion for seriousness (i.e. death, life threatening, hospitalisation, disability/incapacity or other)
10. Evaluation of causality (including grade of relatedness) with the following (more than one may apply):
    a. the investigational treatment/medical device
    b. the clinical study/a study specific procedure
    c. other: e. g. concomitant treatment, underlying disease
11. Date of procedure
12. Reporter’s name, date and signature

*In the case of incomplete information at the time of initial reporting, all appropriate information should be provided as soon as this becomes available.

UK only
Relationship of the SAE to the treatment should be assessed by the investigator at site, as should the expected or unexpected nature of any serious adverse reactions.

All **SAEs** and **SADEs** whether or not deemed investigational method/device related or expected must be reported to the Sponsor by telephone within 24 hours (one working day) of the Investigator learning of its occurrence:

Louise Stockley
R&D Department
Cambridge University Hospitals NHS Foundation Trust
Box 277
Hills Road

Cambridge CB2 0QQ
UK
Tel: 01223 348491
Email: louise.stockley@addenbrookes.nhs.uk

A written report must follow within five working days and is to include a full description of the event and sequelae, in the format detailed on the Serious Adverse Event reporting form. If applicable, the Sponsor will notify the competent authority of all Serious Adverse Events in line with pertinent legal requirements.

The Investigator will notify the ethics committee of all Serious Adverse Events in line with pertinent legal requirements. The Investigator will inform the Sponsor about all reports sent to the ethics committee including follow-up information and answers by the ethics committee. The local investigator is responsible for informing other site investigators and the CI of all SAEs.

The Ethics Committee will be notified of all unexpected and related SAEs within 15 days of the occurrence of the event.

Switzerland only
The following events are reported to the Representative of the Sponsor and Principal Investigator within 24 hours upon becoming aware of the event:

- All SAEs and SADEs
- Device deficiencies that might have potentially led to an SAE
- Health hazards that require measures

Professor Christoph Stettler
Department of Diabetes, Endocrinology, Clinical Nutrition and Metabolism
Inselspital, Bern University Hospital
University of Bern, Bern
Switzerland
Tel: 0041 31 632 40 70
Email: Christoph.stettler@insel.ch

SAEs will evaluated by the Representative of the Sponsor and Principal Investigator with regard to causality and seriousness. Device deficiencies are assessed regarding their potential to lead to an SAE.

The Representative of the Sponsor and Principal Investigator reports **SAEs and SADEs** in Switzerland within 7 days to the local Ethics Committee which are

- related or possibly related to the medical device under investigation
- related or possibly related to study procedures

The Representative of the Sponsor and Principal Investigator reports the above both locally and internationally, within 7 days to Swissmedic. In addition, all **device deficiencies that could have led to a SADE** in Switzerland and study centres abroad will be notified to Swissmedic.

Health hazards that require measures are reported within 2 days by the investigator to Ethics Committee and by the Representative of the Sponsor and Principal Investigator to Swissmedic.

The Representative of the Sponsor and Principal Investigator notifies Swissmedic and Ethics Committee of the completion of the study within 90 days (as of last patient, last visit). A discontinuation or an interruption of the trial, and the reasons for this are notified within 15 days. A final report, with contents in accordance with EN ISO 14155, will be submitted within one year of completion to Swissmedic and Local Ethics Committee.

UK and Switzerland A yearly safety update-report of events from all study sites will be submitted by the Investigators to the local Ethics Committee and Regulatory Authority.

Reporting to the DMEC committee will be in accordance with the signed and agreed DMEC Charter.

11.7 Anticipated Adverse Events/Device Effects

11.7.1 Hypoglycaemia and Hyperglycaemia

Potential risks during the study visits are:

- symptomatic hypoglycaemia (possible)
- severe hypoglycaemia (rare)
- symptomatic hyperglycaemia (unlikely)
- diabetic ketoacidosis (rare)

11.7.2 Blood test

Subjects will be required to have a blood test (venepuncture) at baseline (UK and Switzerland) and end of study (Switzerland only). Potential risks include:

- Slight discomfort or bruising at the site (common)
- Excess bleeding at the site (unlikely)
- Infection at the site (rare)

11.7.3 Cannula Insertion (phase 4 only)

Potential risks include:

- Slight discomfort or bruising at the site (common)
- Excess bleeding at the site (unlikely)
- Infection at the site (rare)

11.7.4 Insulin Pump Therapy

Potential risks associated with insulin pump therapy include:

- Slight discomfort at the time of insertion of the insulin delivery cannula (common)
- Slight bruising at the site of insertion (common)
- Bleeding at insertion site (rare)
- Infection at the site of insertion (rare)
- Allergy to insulin (very rare).

11.7.5 Continuous Glucose Monitoring

Potential risks associated with CGM:

- Slight discomfort at the time of insertion of CGM (common)
- Slight bruising at the site of insertion (unlikely)
- Bleeding at insertion site (rare)
- Infection at the site of insertion (rare)

11.7.6 Questionnaires

As part of the study, subjects will complete questionnaires which include feedback on their experience and views of the diabetes treatment received (UK and Switzerland), and for health care professionals (Switzerland only), feedback on the effect on their workload. It is possible that some people may find these questionnaires to be mildly upsetting. Similar questionnaires have been used in previous research and these reactions are uncommon.

11.8 Benefits

It is expected that closed-loop insulin delivery may have an important role in the inpatient management of diabetes in this patient group. However, as this will be a proof-of-concept study, it is likely that subjects will not directly benefit from taking part.

12 Methods and Assessments

12.1 Study Procedures

12.1.1 Height and Weight

The baseline height and weight will be measured at the ward. Height will be measured in metres using a calibrated stadiometer. Weight will be measured in kilograms using calibrated electronic scales.

12.1.2 Point of Care Capillary Glucose Measurements (Phase 1-3)

Capillary glucose measurements will be measured before each meal and before bedtime by trained clinical and research staff members using NovaStat Strip® point of care glucose meter or similar CE-marked glucose meter.

12.1.3 Plasma Glucose (Phase 4 only)

Plasma glucose concentrations will be measured using 25µl plasma samples on a calibrated Biosen C-line analyser (IGZ Instruments AG, Zürich, Switzerland). The intra-assay coefficient of variation (CV) at 4.1mmol/l is 1.5%. The equivalent inter-assay CV at this glucose concentration is 2.8%, and 1.7% at 14.1mmol/l.

12.1.4 Plasma Insulin (Phase 4 only)

Plasma insulin will be measured by a mass spectrometric immunoassay (The Thermo Scientific™ MSIA™, Thermo Fisher Scientific, Reinach, Switzerland) at the central laboratory of University Hospital Bern. Measurements will be performed on a triple quadrupole mass spectrometer (Xevo TQ-S, Waters) coupled to an UPLC Acquity system (Waters). The recorded Limit of Quantification (LOQ) and Lower Limit of Detection (LLOD) values are 15 pM. Inter-and intra-day repeatability are within 3%, and spike-in recoveries range from 96% to 100%.

12.2 Assessment for Safety

Phase 1-3

Capillary glucose will be measured in real-time before each meal and before bedtime. If capillary glucose below 3.5mmol/l is measured, the capillary glucose sampling interval will be reduced to every 15 minutes. In addition, if the low glucose alert from the CGM becomes activated, a capillary glucose sample will also be measured.

In case of a symptomatic hypoglycaemic episode (as judged by the subject and/or nurse/clinician) and/or capillary glucose level < 3.5 mmol/l, 15g rapid-acting carbohydrate in the form of Lucozade or a similar oral preparation will be

given. If capillary glucose levels are not improving after 15 minutes, the oral dose will be repeated, and intravenous infusion of 10% Dextrose will be considered. Details of all hypoglycaemic episodes and the total amount of glucose administered will be recorded. Capillary glucose levels will be measured at 15-minute intervals following any treatment for hypoglycaemia, until recovery (capillary glucose level > 3.5 mmol/l).

Any episode of severe hypoglycaemia, defined as capillary glucose levels below 2.2mmol/l, will lead to termination of the participant's involvement in the study.

During closed-loop in case of capillary glucose level > 24mmol/l or plasma glucose level >18mmol/l (phase 4) for greater than 1-hour, the clinical investigator will administer supplemental rapid-acting insulin analogue at a dose deemed appropriate by the clinical investigator. If capillary glucose levels are not decreasing after one hour administration of supplemental rapid-acting insulin analogue at a dose deemed appropriate by the clinical investigator, the appropriate dose will be repeated once infusion set occlusion or other related issues have been excluded. During conventional therapy, hyperglycaemia will be managed as per usual local clinical practice.

Phase 4

Overnight period:
During the supervised overnight period, the participant's sensor glucose is continuously monitored. Researchers will be notified when sensor glucose level fall below 3.5mmol/l. If hypoglycaemia is confirmed by plasma glucose measurement, treatment will be given as outlined below.

Daytime period (07:00-17:00)
In case of a symptomatic hypoglycaemic episode (as judged by the subject and/or investigator) and/or plasma glucose level < 2.5 mmol/l, an oral dose of 15g rapid-acting carbohydrate will be given. If plasma glucose levels are not improving after 15 minutes, the oral dose will be repeated, and intravenous infusion of 10% Dextrose will be considered. Details of all hypoglycaemic episodes and the total amount of glucose administered will be recorded. Plasma glucose levels will be measured at 15-minute intervals following any treatment for hypoglycaemia, until recovery.

Any episode of severe hypoglycaemia, defined as plasma glucose levels below 2.2mmol/l, will lead to termination of the participant's involvement in the study.

In case of plasma glucose level > 18mmol/l for greater than 1-hour, the clinical investigator will administer supplemental rapid-acting insulin analogue at a dose deemed appropriate by the clinical investigator. If capillary glucose levels are not decreasing after one hour administration of supplemental rapid-acting insulin analogue at a dose deemed appropriate by the clinical investigator, the appropriate dose will be repeated once infusion set occlusion or other related issues have been excluded.

12.3 Assessment for Efficacy

Continuous glucose monitoring during the study for 72-hours (phase 1) or up to 15 days (phase 2) will be performed in each participant to assess for efficacy.

12.4 Questionnaire

Participants will be asked to complete a questionnaire evaluating their experience and views of the diabetes treatment received (UK and Switzerland).

Healthcare professionals will be asked to complete a questionnaire evaluating the impact on workload burden (Switzerland only, phase 2 only).

13 Data Analysis

13.1 Derived Indices

The following primary and secondary endpoints will be calculated:

- Percentage time spent at glucose > 5.6mmol/l and ≤ 10.0mmol/l, to quantify time spent in the target range
- Percentage time spent at glucose ≤ 5.6mmol/l to quantify time spent below target
- Percentage time spent at glucose ≤ 3.5mmol/l to quantify the duration of hypoglycaemia
- Percentage time spent at glucose ≤ 2.8mmol/l to quantify the duration of significant hypoglycaemia
- Percentage time spent at glucose > 10.0mmol/l to quantify the duration of hyperglycaemia
- Percentage time spent at glucose > 20.0 mmol/l to quantify the duration of significant hyperglycaemia
- Mean glucose level
- Standard deviation of glucose to quantify the glucose variability
- Number of hypoglycaemia events (glucose < 3.5mmol/l) will indicate safety
- Episodes of hyperglycaemia > 20 mmol/l
- Number of adverse events and serious adverse events
- Total amount of insulin delivered to assess insulin needs
- Time when closed-loop system is in operation will assess utility
- Responses from staff feedback questionnaire will assess workload burden (Switzerland only, phase 2 only)

Phase 1-3
The secondary endpoints will be calculated for various parts of the day distinguishing between fasting, prandial, and overnight periods. CGM and fingerstick glucose measurements will be used to determine endpoints.

13.2 Evaluative Periods

The primary and secondary measures will be calculated from day 1 until the end of the 72-hour (phase 1), up to maximum 15 days (phase 2 and phase 3) study intervention (minimum 48-hours) or during the 10-hour period (phase 4).

13.3 Sample Size and Power Calculations

Phase 1: Type 1 diabetes studies show that primary endpoint has SD of 21% (20, 23, 37). With 18 subjects per group a difference of 20% between arms will be detected with a power of 80% using two-sided t-test at 5% significance level. A difference of 20% is considered clinically relevant. The group size is increased to 20 to mitigate against possible differences in the SD between outcomes in type 1 and type 2 diabetes.

Phase 2: The SD of the primary endpoint is increased to 39% to reflect increased patient heterogeneity. Given this increased SD and with 60 subjects per group, a difference of 20% between arms will be detected with a power of 80% using two-sided t-test at 5% significance level. Up to a total of 150 participants will be randomised to allow for 120 participants with at least 48 hours of data.

Phase 3: This is an exploratory analysis involving 40 subjects with at least 48 hours of data (20 in each group). The previous phase 1 and phase 2 of the study may not provide reliable information about the within group variability in this particular population receiving parenteral and/or enteral nutrition. No formal power calculations thus apply. The size was chosen to correspond to phase 1 study size.

Phase 4: Due to the lack of data on the efficacy of automated closed-loop glucose control using faster-acting insulin aspart versus standard insulin aspart in adults with type 2 diabetes, formal power calculation is inappropriate. This is an exploratory study not powered for a specific hypothesis test based on data from up to 15 subjects (to allow for complete data of at least 10 subjects). Suggestive trends that do not achieve statistical significance may form the basis of further study.

13.4 Interim Analysis

No interim analysis will be performed.

13.5 Statistical Analysis

In general, a 5% significance level will be used to declare statistical significance. Endpoints will be log-transformed as appropriate to achieve normality.

Severe hypoglycaemic events and significant hyperglycaemia (>20.0 mmol/l) will be tabulated in each treatment group, which will be compared using Fisher's exact test. For purposes of analysis, a severe hypoglycaemic event will be defined as glucose ≤ 2.2mmol/l. These episodes may be associated with

sufficient neuroglycopaenia to induce seizure or coma. If plasma glucose measurements are not available during such an event, neurological recovery attributable to the restoration of plasma glucose to normal is considered sufficient evidence that the event was induced by a low plasma glucose concentration. Severe hypoglycaemia events and significant hyperglycaemia will be tabulated irrespective of whether CGM data are available and irrespective of whether closed-loop was operational.

Intention to treat analysis is performed on data collected during subcutaneous insulin delivery.

14 Study Materials

14.1 Study Products

14.1.1 Insulin

Rapid acting insulin Lispro (Eli Lilly, Indiana, United States) will be used in the insulin pumps in Phase 1. Rapid acting insulin Lispro (Eli Lilly, Indiana, United States) or Aspart (Novonordisk, Copenhagen, Denmark) will be used in the insulin pumps in Phase 2 and Phase 4. In Phase 3 and Phase 4, faster acting insulin aspart (Fiasp, Novo Nordisk, Copenhagen, Denmark) will be applied in the intervention group. Fiasp is insulin aspart (IAsp) in a new formulation containing two additional excipients (L-arginine and Niacinamide) which accelerate absorption after subcutaneous administration(38). At the beginning of 2017, Fiasp received marketing authorisation from the European Commission. Fiasp has the potential to further improve the safety and efficacy of closed-loop insulin delivery systems. The incremental benefit of Fiasp compared to Iasp when applied through an automated closed-loop system will be assessed in phase 4. Long-acting insulin Glargine (Sanofi-Aventis, Paris, France) will be administered (phase 1 only) from prefilled insulin pens. The insulin vials and pens must be stored in a refrigerator at the investigator's site at +2°C to 8°C until used and not allowed to freeze.

14.1.2 Insulin Pump

The DANA Diabecare R® (SOOIL) or similar CE-marked insulin pump will be used.

14.1.3 Continuous Subcutaneous Glucose Monitor

The FreeStyle Navigator® Continuous Glucose Monitoring (CGM) System (Abbott Diabetes Care, Alameda, CA, USA) or similar CE-marked CGM device will be used.

14.1.4 CGM Receiver Device

A device receiving and displaying data from the CGM transmitter, communicating with CAD.

14.1.5 Control Algorithm Device (CAD)

An ultra-mobile personal OQO computer or similar, known as the Control Algorithm Device (CAD) will be running the software containing the control algorithm. The control algorithm will calculate the required insulin infusion rate.

The control algorithm will be based on a previously developed MPC algorithm that has been validated. It was initially developed and used in adult subjects with T1D(32). The automated controller has been used safely and effectively in the closed-loop studies in subjects with T1D(26).

15 Case Report Forms

UK only

The Case Report Form (CRF) is the printed (paper-based) document designed to record all the protocol required information to be reported to the Chief Investigator.

Switzerland only

The CRF will be paper-based and electronic. All data requested on the CRF will be recorded and the recorded data should be consistent with the source documents or the discrepancies should be explained.

CRFs in all centres will be completed in accordance with GCP Guidelines. Corrections to the printed (paper-based) CRF will be performed by striking through the incorrect entry and by writing the correct value next to the data that has been crossed out; each correction will be initialled and explained (if necessary) by the Investigator or the Investigator's authorised staff.

16 Data Management

16.1 Study Monitoring

UK only

The Study Coordinator will ensure that the study is conducted in accordance with GCP through monitoring visits.

Switzerland only

Clinical Trial Unit (CTU) will coordinate study monitoring according to pre-specified Monitoring Plan and will be involved in data management.

Source data and documents will be made accessible to monitors.

16.2 Data Management

UK Only

The Institute of Metabolic Science, Addenbrooke's Hospital, Cambridge, will set up a database for data collection. Personal contact details for each subject linking them to a unique identification number will be held locally on a study screening log. Computers will be password secure and hard copies of data will be held in locked offices. Data will be stored in the Institute of Metabolic Science, Addenbrooke's Hospital for 10 years.

Switzerland only

The eCRF in this trial is implemented using a dedicated electronic data capturing (EDC) system (REDCap). The EDC system is activated for the trial only after successfully passing a formal test procedure.

All data entered in the eCRF are stored on a Linux server in a dedicated mySQL database.
Responsibility for hosting the EDC system and the database lies with CTU Bern.

Designated investigator staff will enter the data required by the protocol into the eCRF. Automatic validation programs check for data discrepancies in the eCRFs and, by generating appropriate error messages, allow modification or verification of the entered data by the investigator staff before transfer to the RedCap server located at the CTU.

Continuous data from devices will be downloaded as ASCII text files will be stored on a SharePoint 2013 platform that was centrally set up by the University Hospital Bern. It fulfils all requirements of the Human Research Act (HRA). The physical location of the servers is in Zollikofen BE.

The SharePoint server is kept in a locked server-room. Only the system administrators have direct access to the server and back-up tapes. Each access on the platform occurs only with personal credentials (user name and password). The credentials are managed centrally in the Active Directory System of the Inselspital. An anonymous access to the SharePoint platform is not possible. A role concept (read, read/write and administrator) is realised using SharePoint groups and regulates permission for each user and each research project in order to use the system as he/she requires. Every access to the SharePoint server is recorded by the audit function of the SharePoint Server. This audit trail contains information about the accessing user, date and time of access and the name of the document that was accessed. The tracking of the actual data changes is not included in this audit trail. Therefore, the SharePoint versioning functionality is at all times switched on. The comparison of different versions of research document (using the Excel Inquire add-on of Microsoft Excel 2013) ensures the traceability of data changes. The backup of the server and the database instances is done once a week by 1 full and 6 incremental backups. The retention time is 30 days.

At final analysis, data files will be extracted from the database into statistical packages to be analysed. The status of the database at this time is recorded in special archive tables. CTU Bern will securely store the final study database with all archive tables for at least 15 years. The research data files on the SharePoint platform will remain available to all authorised participants for at least 10 years.

Study participants will be assigned a non-identifiable study-specific code (AH2(S)_ followed by two digits). The patient identification list to link the coded data will be accessible to the study team exclusively. Non-identifiable data may

be shared with collaborating institutions and other third parties for the purposes of advancing management and treatment of diabetes, and for publication purposes.

UK only

The Principal Investigator will keep a patient identification list to link study records with patient personal details. Paper copies of the data will be stored in line with the Data Protection Act 1998.

Switzerland only

The patient identification list to link coded personal data with patient details will be stored separately from data collection (HRO, Art 26 paragraph 2).

Storage of health-related personal data will be performed using appropriate operational and organisational measures to protect it according to ClinO Art. 18.

17 Study Management

17.1 Study Registration

The study will be registered at www.clinicaltrials.gov.

Switzerland only

Registration in a national language in the Swiss Federal Complementary Database (http://kofam.ch) will be performed.

17.2 Categorisation of Study

Switzerland only

The study is considered a Category C Medical Device Clinical Trial, as the investigational product (closed-loop insulin delivery system) is not CE-marked at present.

17.3 Study Steering Committee

A study steering committee (SSC) will supervise the trial, to ensure it is conducted to high standards in accordance with the protocol, the principles of GCP, and with regard to participant safety. This committee will consist of the Chief Investigator (Dr Roman Hovorka) and Clinical Investigators (Dr. Anthony Coll, Dr Hood Thabit, Professor Christoph Stettler, and Dr Lia Bally).

The SSC will meet at regular intervals during active phase, and at the conclusion of the study. The SSC will consider the study and relevant information from other sources, ensuring at all times that ethical considerations are met when recommending the continuation of the trial.

18 Ethics

18.1 Ethics Committee

Prior to commencement of the study, the protocol, any amendments, subject information/informed consent form, any other written information to be provided to the subject, subject recruitment procedures, current investigator CVs, and any other documents as required by the Ethics Committee will be submitted. Written approval will be obtained from the Ethics Committee prior to the commencement of the study.

The study will be conducted in accordance to the protocol and with principles enunciated in the Declaration of Helsinki, the guidelines of Good Clinical Practice, the European Directive on Medical Devices 93/42/EEC and the ISO

Norm 14155 and ISO 14971 and the local regulatory and authority requirements.

18.2 Declaration of Interest

There is no commercial or conflict of interest with industry, related to the study.

18.3 Informed Consent of Study Subjects

In obtaining and documenting informed consent, the investigator will comply with the applicable regulatory requirements and will adhere to ICH GCP Guidelines and to the ethical principles that have their origin in the Declaration of Helsinki. Prior to the start of the study, the Investigator will obtain favourable ethical opinion of the written informed consent form and any other written information to be provided to subjects and/or their legally acceptable representative.

Subjects and/or their legally acceptable representative will be given full verbal and written information regarding the objective and procedures of the study and the possible risks involved. Subjects and/or their legally acceptable representative will be informed about their right to withdraw from the study at any time. The signed informed consent forms will be photocopied, originals filed in the Investigator's Site File, and a copy placed in the patient's medical notes and a copy given to the subjects and/or their legally acceptable representative.

18.4 Protocol Amendments

The Sponsor and Representative of the Sponsor may amend the protocol or provide suggestions for a protocol amendment. Protocol modifications related to the indication of use of the investigational device or study population will be agreed by the Manufacturer.

Switzerland only

Substantial amendments are only implemented after approval of the Ethics Committee (ClinO, Art. 29) and Swissmedic (ClinO, Art 34) respectively.

Under circumstances in which the rights, safety and well-being of participants need to be protected, deviations from the protocol may proceed without prior approval of the Ethics Committee and Swissmedic. Such deviations shall be documented and reported to the Ethics Committee and Swissmedic as soon as possible. All non-substantial amendments will be communicated to Swissmedic as soon as possible if applicable and to the Ethics Committee within the Annual Safety Report.

19 Timetable

Phase 1
Inclusion of the first subject in the study is planned to take place in March 2013. The expected completion of the last subject and the planned completion of the Clinical Study Report is December 2013.

Phase 2
Inclusion of the first subject in the study took place in August 2016. The expected completion of the last subject is August 2018.

Phase 3
Eligible subjects will be enrolled between January 2018 and December 2018.

Phase 4
Eligible subjects will be enrolled between April 2018 and December 2018.

20 Deviations from Protocol

Deviations from the protocol should not occur. If deviations do occur, they will be documented, stating the reason and the date, the action taken, and the impact for the subject and for the study. The documentation will be kept in the Investigator's Site File.

21 Responsibilities

21.1 Principal Investigator

The Principal Investigator (PI) is the person with overall responsibility for the research and all ethical applications will be submitted by the PI. The PI is accountable for the conduct of the study and if any responsibilities are delegated. The PI should maintain a list of appropriately qualified persons to whom he/she has delegated specified significant study-related duties.

22 Reports and Publications

Data will be published in internationally peer-reviewed scientific journals and disseminated in lay communications; members of the investigator group will all be co-authors. Decision to submit the report for publication, authorship eligibility, intellectual property management, and sharing of study data results will be outlined in Study Agreements between Sponsors and respective study sites. Intellectual property of the automated closed-loop insulin delivery system will remain with the University of Cambridge.

23 Retention of Study Documentation

UK only

Subject notes must be kept for the maximum of time period as permitted by each individual NHS Trust. Other source documents and the Investigator's Site File must be retained for at least 15 years. The Principal Investigator will archive the documentation pertaining to the study in an archive after completion or discontinuation of the file.

Switzerland only

The Representative of the Sponsor and Principal Investigator will retain all data pertaining for a minimum of 10 years after completion or discontinuation of the trial (Art 45 ClinO of HRA). Access will be restricted to research team members, Swissmedic and Ethics Committee.

24 Indemnity Statements

The clinical investigators are indemnified to cover negligent harm to patients participating in the study by their membership of medical defence organisations.

25 Insurance

Switzerland only

Study insurance will be provided by the University Hospital of Bern. A copy of the certificate will be filed in the investigator site file and the trial master file.

26 References

1. Lind L, Lithell H. Impaired glucose and lipid metabolism seen in intensive care patients is related to severity of illness and survival. Clin Intensive Care. 1994;5(3):100-5.
2. Stranders I, Diamant M, van Gelder RE, Spruijt HJ, Twisk JW, Heine RJ, et al. Admission blood glucose level as risk indicator of death after myocardial infarction in patients with and without diabetes mellitus. Arch Intern Med. 2004;164(9):982-8.
3. Williams LS, Rotich J, Qi R, Fineberg N, Espay A, Bruno A, et al. Effects of admission hyperglycemia on mortality and costs in acute ischemic stroke. Neurology. 2002;59(1):67-71.
4. Baker EH, Janaway CH, Philips BJ, Brennan AL, Baines DL, Wood DM, et al. Hyperglycaemia is associated with poor outcomes in patients admitted to hospital with acute exacerbations of chronic obstructive pulmonary disease. Thorax. 2006;61(4):284-9.
5. Umpierrez GE, Isaacs SD, Bazargan N, You X, Thaler LM, Kitabchi AE. Hyperglycemia: an independent marker of in-hospital mortality in patients with undiagnosed diabetes. J Clin Endocrinol Metab. 2002;87(3):978-82.
6. Murad MH, Coburn JA, Coto-Yglesias F, Dzyubak S, Hazem A, Lane MA, et al. Glycemic control in non-critically ill hospitalized patients: a systematic review and meta-analysis. J Clin Endocrinol Metab. 2012;97(1):49-58.
7. van den Berghe G, Wouters P, Weekers F, Verwaest C, Bruyninckx F, Schetz M, et al. Intensive insulin therapy in the critically ill patients. N Engl J Med. 2001;345(19):1359-67.
8. Finfer S, Chittock DR, Su SY, Blair D, Foster D, Dhingra V, et al. Intensive versus conventional glucose control in critically ill patients. N Engl J Med. 2009;360(13):1283-97.
9. Umpierrez GE, Hellman R, Korytkowski MT, Kosiborod M, Maynard GA, Montori VM, et al. Management of hyperglycemia in hospitalized patients in non-critical care setting: an endocrine society clinical practice guideline. J Clin Endocrinol Metab. 2012;97(1):16-38.
10. Schnipper JL, Barsky EE, Shaykevich S, Fitzmaurice G, Pendergrass ML. Inpatient management of diabetes and hyperglycemia among general medicine patients at a large teaching hospital. J Hosp Med. 2006;1(3):145-50.
11. Giangola J, Olohan K, Longo J, Goldstein JM, Gross PA. Barriers to hyperglycemia control in hospitalized patients: a descriptive epidemiologic study. Endocr Pract. 2008;14(7):813-9.
12. Bailon RM, Cook CB, Hovan MJ, Hull BP, Seifert KM, Miller-Cage V, et al. Temporal and geographic patterns of hypoglycemia among hospitalized patients with diabetes mellitus. J Diabetes Sci Technol. 2009;3(2):261-8.
13. Amrein K, Ellmerer M, Hovorka R, Kachel N, Parcz D, Korsatko S, et al. Hospital glucose control: safe and reliable glycemic control using enhanced model predictive control algorithm in medical intensive care unit patients. Diabetes Technol Ther. 2010;12(5):405-12.
14. Hovorka R. Closed-loop insulin delivery: from bench to clinical practice. Nat Rev Endocrinol. 2011;7(7):385-95.

15. Thabit H, Hartnell S, Allen JM, Lake A, Wilinska ME, Ruan Y, et al. Closed-loop insulin delivery in inpatients with type 2 diabetes: a randomised, parallel-group trial. Lancet Diabetes Endocrinol. 2016.
16. Blaha J, Kopecky P, Matias M, Hovorka R, Kunstyr J, Kotulak T, et al. Comparison of three protocols for tight glycemic control in cardiac surgery patients. Diabetes Care. 2009;32(5):757-61.
17. Feichtner F, Mader JK, Schaller R, Schaupp L, Ellmerer M, Korsatko S, et al. A Stepwise Approach toward Closed-Loop Blood Glucose Control for Intensive Care Unit Patients: Results from a Feasibility Study in Type 1 Diabetic Subjects Using Vascular Micro dialysis with Infrared Spectrometry and a Model Predictive Control Algorithm. J Diabetes Sci Technol. 2011;5(4):901-5.
18. Wilinska ME, Chassin LJ, Acerini CL, Allen JM, Dunger DB, Hovorka R. Simulation environment to evaluate closed-loop insulin delivery systems in type 1 diabetes. J Diabetes Sci Technol. 2010;4(1):132-44.
19. Wilinska ME, Budiman ES, Taub MB, Elleri D, Allen JM, Acerini CL, et al. Overnight closed-loop insulin delivery with model predictive control: assessment of hypoglycemia and hyperglycemia risk using simulation studies. J Diabetes Sci Technol. 2009;3(5):1109-20.
20. Hovorka R, Allen JM, Elleri D, Chassin LJ, Harris J, Xing D, et al. Manual closed-loop insulin delivery in children and adolescents with type 1 diabetes: a phase 2 randomised crossover trial. Lancet. 2010;375(9716):743-51.
21. Kumareswaran K, Harris J, Allen J, Elleri D, Nodale M, Hayes J, et al. Overnight closed-loop insulin delivery in adults with type 1 diabetes. Diabetes UK, Liverpool UK. 2010.
22. Kumareswaran K, Harris J, Elleri D, Allen J, Nodale M, Weston J, et al. Overnight Closed Loop (CL) Glucose Control Following Consumption of Alcohol in Adults with Type 1 Diabetes. Diabetes. 2010;59(A95-A).
23. Hovorka R, Kumareswaran K, Harris J, Allen JM, Elleri D, Xing D, et al. Overnight closed loop insulin delivery (artificial pancreas) in adults with type 1 diabetes: crossover randomised controlled studies. BMJ. 2011;342:d1855.
24. Elleri D, Allen J, Kumareswaran K, Leelarathna L, Nodale M, Caldwell K, et al. Day-and-night closed-loop glucose control in adolescents with type 1 diabetes. Advanced Technologies & Treatments for Diabetes Conference 2011; 2011; London, UK; 2011.
25. Murphy H, Kumareswaran K, Allen J, Elleri D, Simmons D, Raymond-Barker P, et al., editors. Closed loop insulin delivery protects against nocturnal hypoglycaemia after moderate physical activity in pregnant women with type 1 diabetes. Diabetes UK; 2011; London, UK; 2011.
26. Elleri D, Allen J, Nodale M, Wilinska M, Mangat J, Larssen A, et al. Automated overnight closed-loop glucose control in young children with type 1 diabetes. Diabetes Technol Ther. 2011;13.
27. Takahashi G, Sato N, Matsumoto N, Shozushima T, Hoshikawa K, Akitomi S, et al. Preliminary study on glucose control with an artificial pancreas in postoperative sepsis patients. Eur Surg Res. 2011;47(1):32-8.
28. Kansagara D, Fu R, Freeman M, Wolf F, Helfand M. Intensive insulin therapy in hospitalized patients: a systematic review. Ann Intern Med. 2011;154(4):268-82.
29. Unger J, Parkin C. Hypoglycemia in insulin-treated diabetes: a case for increased vigilance. Postgrad Med. 2011;123(4):81-91.

30. Moghissi ES, Korytkowski MT, DiNardo M, Einhorn D, Hellman R, Hirsch IB, et al. American Association of Clinical Endocrinologists and American Diabetes Association consensus statement on inpatient glycemic control. Diabetes Care. 2009;32(6):1119-31.
31. Inzucchi SE. Clinical practice. Management of hyperglycemia in the hospital setting. N Engl J Med. 2006;355(18):1903-11.
32. Hovorka R, Chassin LJ, Wilinska ME, Canonico V, Akwi JA, Federici MO, et al. Closing the loop: the adicol experience. Diabetes Technol Ther. 2004;6(3):307-18.
33. Hovorka R, Canonico V, Chassin LJ, Haueter U, Massi-Benedetti M, Orsini Federici M, et al. Nonlinear model predictive control of glucose concentration in subjects with type 1 diabetes. Physiol Meas. 2004;25(4):905-20.
34. Clement S, Braithwaite SS, Magee MF, Ahmann A, Smith EP, Schafer RG, et al. Management of diabetes and hyperglycemia in hospitals. Diabetes Care. 2004;27(2):553-91.
35. Scott NW, McPherson GC, Ramsay CR, Campbell MK. The method of minimization for allocation to clinical trials. a review. Control Clin Trials. 2002;23(6):662-74.
36. **Evans S**, **Day S**, **Royston** P. **Minim: minimisation program for allocating patients to treatments in clinical trials. http://www-users.york.ac.uk/~mb55/guide/minimins.doc**Minim: minimisation program for allocating patients to treatments in clinical trials. http://www-users.york.ac.uk/~mb55/guide/minimins.doc.
37. Hovorka R, Nodale M, Haidar A, Wilinska ME. Assessing Performance of Closed-Loop Insulin Delivery Systems by Continuous Glucose Monitoring: Drawbacks and Way Forward. Diabetes Technol Ther. 2012.
38. Heise T, Hövelmann U, Brøndsted L, Adrian CL, Nosek L, Haahr H. Faster-acting insulin aspart: earlier onset of appearance and greater early pharmacokinetic and pharmacodynamic effects than insulin aspart. Diabetes Obes Metab. 2015;17(7):682-8.

27 Appendices

27.1 Appendix 1

Overnight Closed-Loop (CL) Insulin Delivery in Children and Adolescents with Type 1 Diabetes (T1D): Towards Home Testing

R Hovorka, CL Acerini, JM Allen, LJ Chassin, C Kollman, D Elleri, J Harris, T Hovorka, AM Larsen, M Nodale, A De Palma, ME Wilinska, D Xing, and DB Dunger
Institute of Metabolic Sciences and Department of Paediatrics, University of Cambridge, United Kingdom; JAEB Centre, Tampa, FL, USA

We executed 3 CL inpatient studies (APCam01, 02 & 03) in young people with T1D. In total, 17 subjects were studied (M 8; age 13.5±3.6 years, BMI 21.0±4.0 kg/m^2, duration diabetes 6.4±4.1 years, A1C 8.5±1.8%) over 33 nights with CL and 21 nights with CSII. APCam01 compared CL against CSII (N = 12; self-selected meal and prandial insulin at 18:00; CL from 20:00 to 08:00). APCam02 evaluated CL after large slowly and rapidly absorbed meals (N = 6; 129±34 g CHO meal with prandial insulin at 18:00; CL from 18:30 to 08:00). APCam03 compared CL and CSII following exercise (N = 9; 55% VO_2MAX exercise from 18:00h until 18:45 with 5min rest; CL from 20:00 to 08:00). Real-time subcutaneous (sc) continuous glucose monitoring (CGM) data was fed into a model predictive controller every 15 minutes, which calculated sc insulin infusion for manually adjusted insulin pump. Reference plasma glucose was measured every 15min. During CL, no rescue carbohydrates were administered. As shown in Table, CL improved glucose control and reduced risk of hypoglycaemia. Figure shows cumulative PG and CGM distributions during APCam01, 02 & 03 from start of CL (values in brackets are from midnight). We conclude that inpatient overnight CL in children and adolescents with T1D is safe and efficacious, and improves significantly glucose control compared to CSII. Outpatient testing is next goal.

Table. Overnight closed loop vs CSII during APCam01 & 03 (N = 21)

	Closed loop	CSII	P
Plasma glucose (mM)	*7.0 (#6.3)	8.1 (7.0)	NS (NS)
Plasma glucose ≤ 3.9mM (%)	6.8 (6.3)	20 (28)	0.01 (0.002
Plasma glucose in target 3.9 – 8.0mM (%)	66 (77)	38 (40)	0.01 (0.007
Plasma glucose > 8mM (%)	28 (17)	43 (32)	NS (NS)
LBGI	1.6 (1.7)	3.8 (5.4)	0.01 (0.002
Insulin infusion (U/h)	1.1 (1.0)	1.1 (1.0)	NS (NS)
* from 20:00 to 08:00; # from 00:00 to 08:00			

Figure. Plasma glucose and CGM during closed loop in APCam01, 02 & 03 (N = 33) from start of control and after midnight. Time spent below, at, and above target (3.9 – 8.0mM) is displayed at top.

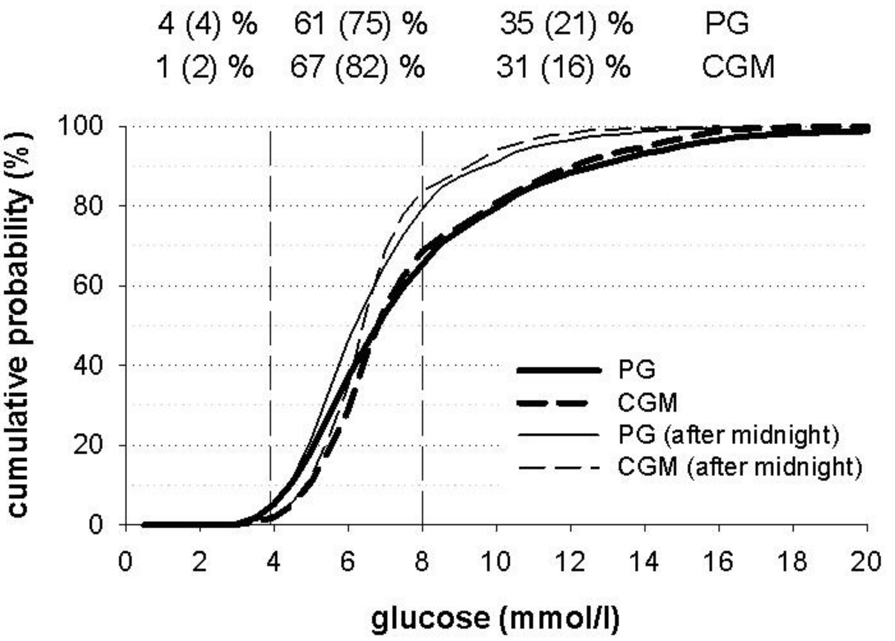

27.2 Appendix 2

Overnight sc-sc closed-loop control improves glucose control and reduces risk of hypoglycaemia in children and adolescents with type 1 diabetes

ROMAN HOVORKA, CARLO L. ACERINI, JANET ALLEN, LUDOVIC J. CHASSIN, ANNE M. LARSEN, ALESSANDRA DE PALMA, MALGORZATA E. WILINSKA, DAVID B. DUNGER, *Cambridge, United Kingdom*

We investigated the ability of closed-loop insulin delivery (CL) to improve overnight blood glucose (BG) control and to reduce risk of hypoglycaemia in 12 children and adolescents with T1D (M 7; age 13.1±4.2 years, BMI 21.9±4.3 kg/m^2, duration diabetes 6.8±4.4 years, HbA1C 8.7±2.0%). Subjects were studied on two separate nights 1 – 3 wks apart and were randomized to receive either overnight CL or standard CSII therapy. Following dinner and prandial insulin at 18:00h, CL was performed between 20:00 – 08:00 the next day. Real-time subcutaneous (sc) continuous glucose monitoring (CGM) data was fed into a model predictive controller (MPC) every 15 minutes, which calculated sc insulin infusion for manually adjusted insulin pump. On CSII night, subject's standard insulin pump settings were applied and non-real-time CGM data recorded. On each night BG was measured every 15min and analyzed on study completion. No rescue carbohydrates were administered. *CGM data*. CL concentrated sc glucose in target range 3.9 to 8.0mM (Figure) and improved glucose control while reducing risk of hypoglycaemia, see Table which reports Kovatchev's Low BG Index (LBGI), Chassin's Grade A+B efficacy rating, and Grade E+F hypo/hyperglycaemia risk index. *BG data*. CL was associated with increased time spent in target range (61 (45 – 73) vs CSII 35 (19 – 53)%, p = 0.03). Improvements in LBGI failed to reach statistical significance (0.9 (0.2 – 3.7) vs 2.0 (0.2 – 9.5), p = NS) due to CGM overestimation of low BG values.
[figure1]
[table1]
Conclusions. In children and adolescents with T1D, 12h overnight CL with MPC increases sc glucose "in target" time by 37% and reduces hypoglycaemia risk index eight-fold.

Tables:

Comparison of CL and CSII using sc glucose (mean±SD or median (IQR))

	CL	CSII	P
Glucose at 20:00 (mM)	10.6 ± 3.0	11.9 ± 4.8	NS
Mean overnight glucose (mM)	7.8 ± 1.4	8.4 ± 3.6	NS
Time in target (%)	63 (49 – 78)	26 (23 – 35)	0.002
LBGI	0.3 (0.0 – 1.0)	2.6 (0.0 – 8.1)	0.02
Grade A+B (%)	52 (32 – 66)	18 (10 – 27)	0.01
Grade E+F (%)	0 (0 – 1)	37 (4 – 45)	0.01

Insulin infusion (U/h)	1.1 (0.6 – 1.8)	1.2 (0.6 – 2.0)	NS

Images:

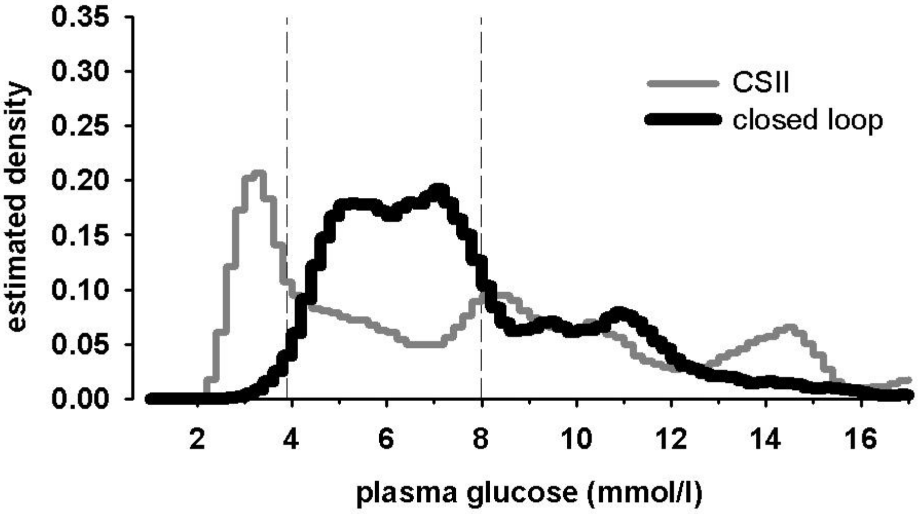

27.3 Appendix 3

Good overnight closed-loop glucose control in children and adolescents with type 1 diabetes following ingestion of large, rapidly and slowly absorbed dinner

Roman Hovorka, Carlo L Acerini, Janet Allen, Ludovic J. Chassin, Debra Mundt,
Anne Mette F Larsen, Alessandra De Palma, Malgorzata E Wilinska, and David B Dunger

Institute of Metabolic Sciences and Department of Paediatrics, University of Cambridge, United Kingdom.

Background and Aims:
In type 1 diabetes (T1D), large evening meal is accompanied by large prandial insulin dose increasing the risk of nocturnal hyper- and hypoglycaemia due CHO/prandial insulin mismatch. We investigated overnight closed-loop (CL) insulin delivery in children and adolescents with T1D to evaluate the performance of CL following ingestion of rapidly and slowly absorbed large dinner.

Materials and Methods:
Six children and adolescents with T1D treated by CSII (M 1; age 15.1±2.2 years; BMI 22.8±4.5 kg/m^2; duration diabetes 6.6±4.4 years; A1C 9.1±2.4%; basal dose 27±13 U/day; bolus dose 27±18 U/day; mean±SD) received overnight CL at a clinical facility on two separate nights 1 – 4 wks apart. In random order, the subjects ate either rapidly or slowly absorbed dinner matched for carbohydrates (CHO) (rapid vs slow: CHO 129±34 vs 129±34 g; glycaemic load 113±29 vs 40±8; energy distribution 68:12:20 vs 54:29:17% CHO:fat:protein; recommended daily energy intake 32±6 vs 41±11%). Following dinner and prandial insulin (17±6 vs 17±7 U; rapid vs slow) at 18:00h, CL was performed between 18:30 – 08:00 the next day. Real-time subcutaneous (sc) continuous glucose monitoring (CGM) data was fed into a model predictive controller (MPC) every 15 minutes, which calculated sc insulin infusion for manually adjusted insulin pump. Blood glucose (BG) was sampled every 15min and measured with 3h delay.

Results:
CGM data. Following rapidly absorbed dinner, 4h incremental glucose area-under-curve (IAUC) was significantly higher (Table). This was accompanied by a non-significant increase in CL 4h insulin delivery. From midnight, glucose excursions were comparable, see Figure showing median (IQ range). Overall, overnight glucose control was good and comparable between two study nights as assessed by mean glucose, time in glucose target 3.9 – 8.0mM, Kovatchev's Low BG Index (LBGI), Chassin's Grade A+B efficacy rating, and Grade E+F hypo/hyperglycaemia risk index (Table). *BG data*. Compared to CGM data,

similar glucose control was observed except higher LBGI [1.4 (0.3 – 3.5) vs 1.3 (0.9 – 1.5)] due to CGM overestimation of low glucose values.

Conclusions:
In children and adolescents with T1D, 12h overnight CL with MPC results in safe and efficacious glucose control following the ingestion of large dinner irrespective of its composition.

Supported by JDRF (Artificial Pancreas Project), EFSD, and NIHR Cambridge BRC.

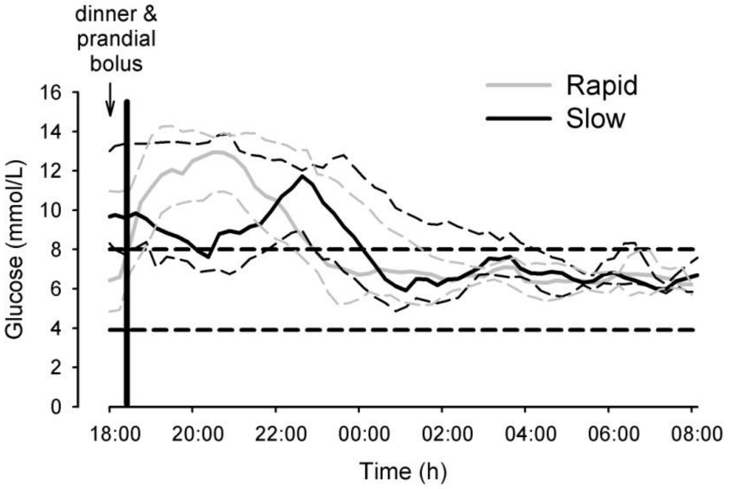

Comparison of CL following rapidly and slowly absorbed dinner [mean±SD or median (IQR)]			
	Rapid	Slow	P
Glucose at 18:30 (mM)	9.0 ±4.4	11.1 ±5.2	NS
Glucose IAUC 18:00 to 22:00 (mM/240min)	798 ± 767	-396 ± 517	0.03
Overnight glucose (mM)	8.4 ±1.9	8.6 ±2.2	NS
Time in target 3.9 – 8.0mM (%)	59 (49 – 63)	61 (43 – 68)	NS
LBGI	0.1 (0.0-1.1)	0.2 (0.0-0.3)	NS
Grade A+B (%)	45 (40 – 52)	51 (38 – 65)	NS
Grade E+F (%)	0 (0 – 11)	1 (0 – 1)	NS
Overnight insulin infusion (U/h)	1.6 ± 0.5	1.4 ± 0.6	NS
Insulin infusion 18:00 to 22:00 (U/240min)	9.2 ± 4.0	6.7 ± 4.1	NS

27.4 Appendix 4

Overnight closed-loop insulin delivery following afternoon exercise in children and adolescents (CaA) with type 1 diabetes (T1D)

Roman Hovorka, Carlo L Acerini, Janet Allen, Ludovic J Chassin, Ulf Ekelund, Daniela Elleri, Anne Mette F Larsen, Marianna Nodale, Malgorzata E Wilinska, and David B Dunger
Institute of Metabolic Sciences, Department of Paediatrics, and MRC Epidemiology Unit, University of Cambridge, United Kingdom.

We investigated ability of overnight closed-loop (CL) insulin delivery in CaA with T1D to prevent nocturnal hypoglycaemia following afternoon exercise. Nine postpubertal CaA with T1D treated by CSII (M 3; age 14.4±2.1 years; BMI 20.0±2.9 kg/m^2; duration diabetes 5.8±3.3 years; A1C 7.8±1.0%; mean±SD) were studied on two separate nights. Subjects received in random order either overnight CL or CSII therapy. On each occasion, subjects had snack at 16:00 and exercised at 55% VO_2MAX from 18:00 for 40min. CL was performed between 20:00 – 08:00 the next day. Subcutaneous (sc) continuous glucose monitoring data was fed into a model predictive controller (MPC) every 15 minutes, which calculated sc insulin infusion for manually adjusted insulin pump. On CSII night, subject's standard insulin pump settings were applied. CL concentrated sc glucose in target range 3.9 to 8.0mM (Figure and Table). One CSII study was stopped as plasma glucose < 2.0mM. CL tended to reduce Kovatchev's Low BG Index (LBGI) although this failed to reach statistical significance. Table reports Kovatchev's LBGI, Chassin's Grade A+B efficacy rating and Grade E+F hypo/hyperglycaemia risk index. In conclusion, 12h overnight CL with MPC following afternoon exercise avoids 81ypoglycaemia and increases time spent in target glucose range twofold.
Supported by JDRF (Artificial Pancreas Project), EFSD, and NIHR Cambridge BRC.

Table. Comparison of CL and CSII using sc glucose (mean±SD)

	CL	CSII	P
Glucose at 20:00 (mM)	7.9 ± 2.2	11.1 ± 5.2	NS
Mean overnight glucose (mM)	6.6 ± 0.8	7.8 ± 3.9	NS
Time in target 3.9 – 8.0mM(%)	84 ± 20	40 ± 37	0.01
LBGI	0.8 ± 0.7	5.3 ± 8.0	NS
Grade A + B (%)	75 ± 24	29 ± 30	0.004
Grade E + F (%)	1 ± 3	24 ± 26	0.030
Insulin infusion (U/h)	0.8 ± 0.3	0.9 ± 0.4	NS

Figure. Median (IQR) sc glucose.

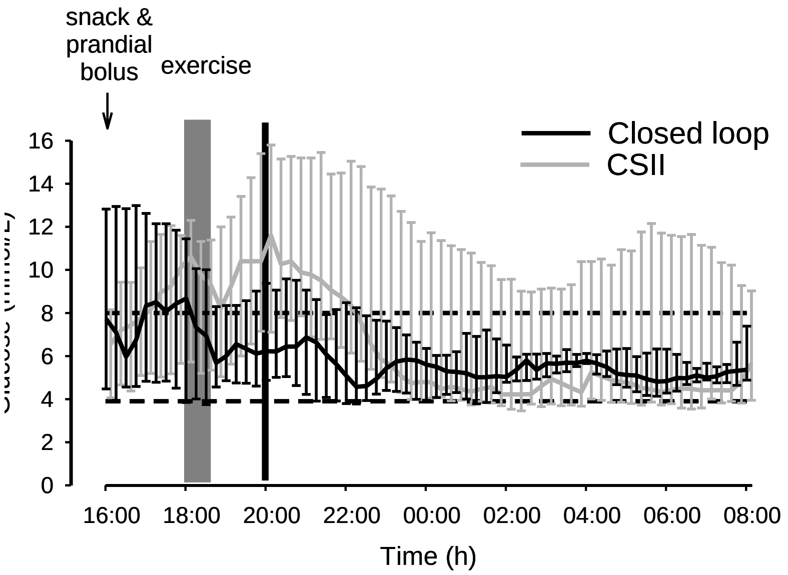

27.5 Appendix 5

Overnight closed-loop insulin delivery in adults with type 1 diabetes

K Kumareswaran[1], J Harris[1], JM Allen[1], D Elleri[1], M Nodale[1], JH Hayes[1], ME Wilinska[1], DB Dunger[1], SR Heller[2], SA Amiel[3], ML Evans[1], R Hovorka[1]

University of Cambridge[1], University of Sheffield[2], King's College London[3]

Aims: To assess feasibility and safety of overnight closed-loop (CL) glucose control compared with conventional continuous subcutaneous insulin infusion (CSII) in adults with Type 1 diabetes (T1D).

Methods: Twelve adult subjects with T1D (F:M 7:5, age 37.7±8.5yrs, diabetes duration 21.5±10.1yrs, HbA1c 7.8±0.5%; mean±SD) treated by CSII, were randomised for two overnight inpatient studies (cross-over design) during which Novorapid insulin was infused using either (i) the CL algorithm linking Freestyle Navigator Continuous Glucose Monitoring System with CSII, or (ii) their usual CSII settings. On both study occasions, subjects consumed a standardised dinner with 60g carbohydrates accompanied by a prandial bolus at 19:00. CL was initiated at 19:00, and continued until 08:00 the following day. Plasma glucose was measured every 15min to assess CL performance.

Results: The time spent in target glucose range (3.9-8.0mmol l^{-1}) was significantly greater during CL compared with conventional CSII (73±28% vs. 55±26%, p=0.006). CL reduced the Low Blood Glucose Index, measuring duration and severity of hypoglycaemia (1.3±1.5 vs. 2.9±2.9, p=0.022). SD of glucose was significantly smaller during CL control (1.2±0.5 vs. 2.0±0.8mmol l^{-1}, p=0.007) indicating reduced glucose variability overnight. Importantly, these improvements in glycaemic control were achieved with no significant difference in the average amount of insulin infused on both study nights (0.7±0.3 vs. 0.7±0.2U h^{-1}, p=NS).

Conclusions: This is the first randomised study of overnight CL glucose control in adults with T1D, demonstrating increased time in target range, reduced hypoglycaemia risk, and reduced glucose variability during CL compared with conventional CSII.

27.6 Appendix 6

Overnight Closed-loop (CL) Glucose Control following Consumption of Alcohol in Adults with Type 1 Diabetes (T1D)

Kavita Kumareswaran[1], Julie Harris[1], Daniela Elleri[1], Janet M Allen[1], Marianna Nodale[1], Joanna Weston, Malgorzata E Wilinska[1], David B Dunger[1], Stephanie A Amiel[2] , Simon R Heller[3], Mark L Evans[1], Roman Hovorka[1]

1Institute of Metabolic Science, University of Cambridge, United Kingdom
[2]King's College London, United Kingdom
[3]University of Sheffield, United Kingdom

We recently demonstrated feasibility and efficacy of overnight CL insulin delivery in adults with T1D, compared with conventional continuous subcutaneous insulin infusion (CSII). Given that alcohol increases risk of nocturnal/ next morning hypoglycemia in T1D, we have now examined how CL impacts on overnight glycaemic control after drinking alcohol. Twelve adults with T1D treated by CSII were studied (F 7, age 37.2±9.9yrs, T1D duration 19.7±9.7yrs, HbA1c 7.8±0.7%; mean±SD). Subjects were studied on 2 separate nights in random order, receiving either overnight CL or CSII therapy. On both occasions, between 2030 and 2200, subjects consumed a mixed meal (100g-carbohydrate) accompanied by 0.75g/kg ethanol as 13% white wine (mean volume consumed 564±133ml). A prandial bolus was administered concurrently. CL was commenced from 2200 until 1200 the next day. Every 15 min, subcutaneous (sc) continuous glucose monitoring (CGM) values from the Freestyle Navigator CGM were fed into a model predictive control algorithm, which calculated the insulin infusion rate to be manually adjusted for delivery via a sc insulin pump. On CSII night, subject's usual insulin pump settings were applied. Plasma glucose was measured every 15min to assess CL performance.

Table. Comparison of CL and CSII using plasma glucose

	CL	CSII	P
Mean overnight glucose (mmol/l)	6.8±0.8	7.0±1.5	NS
SD overnight glucose (mmol/l)	1.8±0.5	2.3±0.7	0.031
Time < 3.9mmol/l (%)	5±7	13±13	NS
Time in target 3.9–8.0mmol/l (%)	72±15	50±23	0.002
Time > 8.0mmol/l (%)	23±16	37±26	0.042
Low BGI	1.4±1.2	2.9±2.6	NS
High BGI	1.8±1.6	3.2±2.6	0.016
Mean overnight insulin infusion (U/h)	0.9±0.4	0.9±0.4	NS

CL significantly improved time in target glucose range (3.9-8.0mmol/l) and reduced glucose variability as measured by SD of glucose, without altering the average insulin infusion rate overnight. CL reduced the high Blood Glucose Index (BGI), measuring duration and severity of hyperglycaemia. There was 1 episode of severe hypoglycemia with CSII and none with CL. In conclusion,

following the consumption of alcohol, CL resulted in improved overnight glucose control compared with conventional CSII.

27.7 Appendix 7

Closing the loop overnight in adults with type 1 diabetes following standard meal and large meal with alcohol

K. Kumareswaran[1]*, J. Harris*[1]*, J. Allen*[1]*, D. Elleri*[1]*, M. Nodale*[1]*, M. Wilinska*[1]*, S. Amiel*[2]*, S. Heller*[3]*, M. Evans*[1]*, R. Hovorka*[1]
[1]Institute of Metabolic Science, University of Cambridge, Cambridge, [2]King's College London, London, [3]University of Sheffield,, Sheffield, UK

Objective: To evaluate an overnight closed-loop (CL) system in adults with type 1 diabetes (T1D).

Methods: Two randomised, crossover studies compared CL with conventional continuous subcutaneous insulin infusion (CSII). In a feasibility study, a medium-sized evening meal containing 60g carbohydrate (CHO) was consumed. In a follow-up study, subjects consumed a large evening meal (100g CHO) accompanied by 0.75g/kg ethanol as 13% white wine (mean intake 564±133ml). Twenty-four subjects (M/F 10/14; age 37.5±9.1years, T1D duration 20.6±9.7years, HbA1c 7.8±0.6%; mean±SD) were studied. During CL nights (n=24), every 15minutes, subcutaneous continuous glucose monitoring values from the Freestyle Navigator CGM System were fed into a model predictive control algorithm, which calculated the infusion rate of Aspart to be adjusted manually for delivery via the Deltec Cozmo insulin pump. During control nights (n=24), subjects' usual insulin pump settings were applied. Plasma glucose was measured every 15minutes to assess CL performance.

Results: Following 60g-CHO meal, overnight CL increased time in target plasma glucose 3.9-8.0mmol/l (80% vs 51% CL vs CSII, p=0.002), with similar outcomes following 100g-CHO meal and alcohol (70% vs 47%, p=0.01). Secondary analysis of pooled data documented increased time in target (76% vs 50%; CL vs CSII, p< 0.001), reduced time < 3.9mmol/l (2.8% vs 6.7%, p=0.04) and > 8.0mmol/l (18% vs 30%, p=0.006), and reduced SD of glucose measuring glycaemic variability (1.4 vs 2.0mmol/l, p=0.001). There was no difference in average overnight insulin infusion (0.8 vs 0.8U/h, p=0.83).

Conclusion: Overnight CL may significantly improve glucose control and reduce risk of nocturnal hypoglycaemia in adults with T1D.

27.8 Appendix 8

Day-and-night Closed-loop (CL) Glucose Control in Adolescents with Type 1 Diabetes (T1D)

D. Elleri[1,2], J.M. Allen[1,2], K. Kumareswaran[1], L. Leelarathna[1], M. Nodale[1], K. Caldwell[1], H. Murphy[1], M.E. Wilinska[1,2], C.L. Acerini[2], D.B. Dunger[1,2] and R. Hovorka[1,2]

[1]Institute of Metabolic Science, University of Cambridge, Cambridge, UK
[2]Department of Paediatrics, University of Cambridge, Cambridge, UK

We evaluated closed-loop (CL) insulin delivery during a 36h period replicating normal daily activities. Twelve adolescents with T1D (M 5; age 15.0±0.9years; A1C 7.9±0.7%; BMI 21.4±2.6kg/m^2; duration of diabetes 6.1±2.8years; total daily dose 1.0±0.4U/kg/day; mean±SD) were studied at a clinical research facility on two occasions. Subjects were randomly allocated to receive either CL or open-loop (OL) (conventional CSII treatment) from 19:30 to 08:00 two days later. During CL, basal rates on insulin pump were manually adjusted every 15min using a model-predictive-control algorithm and real-time CGM glucose levels. On each occasion, subjects engaged in normal daily activities (e.g. playing computer games, walks). They consumed meals (50-80gCHO), accompanied by self-calculated insulin boluses, and snacks (15-30gCHO). Moderate-intensity exercise on a stationary bicycle at 140bpm heart-rate was performed at 10:40 (40min) and at 17:30 (20min). Overall mean plasma glucose levels were 130±22mg/dl during CL versus 162±52mg/dl during OL (p=0.009). Time spent in target glucose range 70-180mg/dl was 82±10% vs 55±29% (p=0.002). Time above 180mg/dl was 13±12% vs 37±33% (p=0.005) and time spent below 70mg/dl was 5±4% vs 7±11% (p=0.50). Overnight, plasma glucose levels were in target for 97±7% vs 51±38% (p=0.02) and for 95±11% vs 45±45% (p=0.001) during the first and second night, respectively. Hypoglycemia occurred on 11 occasions during OL vs 9 during CL (5 episodes were exercise-related, 4 occurred within 2 hours after meals). In conclusion, day-and-night CL may improve glucose control significantly compared to conventional CSII. Exercise and prandial insulin overdosing are confounding factors of hypoglycemia-free glucose control during the day.

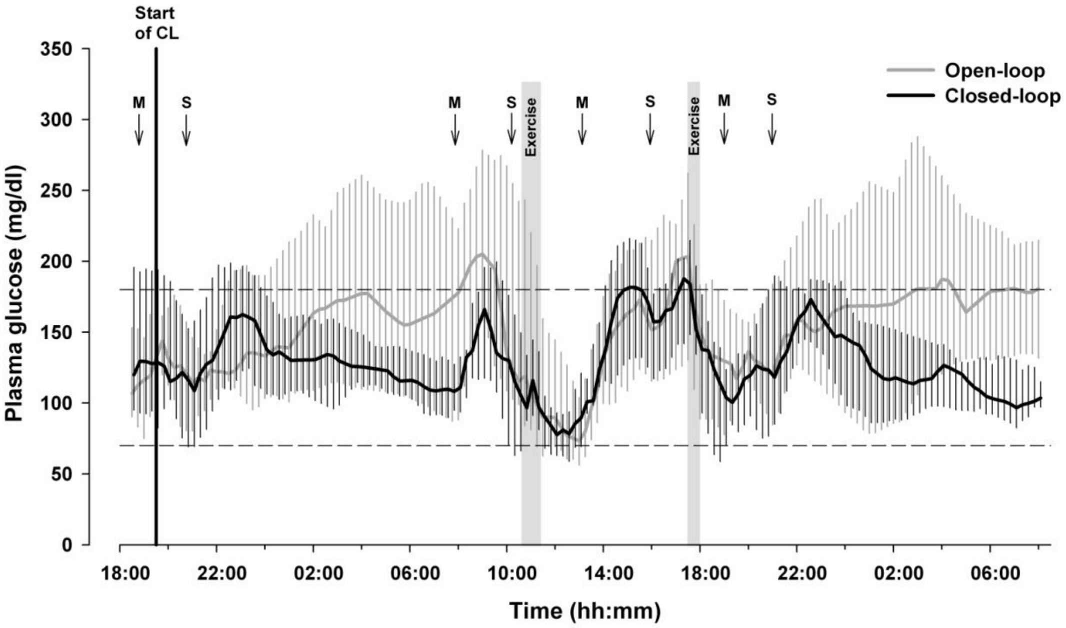

Plasma glucose (median, IQR) during OL (grey line) and CL (black line). Meals (M) and snacks (S) are showed by vertical arrows.

27.9 Appendix 9

Closed-loop insulin delivery protects against nocturnal hypoglycaemia after moderate physical activity in pregnant women with type 1 diabetes (T1DM)

Helen R Murphy, Kavita Kumareswaran, Janet M Allen, Daniela Elleri, David Simmons, Pip Raymond-Barker, Marianna Nodale, Malgorzata E Wilinska, Stephanie Amiel and Roman Hovorka

Aims/Objectives: To examine the safety and efficacy of closed-loop insulin delivery after physical activity in women with T1DM during pregnancy.

Methods: Six women with T1DM between 16-34 weeks gestation were admitted for 24 hours to a clinical research facility. The time spent with plasma glucose levels within the target range (3.5-7.8mmol/l) was determined following afternoon exercise (50min treadmill walking at 15.00h), overnight fast and morning exercise (50min treadmill walking at 09.30h). Prandial insulin boluses were adjusted by women according to their usual algorithms and pre-meal capillary glucose concentration, for lunch (50g CHO 12:30hr), evening meal (60g CHO 18:00hr) and breakfast (50g CHO 07:30hr). Continuous Subcutaneous Insulin Infusion basal rates were adjusted at 15min intervals using an MPC-based computer algorithm and real time CGM glucose levels.

Results: Mean plasma glucose was 6.3 ± 1.0 mmol/l (5.3 ± 2.0 after afternoon exercise, 6.1 ± 0.6 overnight and 6.6 ± 2.0 after morning exercise). Overall time spent with plasma glucose in target was 69.5 ± 13.5% (61.1 ± 30.8% after afternoon exercise, 92.4 ± 8.8% overnight, 53.1 ± 26.2% after morning exercise). Time spent hypoglycaemic (plasma < 2.8 mmol/l) was 1.25 ± 1.5% overall (2.9 ± 5.5% after afternoon exercise, 0% overnight, 3.0 ± 3.8% after breakfast).

Conclusion: While further modification of prandial insulin doses and/or replacement carbohydrates is required for optimal glycaemic control during and immediately after exercise, closed-loop insulin delivery can deliver near-optimal overnight glucose control with no nocturnal hypoglycaemia following moderate physical activity.

27.10 Appendix 10

Fully Automated Overnight Closed-loop Glucose Control in Young Children with Type 1 Diabetes (T1D)

D. Elleri, J.M. Allen, M. E. Wilinska, M..Nodale, C.L. Acerini, D.B. Dunger and R. Hovorka

Institute of Metabolic Science and Department of Paediatrics, University of Cambridge, Cambridge, United Kingdom

Objective
We evaluated fully automated overnight closed-loop (FAOCL) insulin delivery in young children with T1D.

Methods
7 children with T1D (M 4; age 8.9±2.4 years; BMI 17.6±1.4 kg/m^2; duration of diabetes 4.0±2.7 years; total daily dose 0.7±0.1 U/kg/day; A1C 7.8±1.0%; mean±SD) were studied in clinical research facility on two occasions. Subjects had meal at 1800 (76.7±8.6 g CHO) and snack at 21:00 (20.8±6.5 g CHO) accompanied by prandial insulin bolus. In random order, FAOCL started at 18:00 or 21:00 and run until 0800 the next day. Subcutaneous (sc) continuous glucose monitoring (CGM) data was fed automatically into model predictive control algorithm, which calculated sc insulin infusion rates sent wirelessly to insulin pump.

Results
No rescue carbohydrates were administered. Time spent in target glucose range 3.9-8.0 mmol/L [46 (7, 75) vs 60 (23, 67) %; median CGM, (IQR), p=NS, Wilcoxon test] and time above 8.0 mmol/L [36 (25, 93) vs 40 (33, 77) %, p=NS] did not differ between two occasions. Time below 3.9 mmol/L was 0 (0, 0)% on both occasions. Low BG Index (LBGI) (1.2±3.0 vs 0.4±0.5, mean±SD, p=NS), glucose at start of FAOCL (12.9±3.1 vs 13.5±2.5 mmol/L, p=NS) and mean overnight glucose (9.0±2.1 vs 9.0±2.0mmol/L) were also similar. Insulin infusion rates during FAOCL were higher than preprogrammed basal rates on both occasions (0.58±0.25 vs 0.41±0.16, p=0.03 and 0.57±0.21 vs 0.42±0.16U/h, p=0.01).

Conclusions
Fully automated overnight closed-loop is feasible in young children with T1D. Comparable results were obtained when closed-loop was initiated at 1800 or 2100.

27.11 Appendix 11

Closed-loop insulin delivery in well-controlled adults with type 1 diabetes: a free-living day-and-night randomised cross-over study

L. Bally[1, 2, 3*], H. Thabit[1, 2*], H. Kojzar[5], J.K. Mader[5], J. Qerimi-Hyseni[5], S. Hartnell[2], M Tauschmann[1, 4], J.M. Allen[1, 4], M.E. Wilinska[1, 4], T. R. Pieber[5], M. L. Evans[1, 2], R. Hovorka[1, 4]

[1]Wellcome Trust-MRC Institute of Metabolic Science, University of Cambridge, Cambridge, United Kingdom
[2]Department of Diabetes & Endocrinology, Cambridge University Hospitals NHS Foundation Trust, Cambridge, United Kingdom
[3]Department of Diabetes, Endocrinology, Clinical Nutrition & Metabolism, Inselspital, Bern University Hospital, University of Bern, Switzerland
[4]Department of Paediatrics, University of Cambridge, Cambridge, United Kingdom
[5]Department of Internal Medicine, Division of Endocrinology & Diabetology, Medical University of Graz, Graz, Austria

Background: In a population of well-controlled adults with type 1 diabetes, we aimed to establish if day-and-night closed-loop insulin delivery can further improve glucose control whilst alleviating hypoglycaemia risk.
Methods: In an open-label randomised multi-centre crossover study design we compared four-week unsupervised day-and-night closed-loop and usual pump therapy in 29 adults with HbA1c<7.5% under free-living conditions. During closed-loop, model predictive control algorithm directed insulin delivery; prandial insulin delivery was applied using standard bolus wizard. Primary outcome was time when glucose was in the target range of 3·9-10.0mmol/l. Analyses were by intention to treat. Trial registration ClinicalTrials.gov NCT0272731.
Findings: Time when sensor glucose was in target range was significantly increased by 10.5% (95% CI 7.6% to 13.4%, p<0.001; primary endpoint) during closed-loop compared to usual therapy. Closed-loop reduced mean glucose by 0.4mmol/l (95% CI 0.1 to 0.7mmol/l, p=0.023) and reduced the relative risk of time spent below target by 50% (95% CI 37% to 59%, p<0.001) and hypoglycaemia burden measured by AUC<3.5mmol/l by 73% (95% CI 59% to 82%, p<0.001). Glycaemic variability as measured by standard deviation and coefficient of variation of glucose was reduced by closed-loop by 0.5mmol/l (95% CI 0.3 to 0.7mmol/l, p<0.001) and 5% (95% CI 3% to 7%, p<0.001) respectively. Total daily insulin was comparable (p=0.36). No serious adverse events occurred.
Interpretation: Four-week use of unsupervised day-and-night hybrid closed-loop under free-living conditions in well-controlled adults with type 1 diabetes is safe, improves glucose control and reduces hypoglycaemia burden. Larger and longer studies are warranted

27.12 Appendix 12

Fully closed-loop insulin delivery in type 2 diabetes inpatients on general ward: A randomised parallel design study

Hood Thabit[1,2], Sara Hartnell[2], Janet M Allen[1,3], Andrea Lake[2], Malgorzata E. Wilinska[1,3], Yue Ruan[1,3], Mark L. Evans[1,2], Anthony P. Coll[1,2] and Roman Hovorka[1,3]

[1]Wellcome Trust-MRC Institute of Metabolic Science, University of Cambridge, Cambridge, United Kingdom
[2]Wolfson Diabetes Endocrine Clinic, Cambridge University Hospitals NHS Foundation Trust,
Cambridge, United Kingdom
[3]Department of Paediatrics, University of Cambridge, Cambridge, UK

Background: We assessed whether fully closed-loop insulin delivery (the artificial pancreas) is safe and efficacious in type 2 diabetes compared to standard subcutaneous insulin therapy on general ward.
Methods: We enrolled 40 insulin-treated inpatients with type 2 diabetes in single centre, randomised parallel study, comparing (i) closed-loop using model predictive control algorithm to direct subcutaneous insulin delivery of rapid acting insulin analogue without meal bolusing and (ii) conventional subcutaneous insulin therapy according to local clinical guidelines. Primary outcome was time when sensor glucose was in target range between 5.6 and 10.0mmol/l over 72h study duration. Trial registration ClinicalTrials.gov NCT01774565.
Findings: In intention to treat analysis, proportion of time when sensor glucose was in target range was 21.8 percentage points (95% confidence interval [CI] 10.4 to 33.1; p<0.001) greater and time spent above target 19.0 percentage points (95% CI 4.7 to 33.3; p=0.011) lower with the use of closed-loop without changing total daily insulin amount (p=0.78). Overall mean glucose and area under curve below 3.5mmol/l were comparable (p=0.065 and p=0.36, respectively). Closed-loop increased proportion of time overnight when sensor glucose was in target by 20.1 percentage points (95% CI 5.9 to 34.3; p=0.007) whilst area under curve below 3.5mmol/l was not different (p=0.59). No episodes of severe hypoglycaemia or hyperglycaemia with ketonaemia occurred during either study period. Patient-reported acceptability of closed-loop use was high.
Interpretation: Closed-loop insulin delivery without meal bolusing in general ward is safe and feasible in type 2 diabetes adults treated by insulin.

27.13 Appendix 13

Activities at recruitment and study visits

	Recruitment	Day 1	Day 2	Day 3	Day 4
Informed consent	✓				
Check inclusion/exclusion criteria	✓				
Urine pregnancy test (females)	✓				
Medical and diabetes history	✓				
Randomisation	✓				
Weight measurement	✓				
Concomitant illness history	✓				
Glucose sensor insertion		✓			
Insulin pump subcutaneous infusion set insertion		✓			
Once daily subcutaneous basal insulin injection		✓	✓	✓	
Capillary glucose measurement before each mealtime and before bedtime		✓	✓	✓	
Questionnaire					✓
End of study					✓

Activities at recruitment and study visits (Switzerland only)

	Recruitment	Day 1	Day 2 and onwards	End study
Informed consent	✓			
Check inclusion/exclusion criteria	✓			
Urine pregnancy test (females)	✓			
Blood sampling	✓			✓
Medical and diabetes history	✓			
Randomisation	✓			
Weight measurement	✓			
Concomitant illness history	✓			
Glucose sensor insertion		✓		
Insulin pump subcutaneous infusion set insertion		✓		
Capillary glucose measurement before each mealtime and before bedtime		✓	✓	
Dietary assessment		✓	✓	
Questionnaire				✓
End of study				✓

27.14 Appendix 14

Participant Experience Questionnaire (Closed-Loop)
Below is a list of questions which on which we would like your feedback, based on your experience during the study. Please tick the box that best describes your answer.
(**Please do not skip any)**

		Worse than expected	What you expected	Better than expected
Q1.	Were you happy with your glucose levels in hospital during the study?	☐	☐	☐
Q2.	Were you happy with the diabetes treatment given to you during the study?	☐	☐	☐
Q3.	Were you happy with wearing an insulin pump?	☐	☐	☐
Q4.	Were you happy with wearing a glucose sensor?	☐	☐	☐
Q5.	Were you happy to have your glucose levels controlled automatically by the system?	☐	☐	☐
			**Yes**	**No**
Q6.	Did the system limit your mobility whilst in hospital?		☐	☐
Q7.	If a friend or family member was in hospital, would you recommend the system to them?		☐	☐
Q8.	Would you be willing to wear a portable version of the system as part of your diabetes treatment at home?		☐	☐

27.15 Appendix 15

QUESTIONNAIRE FOR STAFF

Profession

- Nurse ☐
- Healthcare Assistant ☐
- Physician ☐

Staff Number:___________________________

		Using closed-loop	No difference	Using usual therapy
Q1.	**I spent less time managing diabetes (i.e giving injections, adjusting doses)**	☐	☐	☐
Q2.	**The glucose levels were more in control**	☐	☐	☐
Q3.	**The patient needed less treatment for hypoglycaemia**	☐	☐	☐
Q4.	**The patient needed less treatment for hyperglycaemia**	☐	☐	☐
Q5.	**I needed to contact the diabetes specialist team less often**	☐	☐	☐

Do you think that closed-loop insulin delivery could be used in the daily routine of hospitalized patients in the general wards Yes / No
(If No, please give reason) :

27.16 Appendix 16

Time schedule Visit 1/Visit 2

Approximate Hour	Running Time	Meals and Activities	Plasma Glucose Samples	Plasma Insulin Samples	Blood Volume
18:30 - 19:00		Arrival at CRF<br>Sensor insertion			
19:30		Weight and height<br>Cannulation			
20:00		1st sensor calibration<br>Start Fully CL			
21:00		2nd sensor calibration			
07:00	0 min	3rd sensor calibration<br>Baseline sampling (only V1)<br>Breakfast (50g CHO)	✓	✓	3ml
07:15	15 min		✓	✓	2ml
07:30	30 min		✓	✓	3ml
07:45	45 min		✓	✓	2ml
08:00	60 min		✓	✓	3ml
08:15	75 min		✓	✓	2ml
08:30	90 min		✓	✓	3ml
08:45	105 min		✓		2ml
09:00	120 min		✓	✓	3ml
09:15	135 min		✓		2ml
09:30	150 min		✓	✓	3ml
09:45	165 min		✓	✓	2ml
10:00	180 min		✓	✓	3ml
10:30	210 min		✓	✓	2ml
11:00	240 min		✓	✓	2ml
11:30	270 min		✓	✓	2ml
12:00	300 min	Lunch (80g of CHO)	✓	✓	3ml
12:15	315 min		✓	✓	2ml
12:30	330 min		✓	✓	3ml
12:45	345 min		✓		2ml
13:00	360 min		✓	✓	3ml
13:15	375 min		✓		2ml
13:30	390 min		✓	✓	3ml
13:45	405 min		✓	✓	2ml

14:00	420 min		✓	✓	3ml
14:15	435 min		✓	✓	2ml
14:30	450 min		✓	✓	3ml
14:45	465 min		✓	✓	2ml
15:00	480 min		✓	✓	3ml
15:30	510 min		✓	✓	2ml
16:00	540 min		✓	✓	2ml
16:30	570 min		✓	✓	2ml
17:00	600 min		✓	✓	2ml
17:30	630 min	Remove cannulas<br>Remove study devices<br>Administer long-acting insulin			
18:00	660 min	Discharge			

Angie02 phase 3: An open-label, two-centre, randomised, parallel design study to assess the efficacy and safety of fully automated closed-loop glucose control in comparison with conventional treatment in hospitalized patients receiving parenteral and/or enteral nutrition

Statistical Analysis Plan

Version 1.0 Date 17 September 2018

1 Study Overview

Angie02 phase 3 study adopts an open-label, randomised, two-arm, parallel design. The main objective of the current study is to determine the efficacy and safety of automated closed-loop glucose control system in hospitalised patients in non-critical care setting requiring subcutaneous insulin and parenteral/enteral nutrition support, as compared to usual care.

2 Objectives

2.1 Efficacy

To assess the efficacy of automated closed-loop (CL) glucose control in the general ward setting in maintaining CGM glucose levels within the target range (5.6 to 10.0 mmol/l) compared to conventional glucose control in hospitalised patients treated with subcutaneous insulin therapy whilst receiving parenteral/enteral nutrition support.

2.2 Safety

To determine the impact of automated closed-loop glucose control in acute, non-critical care settings in terms of incidence of severe hypoglycaemia (defined as capillary glucose levels below 2.2 mmol/l or an event requiring assistance of another person) and occurrence of other adverse events. In addition, the number of events with capillary glucose level > 20mmol/l will be assessed.
Number of treated hypoglycaemic events will be documented.

3 Sample Size

Up to a total of 50 participants will be randomised to allow for 40 participants with at least 48 hours data available for assessment (20 participants per centre). As the previous phase 1 and phase 2 of

the study may not provide reliable information about the within group variability in this particular population receiving parenteral and/or enteral nutrition, no formal power calculation is applicable.

4 Analysis Plan

4.1 Analysable period

Efficacy data will be analysed according to the intention-to-treat principle. Analysis will be performed from the first available sensor reading until discharge or at 15 days, whichever occurs soonest. In the event of closed-loop operation being suspended for more than 4 hours for non-device related issues such as when participant is undergoing surgery managed using conventional subcutaneous or intravenous insulin therapy or control patients being switched to intravenous insulin, data during that period will be excluded from the analysis. The number of such periods will be reported.
Data from all randomised participants with or without protocol violation including dropouts and withdrawals will be included in the analysis.

4.2 Primary Analysis

The primary outcome measure is time spent with CGM glucose concentration in the target range (5.6-10.0mmol/L) during the study period.

The primary analysis will be the single comparison of above outcome between closed-loop and conventional glucose control. Unpaired t-test will be used to compare normally distributed variables. Non-normally distributed variables will be compared using Mann-Whitney U-test.

Values will be given mean (standard deviation) or as median (interquartile range) for each treatment (CL or conventional glucose control).

4.3 Secondary efficacy outcomes

The following outcomes on the two treatment arms will be compared. As appropriate, glucose outcomes will be calculated using CGM values or point of care capillary glucose (Novostat or similar).

1. The AUC below 3.5mmol/l based on CGM
2. The AUC below 3.0mmol/l based on CGM
3. Time spent with glucose levels < 5.6 mmol/l based on CGM
4. Time spent with glucose levels in hypoglycaemic range < 3.9 mmol/L based on CGM
5. Time spent with glucose levels in hypoglycaemic range < 3.0 mmol/L based on CGM
6. Time spent with glucose levels in hypoglycaemic range < 2.8 mmol/L based on CGM
7. Time spent with glucose levels in hyperglycaemic range (> 10.0 mmol/L) based on CGM

8. Time spent with glucose levels in significant hyperglycaemic range (> 20.0 mmol/L) based on CGM
9. Mean of CGM glucose levels.
10. SD and CV of CGM glucose levels.
11. Total daily insulin dose
12. Between 24 hour period variability: Coefficient of variation of CGM glucose between 24 hour periods (08:00 to 08:00)
13. Number of capillary glucose (Novostat or similar) confirmed hypoglycaemic events <3.5mmol/l
14. Pre-breakfast, pre-lunch, pre-dinner, and evening capillary glucose values (Novostat or similar)

During overnight period between 24:00 and 08:00 (8 hours) the following outcomes will be calculated:

- Time spent with CGM glucose concentration in the target range (5.6-10.0mmol/L)
- Mean CGM glucose levels
- The AUC below 3.5mmol/l based on CGM
- SD and CV of CGM glucose levels
- Between night variability: Coefficient of variation of CGM glucose between nights
- Total insulin dose during (CL only)

During day period between 08:00 to 24:00 (16 hours) the following outcomes will be calculated:

- Time spent with CGM glucose concentration in the target range (5.6-10.0mmol/L)
- Mean CGM glucose levels
- The AUC below 3.5mmol/l based on CGM
- SD and CV of CGM glucose levels
- Between day-time variability: Coefficient of variation of CGM glucose between days
- Total insulin dose during (CL only)

Nutrition-related information
The following information related to the nutrition during the study will be analysed

- Total number of patients receiving
    - Parenteral nutrition only
    - Enteral nutrition only
    - Both, parenteral and enteral nutrition
- Number of study days on nutrition support (parenteral and/or enteral nutrition)
- Total daily carbohydrate intake/supply (parenteral + enteral + oral carbohydrates)
- Between day-time variability of total carbohydrate intake/supply

4.4 Safety Analysis

Safety data including number of subjects and number of occurrences of severe hypoglycaemia events (capillary glucose <2.2mmol/l, or requiring assistance of another person), significant hyperglycaemic events (>20mmol/l), events with capillary glucose >18mmol/l for >1h in the closed-loop group (requiring correctional insulin by injection), hyperglycaemia with ketonaemia (B-OHB >0.6mmol/l) as well as number of adverse events and including SADEs and SAEs will be tabulated for all subjects.

4.5 Utility outcomes

Time when closed-loop system is in operation will assess utility. Time when sensor glucose was available will be calculated.

4.6 Participant feedback questionnaires

Closed-Loop participant feedback questionnaires will be collected and used for descriptive statistics.
